# The effect of adjuvant oral application of honey in the management of postoperative pain after tonsillectomy in adults: A pilot study

**DOI:** 10.1371/journal.pone.0228481

**Published:** 2020-02-10

**Authors:** Katharina Geißler, Margaretha Schulze, Johanna Inhestern, Winfried Meißner, Orlando Guntinas-Lichius

**Affiliations:** 1 Department of Otorhinolaryngology, Jena University Hospital, Jena, Germany; 2 Department of Anaesthesiology and Intensive Care Medicine, Jena University Hospital, Jena, Germany; University of Bern, SWITZERLAND

## Abstract

**Objective:**

To analyze the effect of adjuvant oral application of honey for treating postoperative pain after tonsillectomy.

**Design:**

Single centre prospective cohort study.

**Setting:**

Two cohorts of patients after tonsillectomy.

**Participants:**

56 patients treated with honey 8 times per day (honey group), 18 patients treated without honey (control group); baseline analgesia were non-steroidal anti-inflammatory drugs (NSAID) or coxibs; opioids were used as pro re nata (PRN) medication; mean age 34.4 ± 13.4 years; 36% women.

**Main outcome measures:**

On first to fifth postoperative day, patients rated their pain using the validated questionnaire of the German-wide project Quality Improvement in Postoperative Pain Treatment (QUIPS) including a numeric rating scale (NRS, 0–10) for determination of patient's pain. QUIPS allows standardized assessment of patients' characteristics andpain-associated patient-reported outcomes (PROs). The influence of preoperative and postoperative parameters on patients' postoperative pain were estimated by univariate and multivariate statistical analysis.

**Results:**

Average pain in activity in the control group was greater than 4 (NRS 4.4 ± 2.4) during the first five postoperative days, with a renewed increase in pain intensity on the fifth day (4.3 ± 2.5). In the honey group, the pain in activity decreased without any further pain increase and was only higher than 4 on the first three postoperative days (4.3 ± 2.1, all p>0.05). However; neither minimal nor maximal pain were significantly different between both groups on the first postoperative day (p = 0.217, p = 0.980). Over the five postoperative days, the minimal and maximal pain in the honey group decreased continuously and faster than in the control group. With regard to pain-related impairments on the first day, the honey group reported less pain-related sleep disturbance (p = 0.026), as well as significantly fewer episodes of postoperative oral bleeding (p = 0.028) than the control group. Patients without honey consumption had on the first and fifth postoperative day a higher risk of increased minimal pain (OR = -2.424, CI = -4.075 –-0.385). Gender was an independent factor for compliance of honey consumption on the second postoperative day (p = 0.037). Men had a lower probability for compliance of honey consumption (OR = -0.288, CI = -2.863 –-0.090).

**Conclusion:**

There was a trend of reduced postoperative pain after oral honey application. Honey also seems to reduce pain-related impairments. The need for additional opioids on the first day could be reduced. A larger controlled trial is now needed to varify the effect of honey on pain after tonsillectomy.

**Clinical trial registration number:**

German Clinical Trials Register DRKS00006153. The authors confirm that all ongoing and related trials for this drug/intervention are registered.

## Introduction

Tonsillectomy, the surgical removal of the palatine tonsils, is still one of the most common surgical procedures in adults in Germany and around the world [1]. Tonsillectomy causes severe postoperative pain lasting for many days [2]. A prospective cohort study taking part in the Quality Improvement in Postoperative Pain Treatment (QUIPS) registry has shown that tonsillectomy was one of the most painful surgical procedures even compared to major surgery procedures [3]. For pain therapy in adults after tonsillectomy typically non-opioid analgesics are used, often combined with an opioid on demand [4, 5, 6, 7].

Even though patients receive such a combination therapy postoperative pain after tonsillectomy remains at a high level and an improved therapy plan is needed [8, 9, 10].

Different factors contribute to postoperative pain after tonsillectomy: the dense innervation with pain fibers in the area of tonsils [11], mediators of inflammation like bradykinin or prostaglandins, which irritate sensitive nerve endings and induce strong pain [12, 13], local infiltration of neutrophile granulocytes and cytokines [14]. Local application of honey reduces redness and swelling of infected wounds and reduces healing time. Honey seems to have a comparable effect to topic antibiotics on pathogenic bacterial infections of surgical wounds and conjunctiva [15]. Honey might affect the local inflammation and thereby reduce und diminish duration of wound pain. The studies performed so far studying the effect of honey on postoperative pain after tonsillectomy revealed inconsistent results or did not used standard pain outcome measures [16, 17, 18, 19, 20, 21, 22, 23, 24, 25].

The above mentioned project QUIPS was developed in 2005, consisting of standardized data acquisition and an analysis of quality and process indicators [26]. QUIPS and the international counterpart PAIN OUT are open for every hospital worldwide and are web-based [9, 27, 28]. The present prospective clinical study used QUIPS data to analyze if the additional oral application of honey several times a day reduces postoperative pain better than traditional pain therapy with a non-opioid (metamizole or etoricoxib), in combination with an opioid as PRN medication.

## Methods

The present prospective cohort study was part of the German-wide Quality Improvement in Postoperative Pain Treatment (QUIPS) registry (German Clinical Trials Register DRKS00006153, registration in May 2014). Institutional review board approval was obtained prior to study initiation in November 2012 by the Ethics Committee of the Jena University Hospital, Thuringia, Germany. For enrollment of participants in 2012 a positive approval by Ethics Committee was sufficient. Therefore there was a delay in registration the study. The authors confirm that all ongoing and related trials for this drug/intervention are registered.

## Subjects

The patients in the ENT department of Jena University Hospital had to fulfill the following inclusion criteria: age of 18 years and older, oriented and awake, linguistic and intellectual understanding, written consent, bilateral tonsillectomy, diagnosis of acute recurrent tonsillitis or chronic tonsillitis, peritonsillar abscess, obstructive sleep apnea or tumor of tonsil.

Exclusion criteria were age younger than 18 years, cognitive deficits, limited communication skills, diabetes mellitus, no written consent, tonsillectomy one side, postoperative admission on intensive care and previous allergic reaction to honey.

**Fig 1:**
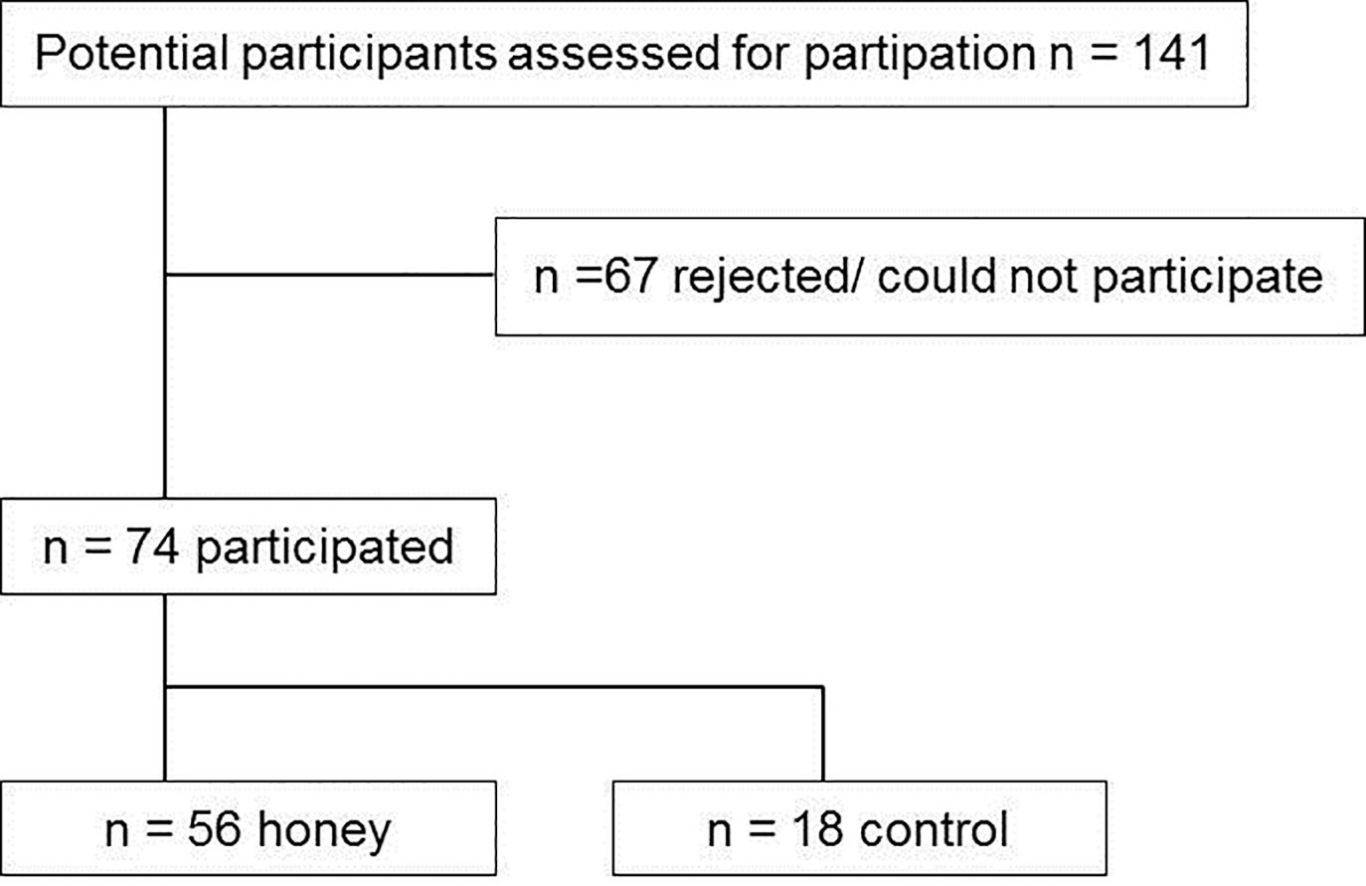
Enrolled patients.

Patients treated in the hospital as in-patients between December 2015 and March 2017 (honey group) and February 2013 and November 2013 (control group) were included by self-selection. The time for patient recruitment and follow-up was between February 2013 and April 2017. There were no changes in pain protocol between 2013 and 2017. 141 tonsillectomies were performed in patients with 18 years and older. 74 patients participated, 67 patients rejected or could not participate (Fig 1).

The addition of honey to patients`food was introduced as part of the clinical routine. Patients received eight pots of honey of nectar of different blossoms (Blütenhonig, Transgourmet Deutschland, Riedstadt, Germany) with a content of 20g per day (maximum 160g per day). The honey was delivered in sterile packages. The dose of honey was chosen empirically. There is no reported dose related toxicity to honey for adults. An illustrated information brochure was handed to the patient. The honey should be applied into the mouth with a spoon and then distributed in the oral cavity for about five minutes. If the honey's sweetness made it impossible for the patient to apply honey this way, it was allowed to dissolve the honey in a half-filled cup with lukewarm water. The honey solution was then also applied over five minutes in the oral cavity. The empty honey pots were counted. The compliance of daily honey consumption was measured for all patients as the days on which all 8 honey pots were used.

### Pain and pain management measures

The QUIPS questionnaires are presented in detail elsewhere [10]. Briefly, the QUIPS questionnaires consisted of two parts for each patient: This first part was covering the patient-reported outcome (PRO) parameters of the questionnaire, whereas the second part was filled by the investigator. After a standardized instruction, the patient itself completed the part one of the form. The patients received a validated 15-item QUIPS questionnaire from first till fifth postoperative day. QUIPS used 11-point numeric rating scales (NRS) to estimate the patient’s pain during activities, maximal pain and pain at rest. Generally, higher numbers are indicating more pain (0 = no pain; 10 = most imaginable pain). Furthermore, the patient was asked by dichotomized (yes/no) questions about pain-related impairments (mobility, breathing, sleep, mood), side effects of pain treatment (drowsiness, nausea, vomiting), and satisfaction with the pain management. The patients were also asked about the preoperative pain counselling in three categories (yes, in general; yes, specific; no). General pain counselling meant that education about postoperative pain and its management in general was part of the pre-surgical interview with the patients. Specific pain counselling assumed that it was talked about specific pain related to the surgical procedure the patient underwent. Furthermore, the interview had to include education on specific measures to prevent and manage postoperative pain before, during and after tonsillectomy exactly for the interviewed patient. The second part, which is filled by the investigator, was covering the relevant demographic and clinical parameters like age, gender, type of surgery, anaesthesia, and pain management.

### Statistical analysis

IBM SPSS statistics software (Version 23.0.0.0) was used. Data is presented as mean ± standard deviation (SD) if not otherwise indicated. Clinical and outcome parameters of all patients were summarized descriptively. To study the metric data of two independent groups the nonparametric Mann-Whitney-U-test was used. If there were variables with more than 2 possible answers, they were analyzed by the nonparametric Kruskal-Wallis-ANOVA-test. To check for significance in nominal variables Pearson`s chi-square test was applied. For the comparison of dependent data, the nonparametric Wilcoxon-test was used. The significance level was set at p<0.05. Multivariate ordinal regression was used to analyze predictors for more postoperative pain for all significant parameters from the univariate analysis. Multivariable binary logistic regression models with stepwise entry were used for the dichotomized categorized outcome parameters to analyze the association to pain-related interferes and pain therapy side effects. In general, nominal p values of two-tailed tests are reported. Pain in activity was analysed with a repeated measures ANOVA with the within factor SESSION (five levels: first to fifth postoperative day) and the between factor GROUP.

## Results

### Demographic parameters

An overview on the comparison of the demographic and baseline parameters is given in **Table 1**. The mean age of the patients was 34.4 ± 13.4 years. In both patient groups, the proportion of male patients was about two-thirds. Patients’ characteristics were not different between both groups (all p>0.05). Postoperative bleeding, with or without need of surgery, on the first five days after surgery was more frequent in the control group (p = 0.028).

**Table 1:** Demographic parameters.

Parameter	honey group n = 56	control group n = 18	p-value
Gender			0.416
female	22	5	
Male	34	13	
age, years	35.8 ± 13.9	30.1 ± 11.2	0.172
Diagnosis			0.383
chronic tonsillitis	24	10	
peritonsillar abscess	24	8	
obstructive sleep	6	0	
apnoe			
tumor of tonsil	2	0	
postoperative complications			**0.028**
No	54	14	
Bleeding	2	4	
ASA-classification			0.283
I	32	13	
II and III	24	5	

ASA = American Society of Anesthesiologists

The highest compliance of honey consumption (% of patients applying all 8 honey pots) was measured on the first and second postoperative day. More than 90% of the patients in the honey group used all 8 honey pots (first day 94.6%, second day 91.1%, third day 87.5%, fourth day 80.4% and fifth day 76.8%).

### Postoperative results of QUIPS

The postoperative pain in activity was recorded in honey and control group for five days. Maximum and minimum pain was asked on the first postoperative day. The pain in activity decreased in both groups on the fourth postoperative day. On the fifth postoperative day pain in activity increased in the control group (4.3 ± 2.5), but not in the honey group (3.3 ± 2.2, p = 0.128, Table 2).

**Table 2:** Pain in activity in both groups on the first to fifth postoperative day.

postoperative day	honey group	control group	p-value
1	4.6 ± 2.3	5.0 ± 2.3	0.529
2	4.6 ± 2.3	4.4 ± 2.3	0.854
3	4.1 ± 2.1	4.2 ± 2.4	0.806
4	3.6 ± 2.1	4.0 ± 2.5	0.536
5	3.3 ± 2.2	4.3 ± 2.5	0.128
5–1	-1.3 ± 0.1	-0.7 ± 0.2	0.458

**Fig 2:**
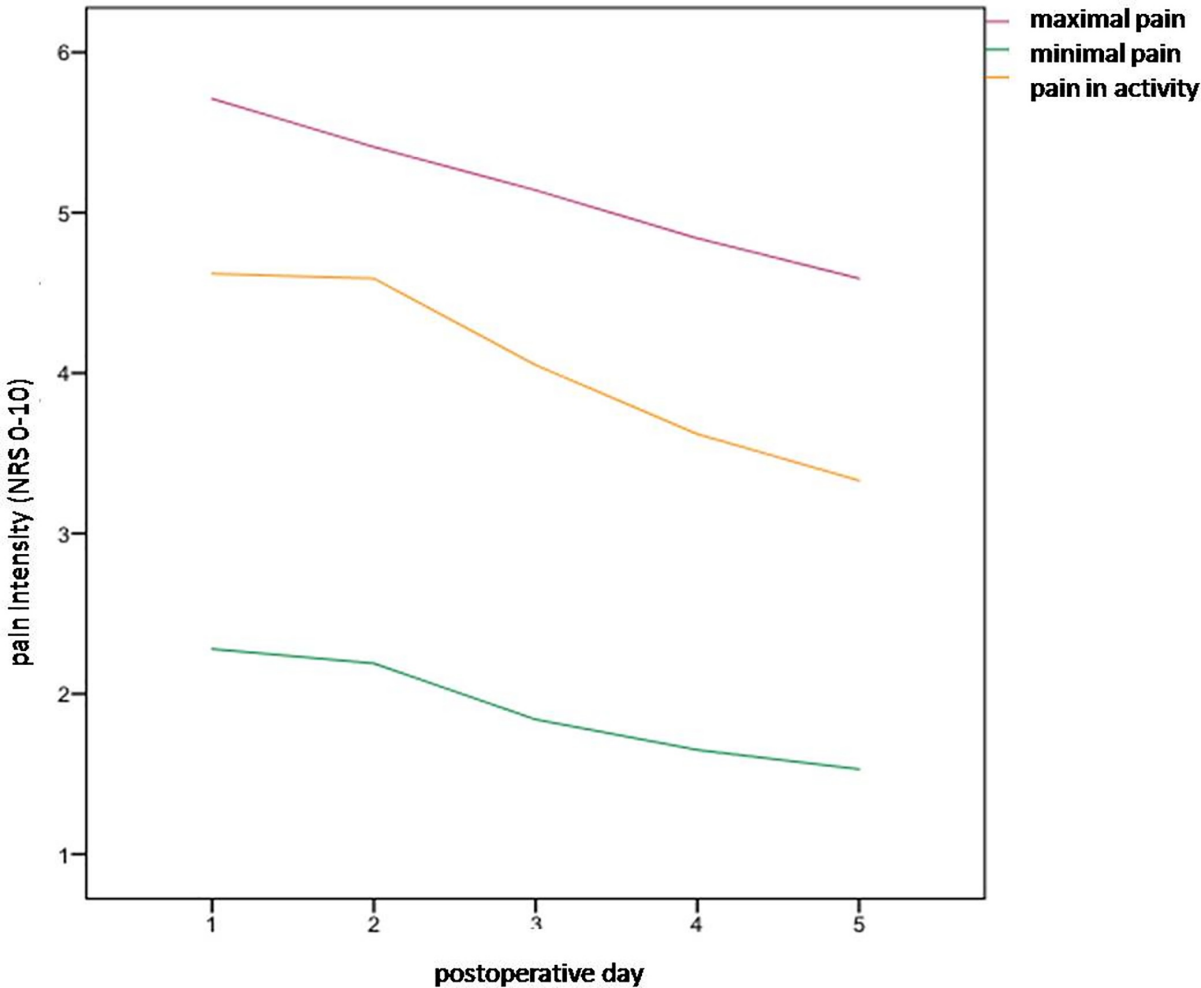
Pain in activity, maximal and minimal pain in honey group.

The maximum and minimum pain on the first postoperative day was not significant different in both groups (p = 0.980, p = 0.217). In the honey group pain in activity, maximal and minimal pain continuously decreased over first to fifth postoperative day (Fig 2). In both groups over 90% of patients received a general or special medical education about pain therapy (p = 0.887).

Regarding the repeated measures ANOVA there was a main effect for SESSION (F(4.272) = 7.85; p<0.001; partial Eta Squared = 0.103). There is a constant decrease of pain in activity across postoperative days. There was no interaction between GROUP and SESSION (F(4.272) = 1.69; no signifance) and no effect for GROUP (F(1.68) = 0.27; no significance).

### Postoperative pain and treatment-associated impairments

52% of patients in honey group woke up during the night due to pain, compared to 83% in the control group (p = 0.026, Table 3). The satisfaction with pain therapy on the first postoperative day was indifferent between both groups (p = 0.453).

**Table 3:** Results of QUIPS questionnaire on the first postoperative day.

Parameter	honey group n = 56	control group n = 18	p-value
medical education			0.887
yes, general	41	14	
yes, special	10	3	
no	5	1	
preoperative chronic pain	11	6	
	Mean ± SD 0.7 ± 0.5	Mean ± SD 6.8 ± 1.7	**<0.001**
pain on first postoperative day			
pain in activity	4.6 ± 2.3	5.0 ± 2.3	0.529
maximal pain	5.8 ± 2.3	5.9 ± 1.9	0.980
minimal pain	2.3 ± 1.7	1.8 ± 1.8	0.217
pain-associated and pain therapy-associated impairments on first postoperative day			
pain while breathing			1.000
yes	38	13	
no	18	5	
waking up because of pain			**0.026**
yes	29	15	
no	27	3	
Fatigue			1.000
yes	26	9	
no	30	9	
feeling uncomfortable because of pain			0.785
yes	22	8	
no	34	10	
complaining by mobility restricts			0.372
yes	16	3	
no	40	15	
Nausea			0.102
yes	9	0	
no	47	18	
Vomitus			-
yes	0	0	
no	56	18	
desire for pain killers			1.000
yes	10	3	
no	46	15	
satisfaction with pain therapy	Mean ± SD 11.6 ± 2.5	Mean ± SD 11.2 ± 2.5	0.453

SD = standard deviation

**Fig 3:**
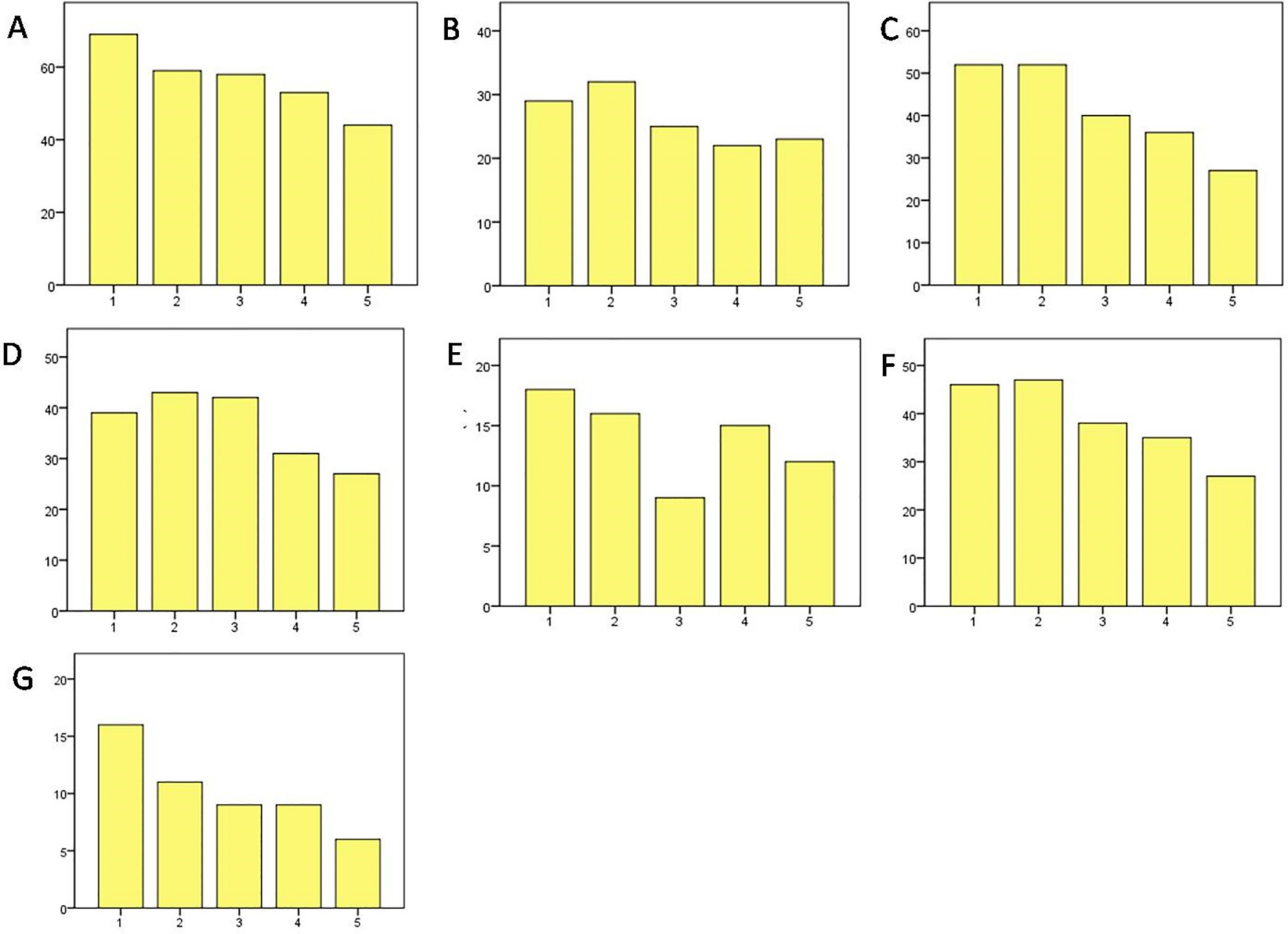
A–impairment whilst breathing (%), B–impairment in mobility (%), C–waking up due to pain (%), D–feeling uncomfortable because of pain (%), E–desire for more painkiller (%), F–fatigue (%), G–nausea (%), y-axis–postoperative impairments (%), x-axis–postoperative day, results of honey group.

The postoperative impairments in the honey group decreased throughout the first till the fifth postoperative day with the strongest decrease concerning the complaints of pain whilst breathing and waking up in the night due to pain (Fig 3).

### Process parameter

All interventions were performed in general anesthesia, without local anesthesia. Intraoperatively two third of the honey group and one third of the control group received the highly potent and short acting opioid remifentanil (p = 0.058, Table 4). Half of the patients received an intravenous application of piritramid in the recovery room (p = 1.000, Table 4). On ward, a majority of the honey group received metamizole as basic analgesia on the first postoperative day, in the control group only one third. The other patients took etoricoxib (p<0.001). In the honey group 96% of patients received no additional non-opioid, in the control group nearly the half of patients additional metamizole (p<0.001). On the first postoperative day more than twice as much patients in the control compared to the honey group requested opioids (p = 0.004). In the honey group mostly tramadol, in the control group mostly piritramid were applied (p<0.001).

**Table 4:** Process parameter on the first postoperative day.

parameter	honey group n = 56	control group n = 18	p-value
preoperative			
regular intake of pain killers			0.434
yes	9 (16.1%)	1 (5.6%)	
no	47 (83.9%)	17 (94.4%)	
pain therapy			1.000
yes	3 (5.4%)	0	
no	53 (94.6%)	18 (100%)	
most used sedativum			0.115
no	4 (7.1%)	0	
midazolam	43 (76.8%)	17 (94.4%)	
clorazepat	1 (1.8%)	0	
Intraoperative			
remifentanil			0.058
yes	34 (60.7%)	6 (33.3%)	
no	22 (39.3%)	12 (66.7%)	
first postoperative day			
opioid in recovery room			1.000
yes (piritramid)	29 (51.8%)	9 (50%)	
no	27 (48.2%)	9 (50%)	
opioid on ward			**0.004**
yes	16 (28.6%)	13 (72.2%)	
no	39 (69.6%)	5 (27.8%)	
most used opioid on ward			**<0.001**
no	39 (69.6%)	5 (27.8%)	
tramadol	10 (17.9%)	1 (5.6%)	
piritramid	4 (7.1%)	12 (66.7%)	
tapentadol	2 (3.6%)	0	
additional opioid on ward			**0.046**
yes	1 (1.8%)	3 (16.7%)	
no	54 (96.4%)	15 (83.3%)	
most used non-opioid on ward			**<0.001**
No	2 (3.6%)	0	
metamizole	53 (94.6%)	6 (33.3%)	
acetaminophen	1 (1.8%)	0	
etoricoxib	0	12 (66.7%)	
additional non-opioid on ward			**<0.001**
no	54 (96.4%)	8 (44.4%)	
metamizole	1 (1.8%)	1 (5.6%)	
acetaminophen	1 (1.8%)	8 (44.4%)	
etoricoxib	0	1 (5.6%)	

The changes of process parameters are described in detail for the honey group (Table 5). The highest demand after opioids on ward was on the second postoperative day.

**Table 5:** Process parameter on the first to fifth postoperative day in honey group.

Parameter	postoperative day				
	1	2	3	4	5
postoperative					
opioid on ward					
yes	16	22	15	10	7
no	39	33	40	45	48
most used opioid on ward					
no	39	33	40	45	48
tramadol	10	18	13	7	7
piritramid	4	3	0	1	0
tapentadol	2	1	1	2	0
codeine	0	0	1	0	0
additional opioid on ward					
yes	1	2	0	3	0
no	54	53	55	52	55
most used non-opioid on ward					
no	2	0	1	6	6
metamizole	53	54	53	48	48
acetaminophen	1	1	1	1	1
etoricoxib	0	1	1	1	1
additional non-opioid on ward					
no	54	53	55	55	54
ibuprofen	1	2	1	1	1
metamizole	1	1	0	0	0
indomethacin	0	0	0	0	1

### Demographic parameters associated with PROs

With univariate analysis the influence of demographic parameters on QUIPS results were analyzed. On the second and fifth postoperative days women had more pain as men (p = 0.024, p = 0.025). On the second postoperative day patients with tumor of tonsil or acute recurrent tonsillitis had more pain than patients with obstructive sleep apnea or peritonsillar abscess (p = 0.018, S1 Table). On the first and second postoperative day patients with obstructive sleep apnea had the strongest and patients with peritonsillar abscess the weakest maximal pain (p = 0.004, p = 0.039, S2 Table). Women had on the first to fifth postoperative day significant higher minimal pain in comparison to men (p = 0.002 to p = 0.021). On the first postoperative day patients without honey consumption had twice as high minimal pain (p = 0.023), on the fifth postoperative day still significant higher minimal pain (p = 0.049, S3 Table).

### Influence of process parameter on QUIPS results

In general, application of intraoperative remifentanil and/or postoperative opioids on demand was associated with higher pain scores on some postoperative days (see S4 to S6 Tables).

### Multivariate analysis of factors influencing postoperative pain in honey group

The diagnosis was an independent factor on pain in activity on the second postoperative day (p = 0.004): Patients with peritonsillar abscess had less pain in activity. Gender and honey consumption were independent factors on minimal pain on the first and fifth postoperative day (p = 0.016, p = 0.019, p = 0,023). On the first postoperative day women had a more two times higher risk of pain (OR = 2.491, CI = 0.206–1.907). Patients without honey application had a higher risk of pain on the first and fifth postoperative day (OR = -2.424, CI = -4.075 –-0.385, Table 6).

**Table 6:** Independent factors with influence on postoperative pain.

pain in activity on second postoperative day R^2^ = 0.241, p = 0.002	OR	95% CI lower limit	95% CI upper limit	p-value
gender (male = 0, female = 1)	0.214	-0.233	2.096	0.114
diagnosis (acute recurrent tonsillitis = 0, peritonsillar abscess = 1)	-0.399	-2.878	-0.567	**0.004**
minimal pain on first postoperative day R^2^ = 0.181, p = 0.005				
gender (male = 0, female = 1)	2.491	0.206	1.907	**0.016**
honey consumption (no = 0, yes = 1)	-2.424	-4.075	-0.385	**0.019**
minimal pain on fifth postoperative day R^2^ = 0.286, p<0.001				
gender (male = 0, female = 1)	0.331	0.176	1.656	**0.016**
honey consumption (no = 0,yes = 1)	-0.313	-2.267	-0.178	**0.023**
satisfaction on first postoperative day R^2^ = 0.155, p = 0.096				
age in years (<33.5 = 0, >33.5 = 1)	-0.015	-1.500	1.352	0.917
diagnosis (acute recurrent tonsillitis = 0, peritonsillar abscess = 1)	0.250	-0.100	2.621	0.069

R^2^ = coefficient of determination, CI = confidence interval, OR (Odds-Ratio) = standardized coefficient

Diagnosis was an independent factor for satisfaction with pain therapy on second to fifth postoperative day (p = 0.009 to p = 0.044). Patients with acute recurrent tonsillitis had a higher risk of less satisfaction on second to fifth postoperative day (Table 7).

**Table 7:** Independent factors with influence on satisfaction with pain therapy.

satisfaction on second postoperative day R^2^ = 0.403, p = 0.002	OR	95% CI lower limit	95% CI upper limit	p-value
age in years (<33.5 = 0, >33.5 = 1)	-0.094	-2.217	1.239	0.569
diagnosis (acute recurrent tonsillitis = 0, peritonsillar abscess = 1)	0.434	0.589	3.917	**0.009**
satisfaction on third postoperative day R^2^ = 0.314, p = 0.007				
age in years (<33.5 = 0, >33.5 = 1)	0.048	-1.449	1.945	0.769
diagnosis (acute recurrent tonsillitis = 0, peritonsillar abscess = 1)	0.353	0.052	3.574	**0.044**
satisfaction on fourth postoperative day R^2^ = 0.329, p = 0.002				
diagnosis (acute recurrent tonsillitis = 0, peritonsillar abscess = 1)	0.402	0.529	3.490	**0.009**
satisfaction on fifth postoperative day R^2^ = 0.277, p = 0.011				
diagnosis (acute recurrent tonsillitis = 0, peritonsillar abscess = 1)	0.414	0.492	4.023	**0.014**

R^2^ = coefficient of determination, CI = confidence interval, OR (Odds-Ratio) = standardized coefficient

Gender was an independent factor for compliance of honey consumption on the second postoperative day (p = 0.037). Men had a lower probability for compliance of honey consumption (OR = -0.288, CI = -2.863 –-0.090), Table 8).

**Table 8:** Independent factors with influence on complicance of honey consumption.

honey consumption on second postoperative day R^2^ = 0.160, p = 0.017	OR	95% CI lower limit	95% CI upper limit	p-value
gender (male = 0, female = 1)	-0.288	-2.863	-0.090	**0.037**

R^2^ = coefficient of determination, CI = confidence interval, OR (Odds-Ratio) = standardized coefficient

Diagnosis was an independent factor on impairment of breathing on the second and fifth postoperative day (p = 0.006, p = 0.031) and on night pain on the fourth postoperative day (p = 0.022). On the second postoperative day patients with acute recurrent tonsillitis had an eightfold higher risk of impairment of breathing (OR = 8.575, CI = 1.839–39.994), on the fifth postoperative day a fourfold higher risk (OR = 4.222, CI = 1.139–15.644). On the fourth postoperative day patients with acute recurrent tonsillitis had a fourfold higher risk of night pain (OR = 4.581, CI = 1.248–16.816, Table 9).

**Table 9:** Independent factors with influence on pain or pain-therapy related impairments.

impairment of breathing on the second postoperative day R^2^ = 0.373, p<0.001	OR	95% CI lower limit	95% CI upper limit	p-value
age in years (<33.5 = 0, >33.5 = 1)	2.428	0.582	10.127	0.223
diagnosis (acute recurrent tonsillitis = 0, peritonsillar abscess = 1)	8.575	1.839	39.994	**0.006**
impairment of breathing on the fifth postoperative day R^2^ = 0.225, p = 0.017				
gender (male = 0, female = 1)	2.609	0.700	9.719	0.153
diagnosis (acute recurrent tonsillitis = 0, peritonsillar abscess = 1)	4.222	1.139	15.644	**0.031**
night pain on fourth postoperative day R^2^ = 0.210, p = 0.019				
gender (male = 0, female = 1)	2.034	0.567	7.291	0.276
diagnosis (acute recurrent tonsillitis = 0, peritonsillar abscess = 1)	4.581	1.248	16.816	**0.022**

R^2^ = coefficient of determination, CI = confidence interval, OR (Odds-Ratio) = standardized coefficient

Application of opioids on ward was an independent factor on impairments of mobility on the third postoperative day (p = 0.046) and night pain on the first and fifth postoperative day (p = 0.022, p = 0.036). The application of opioids correlated with higher risk of impairments of mobility and night pain (p = 0.046, p = 0.022, p = 0.036, Table 10).

**Table 10:** Influence of opioid use on ward on pain or pain-therapy related impairments.

impairment of mobility on third postoperative day R^2^ = 0.265, p = 0.005	OR	95% CI lower limit	95% CI upper limit	p-value
regular pain medication (no = 0, yes = 1)	0.227	0.044	1.166	0.076
opioids on ward (no = 0, yes = 1)	0.172	0.043	0,692	**0.046**
night pain on first postoperative day R^2^ = 0.336, p = 0.001				
opioids on ward (no = 0, yes = 1)	0.144	0.027	0.753	**0.022**
night pain on the fourth pain R^2^ = 0.257, p = 0.004				
opioids on ward (no = 0, yes = 1)	0.150	0.026	0.880	**0.036**

R^2^ = coefficient of determination, CI = confidence interval, OR (Odds-Ratio) = standardized coefficient

## Discussion

The present pilot study evaluated whether oral application of honey as adjuvant therapy after tonsillectomy in adult patients can reduce postoperative pain as well as postoperative pain- or treatment-related impairments. There was a trend of reduced pain after honey application as well as diminished pain-related impairments.

The strengths of this pilot study are the standardized assessment of process and outcome parameters with the QUIPS instrument [8] and repeated measurements from the first to the fifth postoperative day. The weaknesses are the lack of randomization and a low number of patients in the control group. This was a pilot study with exploratory character. Current data were not sufficient to define a hypothesis on the effect of honey on postoperative pain. Furthermore, the correct oral application of honey with regard to a 2-hour intake interval and a 5-minute residence time in the mouth in all cases cannot be guaranteed. It was only feasible to count the empty honey pots after application.

Studies investigating the effects of honey on tonsillectomy included Tualang honey from Southeast Asia [20, 18], unprocessed, pure honey from Malaysia [21], flower honey of thyme and tragacanth plants [16] and commercially available more or less undefined honey [17, 29]. Several studies described, that honey application, even on inflamed oral mucosa, is a painless procedure [30, 31]. The type of honey does not seem to have a significant impact on the results. The present study uses a standard honey (a blend of floral honey from EU and non-EU countries) provided by the University Hospital's kitchen for patient breakfast. The recommended frequency of oral honey application ranged from once [19] or three-times daily [20] to four-hourly [29] or even hourly intake during the waking phase [16]. Nevertheless, a clear recommendation for the application intervals cannot be deduced from these studies. Dosage and frequency of honey intake still has to be specifically investigated.

The multivariate analysis yielded an interesting result: Patients of the control group were about six times more likely to receive an opioid compared to patients in the honey group. Ingestion of another non-opioid on ward was more likely in patients of the control group. The lower demand for analgesics within the honey group may be related to lower postoperative pain. It is also possible that the patients on the first day waived additional analgesics, as they hoped for the pain-relieving effect of honey. However, based upon the available data this proposition cannot be verified.

In contrast to the honey group, other studies in children, adolescents and adults showed an increase in postoperative pain after tonsillectomy due to acute recurrent tonsillitis from the third postoperative day [32, 33]. Pain within the honey group decreased continuously without any further pain peaks, this could be a hint for accelerated wound healing by the applied honey.

In the pediatric study by Mohebbi et al., patients took much less painkillers when they took honey as the first on-demand medication [17]. The direct comparison with the pain values of other honey studies was not always possible because in the present study a more specific differentiation was made between maximal, minimal and pain in activity. In addition, the comparative studies had pediatric or mixed patient groups. Pain in the other studies in children was already below the intervention limit of NRS 4 [17] or even below 3.5 [16] on the first postoperative day, in contrast to the present study of more than 4 (pain in activity) and over 5 (maximal pain). The satisfaction with pain therapy was more than 11 out of 15 possible points on the first day after surgery in both groups. Despite some high pain values, the patients were satisfied with the pain therapy. This paradox appearing phenomenon has already been observed in several other studies [34, 35]. Even when pain relief was below preoperative expectations, patients are often satisfied with the pain therapy [36]. Not pain intensity or actual pain relief alone seem to be the key to patient satisfaction. Schwenkglenks et al. assumed a positive effect on satisfaction by including the patient in therapy planning and by their general well-being in the clinic [35].

A larger controlled trial is now needed to confirm the effect of honey on pain after tonsillectomy. In order to make a more precise assessment of the effectiveness of the honey, a better defined honey with standard composition might be helpful. The impact of the mode of administration as well as the amount and frequency of oral honey application on postoperative pain should also be analyzed in further studies.

## Conclusion

Based on the survey of postoperative minimal, maximal and pain in activity over five days, the present study showed that the pain therapy after tonsillectomy needs further improvement. Even with additional two-hourly intake of honey (eight times daily, 20 g each time), maximal pain with values above the intervention limit of NRS 4 on all five days as well as the pain in activity on the first three days were clearly too high. This pilot study suggests that oral application of honey might prevent a postoperative pain increase on postoperative day 4 and 5, have an opioid-sparing effect.

Overall, honey seems to have a healing and pain-relieving effect. The extent to which honey can be implemented in pain therapy regimes after tonsillectomy must be determined in further studies.

## Supporting information

S1 Checklist TREND statement checklist. (PDF)

S1 Table Influence of demographic parameters on pain in activity. (DOCX)

S2 Table Influence of demographic parameters on maximal pain. (DOCX)

S3 Table Influence of demographic parameters on minimal pain. (DOCX)

S4 Table Influence of process parameter on pain in activity. (DOCX)

S5 Table Influence of process parameter on maximal pain. (DOCX)

S6 Table Influence of process parameter on minimal pain. (DOCX)

S1 Fig (JPG)

S1 File (PDF)

S2 File (PDF)

S3 File (DOCX)
