# Supplementary material for: The effect of adjuvant oral application of honey in the management of postoperative pain after tonsillectomy in adults: A pilot study
Source: PLoS One. 2020 Feb 10;15(2):e0228481. doi: 10.1371/journal.pone.0228481 (PMC7010464; doi:10.1371/journal.pone.0228481)
Supplement: S1 Table — (DOCX) [file pone.0228481.s002.docx]

**S1 table** Influence of demographic parameters on pain in activity

| first postoperative day | Mean ± SD | p-value |
| --- | --- | --- |
| pain in activity | 4.6 ± 2.3 |  |
| age |  | 0.556 |
| <33.5 | 4.9 ± 2.0 |  |
| >33.5 | 4.4 ± 2.6 |  |
| gender |  |  |
| female | 4.3 ± 2.2 | 0.128 |
| male | 5.2 ± 2.3 |  |
| diagnosis |  | 0.278 |
| acute recurrent tonsillitis | 5.2 ± 1.9 |  |
| peritonsillar abscess | 3.9 ± 2.3 |  |
| obstructive sleep apnea | 4.7 ± 3.4 |  |
| tonsil tumor | 5.5 ± 2.1 |  |
| ASA status |  | 0.900 |
| I | 4.6 ± 2.0 |  |
| II/ III | 4.6 ± 2.7 |  |
| honey |  | 0.069 |
| yes | 4.5 ± 2.3 |  |
| no | 7.0 ± 1.0 |  |
| second postoperative day |  |  |
| pain in activity | 4.6 ± 2.3 |  |
| age |  | 0.094 |
| <33.5 | 5.1 ± 1.9 |  |
| >33.5 | 4.1 ± 2.5 |  |
| gender |  | **0.024** |
| female | 5.4 ± 2.5 |  |
| male | 4.1 ± 2.0 |  |
| diagnosis |  | **0.018** |
| acute recurrent tonsillitis | 5.4 ± 1.9 |  |
| peritonsillar abscess | 3.5 ± 2.1 |  |
| obstructive sleep apnea | 5.0 ± 2.4 |  |
| tonsil tumor | 7.0 ± 4.2 |  |
| ASA status |  | 0.894 |
| I | 4.6 ± 2.0 |  |
| II/ III | 4.5 ± 2.6 |  |
| honey |  | 0.433 |
| yes | 4.5 ± 2.3 |  |
| no | 5.2 ± 2.6 |  |
| third postoperative day |  |  |
| pain in activity | 4.1 ± 2.1 |  |
| age |  | 0.785 |
| <33.5 | 4.2 ± 1.9 |  |
| >33.5 | 4.0 ± 2.3 |  |
| gender |  | 0.053 |
| female | 4.7 ± 2.4 |  |
| male | 3.6 ± 1.8 |  |
| diagnosis |  | 0.265 |
| acute recurrent tonsillitis | 4.5 ± 2.1 |  |
| peritonsillar abscess | 3.4 ± 1.9 |  |
| obstructive sleep apnea | 4.3 ± 2.3 |  |
| tonsil tumor | 5.5 ± 3.5 |  |
| ASA status |  | 0.763 |
| I | 4.0 ± 2.0 |  |
| II/ III | 4.2 ± 2.2 |  |
| honey |  | 0.215 |
| yes | 3.9 ± 2.0 |  |
| no | 5.3 ± 2.8 |  |
| fourth postoperative day |  |  |
| pain in activity | 3.6 ± 2.1 |  |
| age |  | 0.839 |
| <33.5 | 3.7 ± 1.8 |  |
| >33.5 | 3.5 ± 2.3 |  |
| gender |  | 0.194 |
| female | 4.1 ± 2.4 |  |
| male | 3.3 ± 1.8 |  |
| diagnosis |  | 0.428 |
| acute recurrent tonsillitis | 4.1 ± 1.9 |  |
| peritonsillar abscess | 3.1 ± 2.1 |  |
| obstructive sleep apnea | 3.8 ± 2.5 |  |
| tonsil tumor | 3.5 ± 3.5 |  |
| ASA status |  | 0.870 |
| I | 3.6 ± 1.9 |  |
| II/ III | 3.6 ± 2.4 |  |
| honey |  | 0.974 |
| yes | 3.6 ± 1.9 |  |
| no | 3.8 ± 2.9 |  |
| fifth postoperative day |  |  |
| pain in activity | 3.3 ± 2.2 |  |
| age |  | 0.766 |
| <33.5 | 3.3 ± 2.2 |  |
| >33.5 | 3.4 ± 2.3 |  |
| gender |  | **0.025** |
| female | 4.2 ± 2.4 |  |
| male | 2.8 ± 1.9 |  |
| diagnosis |  | 0.426 |
| acute recurrent tonsillitis | 3.8 ± 2.2 |  |
| peritonsillar abscess | 2.7 ± 1.9 |  |
| obstructive sleep apnea | 3.5 ± 2.7 |  |
| tonsil tumor | 4.3 ± 4.6 |  |
| ASA status |  | 0.250 |
| I | 3.0 ± 1.9 |  |
| II/ III | 3.8 ± 2.5 |  |
| honey |  | 0.431 |
| yes | 3.1 ± 1.9 |  |
| no | 4.2 ± 3.3 |  |
