# Supplementary material for: The effect of adjuvant oral application of honey in the management of postoperative pain after tonsillectomy in adults: A pilot study
Source: PLoS One. 2020 Feb 10;15(2):e0228481. doi: 10.1371/journal.pone.0228481 (PMC7010464; doi:10.1371/journal.pone.0228481)
Supplement: S2 Table — (DOCX) [file pone.0228481.s003.docx]

**S2 table** Influence of demographic parameters on maximal pain

| first postoperative day | Mean ± SD | p-value |
| --- | --- | --- |
| maximal pain | 5.8 ± 2.3 |  |
| age |  | 0.450 |
| <33.5 | 6.1 ± 1.6 |  |
| >33.5 | 5.5 ± 2.7 |  |
| gender |  |  |
| female | 6.3 ± 2.3 | 0.194 |
| male | 5.5 ± 2.2 |  |
| diagnosis |  | **0.004** |
| acute recurrent tonsillitis | 6.5 ± 1.7 |  |
| peritonsillar abscess | 4.6 ± 2.2 |  |
| obstructive sleep apnea | 7.7 ± 1.0 |  |
| tonsil tumor | 6.5 ± 5.0 |  |
| ASA status |  | 0.535 |
| I | 5.6 ± 2.1 |  |
| II/ III | 6.0 ± 2.4 |  |
| honey |  | 0.062 |
| yes | 5.7 ± 2.2 |  |
| no | 8.0 |  |
| second postoperative day |  |  |
| maximal pain | 5.7 ± 2.3 |  |
| age |  | 0.148 |
| <33.5 | 6.2 ± 1.6 |  |
| >33.5 | 5.2 ± 2.8 |  |
| gender |  | 0.113 |
| female | 6.3 ± 2.5 |  |
| male | 5.3 ± 2.8 |  |
| diagnosis |  | **0.039** |
| acute recurrent tonsillitis | 6.5 ± 1.9 |  |
| peritonsillar abscess | 4.6 ± 2.3 |  |
| obstructive sleep apnea | 6.7 ± 2.0 |  |
| tonsil tumor | 6.5 ± 5.0 |  |
| ASA status |  | 0.745 |
| I | 5.7 ± 2.2 |  |
| II/ III | 5.8 ± 2.5 |  |
| honey |  | 0.933 |
| yes | 5.7 ± 2.3 |  |
| no | 5.6 ± 2.9 |  |
| third postoperative day |  |  |
| maximal pain | 5.4 ± 2.1 |  |
| age |  | 0.182 |
| <33.5 | 5.8 ± 1.9 |  |
| >33.5 | 5.0 ± 2.3 |  |
| gender |  | 0.060 |
| female | 6.0 ± 2.1 |  |
| male | 5.0 ± 2.1 |  |
| diagnosis |  | 0.086 |
| acute recurrent tonsillitis | 6.0 ± 2.1 |  |
| peritonsillar abscess | 4.7 ± 2.0 |  |
| obstructive sleep apnea | 6.5 ± 1.8 |  |
| tonsil tumor | 4.0 ± 2.8 |  |
| ASA status |  | 0.685 |
| I | 5.3 ± 2.3 |  |
| II/ III | 5.5 ± 1.9 |  |
| honey |  | 0.501 |
| yes | 5.3 ± 2.1 |  |
| no | 6.0 ± 2.4 |  |
| fourth postoperative day |  |  |
| maximal pain | 5.1 ± 2.3 |  |
| age |  | 0.273 |
| <33.5 | 5.5 ± 1.9 |  |
| >33.5 | 4.8 ± 2.6 |  |
| gender |  | 0.155 |
| female | 5.7 ± 2.2 |  |
| male | 4.8 ± 2.3 |  |
| diagnosis |  | 0.129 |
| acute recurrent tonsillitis | 5.7 ± 2.0 |  |
| peritonsillar abscess | 4.2 ± 2.1 |  |
| obstructive sleep apnea | 6.2 ± 2.1 |  |
| tonsil tumor | 6.0 ± 5.7 |  |
| ASA status |  | 0.371 |
| I | 4.9 ± 2.2 |  |
| II/ III | 5.5 ± 2.3 |  |
| honey |  | 0.843 |
| yes | 5.1 ± 2.1 |  |
| no | 5.1 ± 3.1 |  |
| fifth postoperative day |  |  |
| maximal pain | 4.8 ± 2.5 |  |
| age |  | 0.390 |
| <33.5 | 5.1 ± 2.3 |  |
| >33.5 | 4.6 ± 2.7 |  |
| gender |  | 0.081 |
| female | 5.6 ± 2.4 |  |
| male | 4.3 ± 2.4 |  |
| diagnosis |  | 0.133 |
| acute recurrent tonsillitis | 5.4 ± 2.4 |  |
| peritonsillar abscess | 3.8 ± 2.2 |  |
| obstructive sleep apnea | 6.2 ± 2.2 |  |
| tonsil tumor | 6.0 ± 5.7 |  |
| ASA status |  | 0.656 |
| I | 4.7 ± 2.5 |  |
| II/ III | 5.1 ± 2.6 |  |
| honey |  | 0.149 |
| yes | 4.6 ± 2.3 |  |
| no | 5.9 ± 3.3 |  |
