# Supplementary material for: The effect of adjuvant oral application of honey in the management of postoperative pain after tonsillectomy in adults: A pilot study
Source: PLoS One. 2020 Feb 10;15(2):e0228481. doi: 10.1371/journal.pone.0228481 (PMC7010464; doi:10.1371/journal.pone.0228481)
Supplement: S3 Table — (DOCX) [file pone.0228481.s004.docx]

**S3 table** Influence of demographic parameters on minimal pain

| first postoperative day | Mean ± SD | p-value |
| --- | --- | --- |
| minimal pain | 2.3 ± 1.7 |  |
| age |  | 0.322 |
| <33.5 | 2.5 ± 1.6 |  |
| >33.5 | 2.1 ± 1.8 |  |
| gender |  |  |
| female | 2.9 ± 1.6 | **0.021** |
| male | 1.9 ± 1.6 |  |
| diagnosis |  | 0.401 |
| acute recurrent tonsillitis | 2.7 ± 1.6 |  |
| peritonsillar abscess | 1.8 ± 1.5 |  |
| obstructive sleep apnea | 2.7 ± 2.7 |  |
| tonsil tumor | 2.0 |  |
| ASA status |  | 0.705 |
| I | 2.2 ± 1.5 |  |
| II/ III | 2.4 ± 1.9 |  |
| honey |  | **0.023** |
| yes | 2.2 ± 1.7 |  |
| no | 4.3 ± 0.6 |  |
| second postoperative day |  |  |
| minimal pain | 2.2 ± 1.6 |  |
| age |  | 0.217 |
| <33.5 | 2.4 ± 1.4 |  |
| >33.5 | 2.0 ± 1.8 |  |
| gender |  | **0.002** |
| female | 3.1 ± 1.8 |  |
| male | 1.6 ± 1.2 |  |
| diagnosis |  | 0.081 |
| acute recurrent tonsillitis | 2.6 ± 1.6 |  |
| peritonsillar abscess | 1.6 ± 1.3 |  |
| obstructive sleep apnea | 2.2 ± 1.5 |  |
| tonsil tumor | 4.5 ± 3.5 |  |
| ASA status |  | 0.522 |
| I | 2.0 ± 1.5 |  |
| II/ III | 2.4 ± 1.8 |  |
| honey |  | 0.978 |
| yes | 2.2 ± 1.6 |  |
| no | 2.2 ± 2.2 |  |
| third postoperative day |  |  |
| minimal pain | 2.2 ± 1.4 |  |
| age |  | 0.570 |
| <33.5 | 1.9 ± 1.3 |  |
| >33.5 | 1.8 ± 1.4 |  |
| gender |  | **0.003** |
| female | 2.5 ± 1.4 |  |
| male | 1.4 ± 1.1 |  |
| diagnosis |  | 0.394 |
| acute recurrent tonsillitis | 2.2 ± 1.5 |  |
| peritonsillar abscess | 1.5 ± 1.3 |  |
| obstructive sleep apnea | 2.0 ± 1.3 |  |
| tonsil tumor | 2.0 ± 1.4 |  |
| ASA status |  | 0.191 |
| I | 1.6 ± 1.3 |  |
| II/ III | 2.1 ± 1.4 |  |
| honey |  | 0.467 |
| yes | 1.8 ± 1.3 |  |
| no | 2.2 ± 1.7 |  |
| fourth postoperative day |  |  |
| minimal pain | 1.7 ± 1.4 |  |
| age |  | 0.591 |
| <33.5 | 1.7 ± 1.4 |  |
| >33.5 | 1.6 ± 1.5 |  |
| gender |  | **0.010** |
| female | 2.3 ± 1.6 |  |
| male | 1.2 ± 1.1 |  |
| diagnosis |  | 0.560 |
| acute recurrent tonsillitis | 1.9 ± 1.6 |  |
| peritonsillar abscess | 1.4 ± 1.4 |  |
| obstructive sleep apnea | 1.7 ± 1.0 |  |
| tonsil tumor | 2.0 ± 1.4 |  |
| ASA status |  | 0.209 |
| I | 1.4 ± 1.3 |  |
| II/ III | 1.9 ± 1.5 |  |
| honey |  | 0.276 |
| yes | 1.5 ± 1.3 |  |
| no | 2.2 ± 1.9 |  |
| fifth postoperative day |  |  |
| minimal pain | 1.5 ± 1.4 |  |
| age |  | 0.786 |
| <33.5 | 1.6 ± 1.4 |  |
| >33.5 | 1.5 ± 1.4 |  |
| gender |  | **0.004** |
| female | 2.3 ± 1.7 |  |
| male | 1.0 ± 0.8 |  |
| diagnosis |  | 0.541 |
| acute recurrent tonsillitis | 1.7 ± 1.5 |  |
| peritonsillar abscess | 1.2 ± 1.2 |  |
| obstructive sleep apnea | 1.3 ± 0.8 |  |
| tonsil tumor | 2.8 ± 2.5 |  |
| ASA status |  | 0.660 |
| I | 1.4 ± 1.3 |  |
| II/ III | 1.6 ± 1.4 |  |
| honey |  | **0.049** |
| yes | 1.3 ± 1.2 |  |
| no | 2.4 ± 1.8 |  |
