# Supplementary material for: The effect of adjuvant oral application of honey in the management of postoperative pain after tonsillectomy in adults: A pilot study
Source: PLoS One. 2020 Feb 10;15(2):e0228481. doi: 10.1371/journal.pone.0228481 (PMC7010464; doi:10.1371/journal.pone.0228481)
Supplement: S4 Table — (DOCX) [file pone.0228481.s005.docx]

**S4 table** Influence of process parameter on pain in activity

| first postoperative day | Mean ± SD | p-value |
| --- | --- | --- |
| pain in activity | 4.6 ± 2.3 |  |
| regular intake of pain killers |  | 0.111 |
| yes | 5.7 ± 2.3 |  |
| no | 4.4 ± 2.3 |  |
| predominant sedative |  | **0.034** |
| no | 7.0 ± 1.4 |  |
| midazolam | 4.6 ± 2.2 |  |
| clorazepate | 7.0 |  |
| intraoperative remifentanil |  | 0.470 |
| yes | 4.8 ± 2.3 |  |
| no | 4.3 ± 2.4 |  |
| opioids in recovery room |  | **0.002** |
| yes (piritramid) | 5.5 ± 2.2 |  |
| no | 3.7 ± 2.0 |  |
| opioids on ward |  | **0.007** |
| yes | 5.9 ± 2.4 |  |
| no | 4.0 ± 2.1 |  |
| predominant opioid on ward |  | **0.034** |
| no | 4.0 ± 2.1 |  |
| tramadol | 6.3 ± 1.7 |  |
| piritramid | 5.0 ± 4.4 |  |
| tapentadol | 5.5 ± 3.5 |  |
| tramadol |  | **0.006** |
| yes | 6.3 ± 1.7 |  |
| no | 4.2 ± 2.2 |  |
| piritramid |  | 0.610 |
| yes | 5.0 ± 4.4 |  |
| no | 4.6 ± 2.2 |  |
| tapentadol |  | 0.594 |
| yes | 5.5 ± 3.5 |  |
| no | 4.5 ± 2.3 |  |
| additional opioid on ward |  | 0.337 |
| yes | 7.00 |  |
| no | 4.5 ± 2.3 |  |
| predominant non-opioid on ward |  | 0.574 |
| no | 5.0 ± 2.8 |  |
| metamizole | 4.6 ± 2.3 |  |
| acetaminophen | 7.0 |  |
| additional non-opioid on ward |  | 0.438 |
| no | 4.6 ± 2.3 |  |
| ibuprofen | 3.0 |  |
| metamizole | 7.0 |  |
| preoperative pain therapy |  | 0.973 |
| yes | 4.7 ± 2.5 |  |
| no | 4.6 ± 2.3 |  |
| preoperative counseling on postoperative pain management |  | 0.365 |
| no | 3.2 ± 3.0 |  |
| yes, general | 4.7 ± 2.3 |  |
| yes, special | 5.0 ± 2.0 |  |
| second postoperative day |  |  |
| pain in activity | 5.0 ± 2.3 |  |
| regular intake of pain killers |  | 0.559 |
| yes | 5.2 ± 2.9 |  |
| no | 4.5 ± 2.2 |  |
| predominant sedative |  | **0.024** |
| no | 7.0 ± 1.4 |  |
| midazolam | 4.5 ± 2.1 |  |
| clorazepat | 8.0 |  |
| intraoperative remifentanil |  | 0.660 |
| yes | 4.8 ± 2.3 |  |
| no | 4.3 ± 2.3 |  |
| opioid in recovery room |  | **<0.001** |
| yes (piritramid) | 5.7 ± 2.0 |  |
| no | 3.4 ± 1.9 |  |
| opioid on ward |  | **0.002** |
| yes | 5.8 ± 2.0 |  |
| no | 3.7 ± 2.1 |  |
| predominant opioid on ward |  | **0.014** |
| no | 3.7 ± 2.1 |  |
| tramadol | 5.8 ± 2.2 |  |
| piritramid | 6.0 ± 1.0 |  |
| tapentadol | 6.0 |  |
| tramadol |  | **0.009** |
| yes | 5.8 ± 2.2 |  |
| no | 3.9 ± 2.1 |  |
| piritramid |  | 0.199 |
| yes | 6.0 ± 1.0 |  |
| no | 4.5 ± 2.3 |  |
| tapentadol |  | 0.582 |
| yes | 6.0 |  |
| no | 4.5 ± 2.3 |  |
| additional opioid on ward |  | 0.231 |
| yes | 6.5 ± 0.7 |  |
| no | 4.5 ± 2.3 |  |
| predominant non-opioid on ward |  | 0.420 |
| no | 0.0 |  |
| metamizol | 4.5 ± 2.3 |  |
| acetaminophen | 7.0 |  |
| etoricoxib | 6.0 |  |
| additional non-opioid on ward |  | 0.428 |
| no | 4.5 ± 2.2 |  |
| ibuprofen | 7.0 ± 4.2 |  |
| metamizole | 6.0 |  |
| preoperative pain therapy |  | 0.680 |
| yes | 4.7 ± 4.7 |  |
| no | 4.6 ± 2.1 |  |
| preoperative counseling on postoperative pain management |  | 0.828 |
| no | 4.6 ± 2.1 |  |
| yes, general | 4.5 ± 2.3 |  |
| yes, special | 5.0 ± 2.5 |  |
| third postoperative day |  |  |
| pain in activity | 4.1 ± 2.1 |  |
| regular intake of pain killers |  | 0.164 |
| yes | 4.9 ± 1.8 |  |
| no | 3.9 ± 2.1 |  |
| predominant sedative |  | 0.084 |
| no | 5.8 ± 1.7 |  |
| midazolam | 4.1 ± 2.0 |  |
| clorazepat | 5.0 |  |
| intraoperative remifentanil |  | 0.274 |
| yes | 4.3 ± 2.1 |  |
| no | 3.7 ± 2.1 |  |
| opioid in recovery room |  | **0.005** |
| yes (piritramid) | 4.8 ± 2.1 |  |
| no | 3.2 ± 1.7 |  |
| opioid on ward |  | 0.099 |
| yes | 4.9 ± 2.2 |  |
| no | 3.7 ± 2.0 |  |
| predominant opioid on ward |  | 0.271 |
| no | 3.7 ± 2.0 |  |
| tramadol | 4.9 ± 2.3 |  |
| codeine | 5 |  |
| tapentadol | 6.0 |  |
| predominant non-opioid on ward |  | 0.188 |
| no | 1.0 |  |
| metamizol | 4.0 ± 2.1 |  |
| acetaminophen | 6.0 |  |
| etoricoxib | 6.0 |  |
| additional non-opioid on ward |  | 0.691 |
| no | 4.0 ± 2.1 |  |
| ibuprofen | 5.0 |  |
| preoperative pain therapy |  | 0.610 |
| yes | 3.7 ± 3.7 |  |
| no | 4.1 ± 2.0 |  |
| preoperative counseling on postoperative pain management |  | 0.892 |
| no | 4.0 ± 1.6 |  |
| yes, general | 4.1 ± 2.1 |  |
| yes, special | 3.8 ± 2.3 |  |
| fourth postoperative day |  |  |
| pain in activity | 3.6 ± 2.1 |  |
| regular intake of pain killers |  | 0.536 |
| yes | 4.0 ± 2.2 |  |
| no | 3.5 ± 2.1 |  |
| predominant sedative |  | **0.037** |
| no | 5.5 ± 2.1 |  |
| midazolam | 3.7 ± 2.0 |  |
| clorazepat | 2.0 |  |
| intraoperative remifentanil |  | 0.547 |
| yes | 3.8 ± 2.1 |  |
| no | 3.4 ± 2.2 |  |
| opioid in recovery room |  | **0.017** |
| yes (piritramid) | 4.3 ± 2.1 |  |
| no | 2.9 ± 1.9 |  |
| opioid on ward |  | 0.117 |
| yes | 4.8 ± 2.1 |  |
| no | 3.4 ± 2.1 |  |
| predominant opioid on ward |  | 0.232 |
| no | 3.4 ± 2.1 |  |
| tramadol | 4.4 ± 2.1 |  |
| piritramid | 8.0 |  |
| tapentadol | 4.5 ± 0.7 |  |
| additional opioid on ward |  | 0.254 |
| yes | 3.5 ± 2.1 |  |
| no | 5.7 ± 2.1 |  |
| predominant non-opioid on ward |  | 0.204 |
| no | 2.3 ± 2.3 |  |
| metamizol | 3.7 ± 2.0 |  |
| acetaminophen | 6.0 |  |
| etoricoxib | 4.0 |  |
| additional non-opioid on ward |  | 0.873 |
| no | 3.6 ± 2.1 |  |
| ibuprofen | 3.0 |  |
| preoperative pain therapy |  | 0.585 |
| yes | 3.0 ± 2.7 |  |
| no | 3.7 ± 2.1 |  |
| preoperative counseling on postoperative pain management |  | 0.848 |
| no | 3.2 ± 0.8 |  |
| yes, general | 3.7 ± 2.1 |  |
| yes, special | 3.4 ± 2.4 |  |
| fifth postoperative day |  |  |
| pain in activity | 3.3 ± 2.2 |  |
| regular intake of pain killers |  | 0.552 |
| yes | 3.7 ± 2.2 |  |
| no | 3.3 ± 2.2 |  |
| predominant sedative |  | 0.092 |
| no | 4.0 ± 3.2 |  |
| midazolam | 3.3 ± 2.0 |  |
| clorazepat | 9.0 |  |
| intraoperative remifentanil |  | 0.563 |
| yes | 3.5 ± 2.3 |  |
| no | 3.0 ± 2.0 |  |
| opioid in recovery room |  | **0.023** |
| yes (piritramid) | 4.0 ± 2.2 |  |
| no | 2.6 ± 2.0 |  |
| opioid on ward |  | 0.331 |
| yes | 4.5 ± 2.3 |  |
| no | 3.2 ± 2.2 |  |
| predominant opioid on ward |  | 0.331 |
| no | 3.2 ± 2.2 |  |
| tramadol | 4.5 ± 2.3 |  |
| additional opioid on ward |  | 0.654 |
| yes | 3.4 ± 2.2 |  |
| no | - |  |
| predominant non-opioid on ward |  | 0.204 |
| no | 2.3 ± 2.3 |  |
| metamizol | 3.4 ± 0.1 |  |
| acetaminophen | 5.0 |  |
| etoricoxib | 0.0 |  |
| additional non-opioid on ward |  | 0.311 |
| no | 3.3 ± 2.2 |  |
| ibuprofen | 5.0 |  |
| preoperative pain therapy |  | 0.452 |
| yes | 2.8 ± 2.1 |  |
| no | 3.4 ± 2.1 |  |
| preoperative counseling on postoperative pain management |  | 0.346 |
| no | 4.0 ± 1.2 |  |
| yes, general | 3.1 ± 2.2 |  |
| yes, special | 4.0 ± 2.7 |  |
