# Supplementary material for: The effect of adjuvant oral application of honey in the management of postoperative pain after tonsillectomy in adults: A pilot study
Source: PLoS One. 2020 Feb 10;15(2):e0228481. doi: 10.1371/journal.pone.0228481 (PMC7010464; doi:10.1371/journal.pone.0228481)
Supplement: S5 Table — (DOCX) [file pone.0228481.s006.docx]

**S5 table** Influence of process parameter on maximal pain

| first postoperative day | Mean ± SD | p-value |
| --- | --- | --- |
| maximal pain | 5.8 ± 2.3 |  |
| regular intake of pain killers |  | 0.337 |
| yes | 6.4 ± 2.7 |  |
| no | 5.7 ± 2.2 |  |
| predominant sedative |  | 0.069 |
| no | 7.0 ± 1.4 |  |
| midazolam | 6.0 ± 2.2 |  |
| clorazepate | 8.0 |  |
| intraoperative remifentanil |  | **0.023** |
| yes | 6.4 ± 1.9 |  |
| no | 4.9 ± 2.5 |  |
| opioids in recovery room |  | **<0.001** |
| yes (piritramid) | 6.9 ± 1.9 |  |
| no | 4.6 ± 2.0 |  |
| opioids on ward |  | **0.002** |
| yes | 7.3 ± 1.6 |  |
| no | 5.1 ± 2.2 |  |
| predominant opioid on ward |  | **0.006** |
| no | 5.0 ± 2.1 |  |
| tramadol | 7.0 ± 1.9 |  |
| piritramid | 6.9 ± 1.6 |  |
| tapentadol | 7.0 ± 1.4 |  |
| tramadol |  | **0.045** |
| yes | 7.0 ± 1.9 |  |
| no | 5.5 ± 2.1 |  |
| piritramid |  | **0.027** |
| yes | 6.9 ± 1.6 |  |
| no | 5.5 ± 2.2 |  |
| tapentadol |  | 0.420 |
| yes | 7.0 ± 1.4 |  |
| no | 5.8 ± 2.2 |  |
| additional opioid on ward |  | 0.315 |
| yes | 8.00 |  |
| no | 5.7 ± 2.3 |  |
| predominant non-opioid on ward |  | 0.610 |
| no | 7.0 ± 1.4 |  |
| metamizole | 5.7 ± 2.3 |  |
| acetaminophen | 0.0 |  |
| additional non-opioid on ward |  | 0.851 |
| no | 5.8 ± 2.3 |  |
| ibuprofen | 6.0 |  |
| metamizole | 7.0 |  |
| preoperative pain therapy |  | 0.945 |
| yes | 5.7 ± 4.5 |  |
| no | 5.8 ± 2.1 |  |
| preoperative counseling on postoperative pain management |  | 0.446 |
| no | 6.6 ± 2.9 |  |
| yes, general | 5.9 ± 2.2 |  |
| yes, special | 5.1 ± 2.3 |  |
| second postoperative day |  |  |
| maximal pain | 5.7 ± 2.3 |  |
| regular intake of pain killers |  | 0.644 |
| yes | 6.1 ± 2.9 |  |
| no | 5.7 ± 2.2 |  |
| predominant sedative |  | 0.272 |
| no | 7.0 ± 1.4 |  |
| midazolam | 5.8 ± 2.3 |  |
| clorazepat | 8.0 |  |
| intraoperative remifentanil |  | 0.099 |
| yes | 6.2 ± 2.2 |  |
| no | 5.0 ± 2.4 |  |
| opioid in recovery room |  | **0.001** |
| yes (piritramid) | 6.8 ± 2.0 |  |
| no | 4.6 ± 2.1 |  |
| opioid on ward |  | **0.003** |
| yes | 7.0 ± 1.9 |  |
| no | 4.8 ± 2.2 |  |
| predominant opioid on ward |  | **0.014** |
| no | 4.8 ± 2.2 |  |
| tramadol | 6.9 ± 2.0 |  |
| piritramid | 8.0 |  |
| tapentadol | 6.0 |  |
| tramadol |  | **0.012** |
| yes | 6.9 ± 2.0 |  |
| no | 5.1 ± 2.3 |  |
| piritramid |  | 0.060 |
| yes | 8.0 |  |
| no | 5.6 ± 2.3 |  |
| tapentadol |  | 0.945 |
| yes | 6.0 |  |
| no | 5.7 ± 2.4 |  |
| additional opioid on ward |  | 0.252 |
| yes | 8.0 |  |
| no | 5.6 ± 2.3 |  |
| predominant non-opioid on ward |  | 0.864 |
| no | - |  |
| metamizol | 5.7 ± 2.4 |  |
| acetaminophen | 7.0 |  |
| etoricoxib | 6.0 |  |
| additional non-opioid on ward |  | 0.443 |
| no | 5.6 ± 2.3 |  |
| ibuprofen | 8.0 ± 2.8 |  |
| metamizole | 6.0 |  |
| preoperative pain therapy |  | 0.556 |
| yes | 5.0 ± 4.4 |  |
| no | 5.8 ± 2.2 |  |
| preoperative counseling on postoperative pain management |  | 0.644 |
| no | 6.5 ± 3.0 |  |
| yes, general | 5.8 ± 2.3 |  |
| yes, special | 5.2 ± 2.2 |  |
| third postoperative day |  |  |
| maximal pain | 5.4 ± 2.1 |  |
| regular intake of pain killers |  | 0.730 |
| yes | 5.2 ± 1.9 |  |
| no | 5.5 ± 2.2 |  |
| predominant sedative |  | 0.188 |
| no | 6.8 ± 1.5 |  |
| midazolam | 5.4 ± 2.1 |  |
| clorazepat | 8.0 |  |
| intraoperative remifentanil |  | 0.358 |
| yes | 5.6 ± 1.9 |  |
| no | 5.1 ± 2.5 |  |
| opioid in recovery room |  | **0.005** |
| yes (piritramid) | 6.2 ± 2.1 |  |
| no | 4.5 ± 1.8 |  |
| opioid on ward |  | **0.017** |
| yes | 6.7 ± 1.8 |  |
| no | 4.9 ± 2.1 |  |
| predominant opioid on ward |  | 0.072 |
| no | 4.9 ± 2.1 |  |
| tramadol | 6.6 ± 1.9 |  |
| codeine | 5.0 |  |
| tapentadol | 7.0 |  |
| tramadol |  | **0.021** |
| yes | 6.6 ± 1.9 |  |
| no | 5.0 ± 2.1 |  |
| piritramid |  | - |
| yes | - |  |
| no | 5.4 ± 2.1 |  |
| tapentadol |  | 0.593 |
| yes | 7.0 |  |
| no | 5.4 ± 2.1 |  |
| codeine |  | 0.259 |
| yes | 8.0 |  |
| no | 5.4 ± 2.1 |  |
| additional opioid on ward |  | - |
| yes | - |  |
| no | 2.1 ± 0.4 |  |
| predominant non-opioid on ward |  | 0.336 |
| no | 1.0 |  |
| metamizol | 5.5 ± 2.1 |  |
| acetaminophen | 6.0 |  |
| etoricoxib | 7.0 |  |
| additional non-opioid on ward |  | 0.873 |
| no | 5.4 ± 2.1 |  |
| ibuprofen | 6.0 |  |
| preoperative pain therapy |  | 0.121 |
| yes | 3.3 ± 2.5 |  |
| no | 5.5 ± 2.1 |  |
| preoperative counseling on postoperative pain management |  | 0.734 |
| no | 5.4 ± 2.1 |  |
| yes, general | 5.5 ± 2.1 |  |
| yes, special | 4.9 ± 2.3 |  |
| fourth postoperative day |  |  |
| maximal pain | 5.1 ± 2.3 |  |
| regular intake of pain killers |  | 0.614 |
| yes | 4.9 ± 2.8 |  |
| no | 5.2 ± 2.2 |  |
| predominant sedative |  | 0.155 |
| no | 6.8 ± 1.5 |  |
| midazolam | 5.0 ± 2.3 |  |
| clorazepat | 8.0 |  |
| intraoperative remifentanil |  | 0.512 |
| yes | 5.3 ± 2.2 |  |
| no | 4.9 ± 2.4 |  |
| opioid in recovery room |  | 0.059 |
| yes (piritramid) | 5.7 ± 2.3 |  |
| no | 4.5 ± 2.1 |  |
| opioid on ward |  | 0.066 |
| yes | 6.5 ± 2.1 |  |
| no | 4.9 ± 2.3 |  |
| predominant opioid on ward |  | 0.155 |
| no | 4.9 ± 2.3 |  |
| tramadol | 6.4 ± 2.2 |  |
| piritramid | 9.0 |  |
| tapentadol | 5.5 ± 0.7 |  |
| additional opioid on ward |  | 0.232 |
| yes | 5.1 ± 2.3 |  |
| no | 6.7 ± 2.1 |  |
| predominant non-opioid on ward |  | 0.150 |
| no | 3.2 ± 2.6 |  |
| metamizol | 5.4 ± 2.2 |  |
| acetaminophen | 6.0 |  |
| etoricoxib | 6.0 |  |
| additional non-opioid on ward |  | 1.000 |
| no | 5.1 ± 2.3 |  |
| ibuprofen | 5.0 |  |
| preoperative pain therapy |  | 0.402 |
| yes | 5.3 ± 4.0 |  |
| no | 5.2 ± 2.1 |  |
| preoperative counseling on postoperative pain management |  | 0.829 |
| no | 4.8 ± 1.9 |  |
| yes, general | 5.3 ± 2.3 |  |
| yes, special | 4.8 ± 2.7 |  |
| fifth postoperative day |  |  |
| maximal pain | 4.8 ± 2.5 |  |
| regular intake of pain killers |  | 0.651 |
| yes | 4.6 ± 2.7 |  |
| no | 4.9 ± 2.5 |  |
| predominant sedative |  | 0.130 |
| no | 6.5 ± 1.7 |  |
| midazolam | 4.7 ± 2.5 |  |
| clorazepat | 9.0 |  |
| intraoperative remifentanil |  | 0.353 |
| yes | 5.1 ± 2.4 |  |
| no | 4.4 ± 2.6 |  |
| opioid in recovery room |  | **0.038** |
| yes (piritramid) | 5.5 ± 2.5 |  |
| no | 4.0 ± 2.3 |  |
| opioid on ward |  | 0.549 |
| yes | 5.6 ± 2.6 |  |
| no | 4.8 ± 2.5 |  |
| predominant opioid on ward |  | 0.549 |
| no | 4.8 ± 2.5 |  |
| tramadol | 5.6 ± 2.6 |  |
| additional opioid on ward |  | - |
| yes | - |  |
| no | 4.9 ± 2.5 |  |
| predominant non-opioid on ward |  | 0.114 |
| no | 3.0 ± 2.6 |  |
| metamizol | 5.1 ± 2.4 |  |
| acetaminophen | 5.0 |  |
| additional non-opioid on ward |  | 0.591 |
| no | 4.8 ± 2.5 |  |
| ibuprofen | 6.0 |  |
| preoperative pain therapy |  | 0.384 |
| yes | 5.5 ± 3.7 |  |
| no | 4.9 ± 2.3 |  |
| preoperative counseling on postoperative pain management |  | 0.666 |
| no | 5.8 ± 2.1 |  |
| yes, general | 4.9 ± 2.4 |  |
| yes, special | 4.3 ± 3.0 |  |
