# Supplementary material for: The effect of adjuvant oral application of honey in the management of postoperative pain after tonsillectomy in adults: A pilot study
Source: PLoS One. 2020 Feb 10;15(2):e0228481. doi: 10.1371/journal.pone.0228481 (PMC7010464; doi:10.1371/journal.pone.0228481)
Supplement: S6 Table — (DOCX) [file pone.0228481.s007.docx]

**S6 table** Influence of process parameter on minimal pain

| first postoperative day | Mean ± SD | p-value |
| --- | --- | --- |
| minimal pain | 2.3 ± 1.7 |  |
| regular intake of pain killers |  | 0.174 |
| yes | 3.0 ± 1.7 |  |
| no | 2.2 ± 1.7 |  |
| predominant sedative |  | 0.054 |
| no | 4.0 ± 0.8 |  |
| midazolam | 2.3 ± 1.7 |  |
| clorazepate | 4.0 |  |
| intraoperative remifentanil |  | 0.087 |
| yes | 2.6 ± 1.6 |  |
| no | 1.8 ± 1.8 |  |
| opioids in recovery room |  | **0.048** |
| yes (piritramid) | 2.7 ± 1.6 |  |
| no | 1.9 ± 1.7 |  |
| opioids on ward |  | 0.340 |
| yes | 2.6 ± 2.0 |  |
| no | 2.1 ± 1.6 |  |
| predominant opioid on ward |  | 0.545 |
| no | 2.1 ± 1.6 |  |
| tramadol | 2.5 ± 2.1 |  |
| piritramid | 2.5 ± 2.4 |  |
| tapentadol | 3.5 ± 0.7 |  |
| additional opioid on ward |  | 0.460 |
| yes | 3.00 |  |
| no | 2.2 ± 1.7 |  |
| predominant non-opioid on ward |  | 0.440 |
| no | 3.5 ± 0.7 |  |
| metamizole | 2.2 ± 1.7 |  |
| acetaminophen | 3.0 |  |
| additional non-opioid on ward |  | 0.875 |
| no | 1.7 ± 0.3 |  |
| ibuprofen | 2.0 |  |
| metamizole | 3.0 |  |
| preoperative pain therapy |  | 0.837 |
| yes | 2.0 ± 1.0 |  |
| no | 2.3 ± 1.7 |  |
| preoperative counseling on postoperative pain management |  | 0.071 |
| no | 1.4 ± 1.0 |  |
| yes, general | 2.3 ± 1.7 |  |
| yes, special | 3.0 ± 1.3 |  |
| second postoperative day |  |  |
| minimal pain | 2.2 ± 1.6 |  |
| regular intake of pain killers |  | 0.163 |
| yes | 3.2 ± 2.3 |  |
| no | 2.0 ± 1.4 |  |
| predominant sedative |  | **0.040** |
| no | 3.5 ± 0.6 |  |
| midazolam | 2.2 ± 1.7 |  |
| clorazepat | 4.0 |  |
| intraoperative remifentanil |  | 0.136 |
| yes | 2.5 ± 1.6 |  |
| no | 1.8 ± 1.6 |  |
| opioid in recovery room |  | **0.022** |
| yes (piritramid) | 2.7 ± 1.8 |  |
| no | 1.7 ± 1.3 |  |
| opioid on ward |  | 0.087 |
| yes | 2.8 ± 1.9 |  |
| no | 1.7 ± 1.3 |  |
| predominant opioid on ward |  | 0.069 |
| no | 1.7 ± 1.3 |  |
| tramadol | 2.6 ± 2.0 |  |
| piritramid | 4.3 ± 0.6 |  |
| tapentadol | 2.0 |  |
| additional opioid on ward |  | 0.094 |
| yes | 4.5 ± 0.7 |  |
| no | 2.1 ± 1.6 |  |
| predominant non-opioid on ward |  | 0.786 |
| no |  |  |
| metamizol | 2.2 ± 1.7 |  |
| acetaminophen | 3.0 |  |
| etoricoxib | 2.0 |  |
| additional non-opioid on ward |  | 0.808 |
| no | 2.1 ± 1.5 |  |
| ibuprofen | 4.2 ± 4.0 |  |
| metamizole | 2.0 |  |
| preoperative pain therapy |  | 0.381 |
| yes | 3.7 ± 2.9 |  |
| no | 2.1 ± 1.5 |  |
| preoperative counseling on postoperative pain management |  | 0.226 |
| no | 2.2 ± 1.4 |  |
| yes, general | 2.2 ± 1.7 |  |
| yes, special | 2.6 ± 0.8 |  |
| third postoperative day |  |  |
| minimal pain | 2.2 ± 1.4 |  |
| regular intake of pain killers |  | 0.131 |
| yes | 2.6 ± 1.7 |  |
| no | 1.7 ± 1.2 |  |
| predominant sedative |  | **0.009** |
| no | 3.3 ± 0.5 |  |
| midazolam | 1.8 ± 1.3 |  |
| clorazepat | 3.0 |  |
| intraoperative remifentanil |  | 0.051 |
| yes | 2.1 ± 1.3 |  |
| no | 1.4 ± 1.3 |  |
| opioid in recovery room |  | 0.160 |
| yes (piritramid) | 2.1 ± 1.4 |  |
| no | 1.6 ± 1.3 |  |
| opioid on ward |  | 0.184 |
| yes | 2.5 ± 1.6 |  |
| no | 1.6 ± 1.2 |  |
| predominant opioid on ward |  | 0.251 |
| no | 1.6 ± 1.2 |  |
| tramadol | 2.3 ± 1.6 |  |
| codeine | 4.0 |  |
| tapentadol | 3.0 |  |
| additional opioid on ward |  | 0.909 |
| yes | 2.0 |  |
| no | 1.8 ± 1.4 |  |
| predominant non-opioid on ward |  | 0.134 |
| no | 0.0 |  |
| metamizol | 1.8 ± 1.3 |  |
| acetaminophen | 4.0 |  |
| etoricoxib | 3.0 |  |
| additional non-opioid on ward |  | 0.909 |
| no | 1.8 ± 1.4 |  |
| ibuprofen | 2.0 |  |
| preoperative pain therapy |  | 0.766 |
| yes | 2.0 ± 1.0 |  |
| no | 1.8 ± 1.4 |  |
| preoperative counseling on postoperative pain management |  | 0.104 |
| no | 1.0 ± 1.0 |  |
| yes, general | 1.8 ± 1.4 |  |
| yes, special | 2.4 ± 1.2 |  |
| fourth postoperative day |  |  |
| minimal pain | 1.7 ± 1.4 |  |
| regular intake of pain killers |  | 0.055 |
| yes | 2.4 ± 1.3 |  |
| no | 1.5 ± 1.4 |  |
| predominant sedative |  | **0.013** |
| no | 2.8 ± 0.5 |  |
| midazolam | 1.6 ± 1.4 |  |
| clorazepat | 3.0 |  |
| intraoperative remifentanil |  | 0.077 |
| yes | 1.8 ± 1.2 |  |
| no | 1.7 ± 1.4 |  |
| opioid in recovery room |  | 0.184 |
| yes (piritramid) | 1.9 ± 1.6 |  |
| no | 1.4 ± 1.2 |  |
| opioid on ward |  | 0.734 |
| yes | 2.0 ± 1.7 |  |
| no | 1.6 ± 1.4 |  |
| predominant opioid on ward |  | 0.500 |
| no | 1.6 ± 1.4 |  |
| tramadol | 1.5 ± 1.0 |  |
| piritramid | 6.0 |  |
| tapentadol | 1.5 ± 0.7 |  |
| additional opioid on ward |  | 0.493 |
| yes | 1.6 ± 1.3 |  |
| no | 3.0 ± 2.7 |  |
| predominant non-opioid on ward |  | 0.430 |
| no | 1.0 ± 0.6 |  |
| metamizol | 1.7 ± 1.5 |  |
| acetaminophen | 3.0 |  |
| etoricoxib | 1.0 |  |
| additional non-opioid on ward |  | 0.327 |
| no | 1.6 ± 1.4 |  |
| ibuprofen | 3.0 |  |
| preoperative pain therapy |  | 0.740 |
| yes | 1.5 ± 1.3 |  |
| no | 1.7 ± 1.4 |  |
| preoperative counseling on postoperative pain management |  | 0.225 |
| no | 1.0 ± 1.0 |  |
| yes, general | 1.6 ± 1.4 |  |
| yes, special | 2.3 ± 1.7 |  |
| fifth postoperative day |  |  |
| minimal pain | 1.5 ± 1.4 |  |
| regular intake of pain killers |  | 0.129 |
| yes | 2.1 ± 1.3 |  |
| no | 1.4 ± 1.4 |  |
| predominant sedative |  | 0.121 |
| no | 1.5 ± 0.6 |  |
| midazolam | 1.6 ± 1.4 |  |
| clorazepat | 2.0 |  |
| intraoperative remifentanil |  | 0.057 |
| yes | 1.7 ± 1.3 |  |
| no | 1.5 ± 1.2 |  |
| opioid in recovery room |  | 0.123 |
| yes (piritramid) | 1.8 ± 1.6 |  |
| no | 1.2 ± 1.0 |  |
| opioid on ward |  | 0.922 |
| yes | 2.2 ± 2.1 |  |
| no | 1.5 ± 1.2 |  |
| predominant opioid on ward |  | 0.917 |
| no | 1.4 ± 1.2 |  |
| tramadol | 2.2 ± 2.1 |  |
| additional opioid on ward |  | - |
| yes | - |  |
| no | 1.5 ± 1.4 |  |
| predominant non-opioid on ward |  | 0.690 |
| no | 1.0 ± 0.6 |  |
| metamizol | 1.6 ± 1.4 |  |
| acetaminophen | 1.0 |  |
| additional non-opioid on ward |  | 0.407 |
| no | 1.5 ± 1.4 |  |
| ibuprofen | 2.0 |  |
| preoperative pain therapy |  | 0.971 |
| yes | 2.4 ± 1.8 |  |
| no | 1.5 ± 1.3 |  |
| preoperative counseling on postoperative pain management |  | 0.310 |
| no | 1.5 ± 1.0 |  |
| yes, general | 1.4 ± 1.4 |  |
| yes, special | 2.0 ± 1.5 |  |
