# Supplementary material for: The effect of adjuvant oral application of honey in the management of postoperative pain after tonsillectomy in adults: A pilot study
Source: PLoS One. 2020 Feb 10;15(2):e0228481. doi: 10.1371/journal.pone.0228481 (PMC7010464; doi:10.1371/journal.pone.0228481)
Supplement: S1 File — (PDF) [file pone.0228481.s009.pdf]

Deutsches Register Klinischer Studien
German Clinical Trials Register

***PLEASE NOTE:*** *This trial has been registered retrospectively.*

## Trial Description

### Title

**QUIPS - Quality improvement in post operative pain treatment**

### Trial Acronym

**[---]***

### URL of the trial

**http://www.quips-projekt.de**

### Brief Summary in Lay Language

**QUIPS (quality improvement in postoperative pain management) is a multicentric, interdisciplinary benchmark project that aims at improving acute pain treatment in hospitals and outpatient clinics. The overall goal of this project is to improve treatment outcomes of postoperative pain therapy by collecting a standardized set of quality data, analyzing them and sending an immediate feedback to the hospitals. This webbased, automatic feedback system enables an internal and external benchmarking as well as an ongoing monitoring of processes.**

### Brief Summary in Scientific Language

**Postoperative pain is not only an unpleasant sensory experience but carries the risk of a number of potentially dangerous consequences. Severe pain can delay postoperative mobilisation and prolong rehabilitation. Postoperative pain increases endocrine stress reactions and has negative effects on wound healing. There is strong evidence that severe postoperative pain leads to chronic pain. For this reason, resolving pain according to state of the art standards should be a moral obligation for every healthcare provider.**

**Unfortunately, the quality outcome of postoperative pain therapy is far from being optimal in the clinical day-to-day business and is described: "as ineffective, inadequate and without the necessary organizational and scientific background" (Neugebauer 1998).**

**As pain therapy is not a particularly complex medical problem, the reason for this negative outcome is likely to be caused by the numerous non-medical obstacles that prevent an efficient implementation of pain therapy.**

**QUIPS's main objective is to improve postoperative pain therapy by collecting data on pain treatment quality outcomes, analyzing them and sending an immediate feedback to the participating hospitals. This system works independently from the hospitals' infrastructural conditions (i.e. IT or documentation systems) and guarantees an ongoing quality assurance by its internal and external benchmarking.**

## Organizational Data

- DRKS-ID: **DRKS00006153**
- Date of Registration in DRKS: **2014/05/12**
- Date of Registration in Partner Registry or other Primary Registry: **[---]***
- Investigator Sponsored/Initiated Trial (IST/IIT): **yes**
- Ethics Approval/Approval of the Ethics Committee: **Approved**
- (leading) Ethics Committee Nr.: **1184-08/03 , Ethikkommission der Friedrich-Schiller-Universität Jena an der Medizinischen Fakultät**

## Secondary IDs

## Health condition or Problem studied

- Free text: **Postoperative pain treatment**
- ICD10: **R52.0 - Acute pain**

## Interventions/Observational Groups

- Arm 1: **On postop day 1, patients are asked about their pain/their assessment of the pain therapy**

## Characteristics

- Study Type: **Non-interventional**
- Study Type Non-Interventional: **Observational study**
- Allocation: **Single arm study**
- Blinding: **[---]***
- Who is blinded: **[---]***
- Control: **Uncontrolled/Single arm**
- Purpose: **Supportive care**
- Assignment: **Single (group)**
- Phase: **N/A**
- Off-label use (Zulassungsüberschreitende Anwendung eines Arzneimittels): **N/A**

### Primary Outcome

**Outcome quality in postoperative pain therapy, assessed by the parameters pain intensity and side effects. Patient reported outcomes. Questionnaire filled in on postop day 1.**

### Secondary Outcome

**Patient satisfaction, process- and structure parameters of postoperative pain therapy (e.g. pain measurement, adequate medication, involving patients)**

## Countries of recruitment

- DE **Germany**
- AT **Austria**
- LU **Luxembourg**

## Locations of Recruitment

- University Medical Center **Klinik für Anästhesiologie und Intensivmedizin, Jena**

## Recruitment

- Planned/Actual: **Actual**
- (Anticipated or Actual) Date of First Enrollment: **2004/08/02**
- Target Sample Size: **1000000**
- Monocenter/Multicenter trial: **Multicenter trial**
- National/International: **International**

### Inclusion Criteria

- Gender: **Both, male and female**
- Minimum Age: **18 Years**
- Maximum Age: **no maximum age**

### Additional Inclusion Criteria

**- Patient consents to participate, - patient is post-op day 1, - patients speaks German, patient is able to fill in the questionnaire**

### Exclusion criteria

**- patient refuses participation**
**- patient doesn't speak German**
**- patient is not post-op day 1**
**- patient is not able to fill in the questionnaire (sedated, confused, fast asleep)**

## Addresses

- **Primary Sponsor**

**Deutsche Gesellschaft für Anästhesiologie und Intensivmedizin**
**Roritzerstraße 27**
**90419 Nürnberg**
**Germany**

Telephone: **[---]***
Fax: **[---]***
E-mail: **[---]***
URL: **www.dgai.de**

- **Contact for Scientific Queries**

**Universitätsklinikum JenaKlinik für Anänsthesiologie und Intensivmedizin**
**Mr. Prof. Winfried Meissner**
**Erlanger Allee 101**
**07747 Jena**
**Germany**

Telephone: **03641 9323353**
Fax: **03641 9323102**
E-mail: **winfried.meissner at med.uni-jena.de**
URL: **www.uniklinik-jena.de**

- **Contact for Public Queries**

**Universitätsklinik JenaKlinik für Anästhesiologie und Intensivmedizin**
**Ms. Claudia Weinmann**
**Erlanger Allee 101**
**07747 Jena**
**Germany**

Telephone: **03641 9323158**
Fax: **03641 9323102**
E-mail: **claudia.weinmann at med.uni-jena.de**
URL: **www.quips-projekt.de**

## Sources of Monetary or Material Support

- **Public funding institutions financed by tax money/Government funding body (German Research Foundation (DFG), Federal Ministry of Education and Research (BMBF), etc.)**

  **Bundesministerium für Bildung und Forschung Dienstsitz Berlin**
  **Friedrichstraße 130 B**
  **10117 Berlin**
  **Germany**

  Telephone: **[---]***
  Fax: **[---]***
  E-mail: **[---]***
  URL: **www.bmbf.de**
- **[---]***

  **nach BMBF Förderung: Projektfinanzierung durch Jahresgebühr, die von teilnehmenden Kliniken gezahlt wird**

  Telephone: **[---]***
  Fax: **[---]***
  E-mail: **[---]***
  URL: **[---]***

## Status

- Recruitment Status: **Recruiting ongoing**
- Study Closing (LPLV): **[---]***

## Trial Publications, Results and other documents

- Paper **QUIPS Publikationen**
- Abstract **AbstraktQuips**

** This entry means the parameter is not applicable or has not been set.*
**** This entry means that data is not displayed due to insufficient data privacy clearing.*
