# Supplementary material for: The effect of adjuvant oral application of honey in the management of postoperative pain after tonsillectomy in adults: A pilot study
Source: PLoS One. 2020 Feb 10;15(2):e0228481. doi: 10.1371/journal.pone.0228481 (PMC7010464; doi:10.1371/journal.pone.0228481)
Supplement: S2 File — (PDF) [file pone.0228481.s010.pdf]

QUIPS

Qualitätssicherung in der Postoperativen Schmerztherapie

Benutzerhandbuch Standard Operating Procedures (SOPs) Version 2018

Inhaltsverzeichnis

1 Projektüberblick 3
1.1 Hintergrund und Ziele des Projekts 3
1.2 Struktur von QUIPS (Qualitätssicherung in der postoperativen Schmerztherapie) 3
2 Organisatorischer Rahmen 3
2.1 Wer ist beteiligt? 4
2.1.1 Projektteam 4
2.1.2 Personal zur Datenerhebung 4
2.2 Vorbereitung 5
2.2.1 EDV, technische Voraussetzung 5
2.2.2 Teilnahme am Projekt 5
2.2.3 Personalschulung zur Datenerhebung 6
2.2.4 Ethikvotum 6
3 Erläuterungen des Fragebogens 7
3.1 Demographische Parameter 7
3.2 Ergebnisparameter 8
3.3 Prozessparameter 10
3.4 Freifelder 11
4. Datenerhebung 11
4.1 Randomisierung der Stationen 11
4.2 Ein- und Ausschlusskriterien 12
4.3 Datenerfassung 12
4.4 Langzeit- Follow-up/ Chronifizierung postoperativer Schmerzen 14
4.4.1 Datenerhebung für das Langzeit Follow up 14
4.5 Eingabe und Transfer der Daten zum zentralen Server 15
4.6 Häufige Fragen zur Datenerhebung 26
4.7 Sicherung der Datenqualität 30
5 Ergebnisrückmeldung 31
5.1 Charakterisierung des Feedbacks 31
5.2 Datenbestand 35
5.3 Outcome- Parameter Schmerzintensität 36
5.4 Outcome-Parameter Tätigkeiten/Stimmung 36
5.5 Station Prozessparameter 37
5.6 Rohdaten 39
6 Feedback-Meeting 40
6.1 Überblick Changemanagement 41
7. Zusammenfassung/Überblick 42
8 Zusätzliche Angebote aus dem PAIN OUT Projekt 43
8.1 Elektronische Wissensbibliothek (EKL) 43
8.2 Teilnahme an PAIN OUT (Improvement in postoperative PAIN OUTcome) 45
9. Zu guter Letzt ... 45

Copyright Nutzung durch Dritte vorbehaltlich Genehmigung durch DGAI/ DGCH Version 2018

1 Projektüberblick

1.1 Hintergrund und Ziele des Projekts

Postoperative Schmerzen sind nicht nur für die Patientinnen und Patienten eine unangenehme Erfahrung, sondern sie sind auch mit einer Reihe potentiell gefährlicher Konsequenzen verbunden. Starke Schmerzen können die postoperative Mobilisation verzögern und die Rehabilitationsdauer verlängern. Postoperative Schmerzen erhöhen die endokrine Stressreaktion und können negative Folgen für die Wundheilung haben. Es existiert eine Reihe von Hinweisen darauf, dass starke postoperative Beschwerden zur Entwicklung von chronischen Schmerzen führen können. Eine Behandlung akuter Schmerzen nach Kriterien, die dem derzeitigen Wissensstand entsprechen, sollte daher in jedem Fall eine ethische Selbstverständlichkeit sein.

Leider erreicht die Qualität der postoperativen Schmerztherapie im klinischen Alltag nicht immer einen optimalen Standard. Dies liegt offensichtlich nicht daran, dass die Schmerztherapie ein besonders komplexes medizinisches Problem darstellt, sondern dass ihrer Umsetzung eine Reihe von Widerständen gegenübersteht.

Ziel von QUIPS ist die Verbesserung der postoperativen Symptomkontrolle durch eine regelmäßige Erhebung von Daten zur Therapiequalität, ihrer Analyse und die zeitnahe Rückmeldung an die beteiligten Kliniken. Im Vordergrund stehen dabei Parameter der Ergebnisqualität aus der Patientenperspektive. Dadurch liefert QUIPS eine valide Datengrundlage zum inner- und interklinischen Vergleich, zur Identifikation von Defiziten, zum gezielten Ressourceneinsatz und zur Erfolgskontrolle von Verbesserungsbemühungen.

1.2 Struktur von QUIPS (Qualitätssicherung in der postoperativen Schmerztherapie)

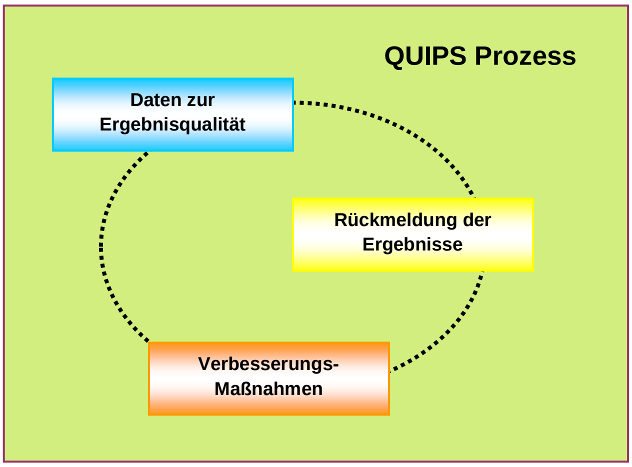

Eine Reihe von klinisch-demographischen Daten (z. B. Alter, OP-Art) sowie Parameter der Ergebnisqualität werden mit Hilfe eines standardisierten Patientenfragebogens erfasst. Nach der Eingabe in einen PC und der Versendung an den zentralen Server werden die Daten ausgewertet und die Ergebnisse an die teilnehmenden Kliniken online zurückgemeldet (siehe Abb.1).
Aufbauend auf einer anschließenden Defizitanalyse können Verbesserungsmaßnahmen eingeleitet und durch eine weitere Datenerhebung den Effekt der Maßnahmen direkt und zeitnah überprüfen. Alle Daten sind ab dem Zeitpunkt ihrer Übertragung auf den zentralen Server anonymisiert.

Abbildung 1: Schematischer Ablauf des Projektes

Auf der Grundlage der zurückgemeldeten Ergebnisse kann die Qualität der postoperativen Schmerztherapie:

- der eigenen Station im Zeitverlauf,
- der eigenen Station im Vergleich mit anderen Stationen
- nach Auswahl bestimmter Operationen und
- im Vergleich zu anderen teilnehmenden Zentren analysiert werden.
-

Es werden Ihnen die Outcome- Parameter sowie ausgewählte Prozessdaten in einer Maske (Benchmarkserver) aufbereitet und graphisch dargestellt.

2 Organisatorischer Rahmen

Die Präsidien der Deutschen Gesellschaft für Anästhesiologie und Intensivmedizin (DGAI) und des Berufsverbandes Deutscher Anästhesisten (BDA) haben in ihren Herbstsitzungen 2005 beschlossen, die Schirmherrschaft über dieses Projekt zu übernehmen und es ihren Mitgliedern zur Verfügung zu stellen.

Copyright Nutzung durch Dritte vorbehaltlich Genehmigung durch DGAI/ DGCH Version 2018

Die Deutsche Gesellschaft für Chirurgie (DGCH), sowie der Berufsverband Deutscher Chirurgen (BDC) und die Österreichische Gesellschaft für Anästhesiologie, Reanimation und Intensivmedizin (ÖGARI) sind Kooperationspartner des Projektes.

QUIPS kann in Zentren beliebiger Größe durchgeführt werden. Es ist nicht an eine bestimmte Bettenzahl oder ein besonderes OP-Spektrum gebunden. Die Hardwarevoraussetzungen sind gering. Es wird ein PC mit Internetanschluss benötigt. Eine Anbindung an Netzwerke oder Krankenhausinformationssysteme ist nicht notwendig.

2.1 Wer ist beteiligt?

Schmerztherapie ist immer eine interdisziplinäre Aufgabe. Dieses Projekt kann nur dann erfolgreich sein, wenn es ein Anliegen aller ist. Nehmen Sie vor Projektbeginn mit den beteiligten Berufsgruppen Kontakt auf. Dies betrifft das ärztliche und pflegerische Personal der beteiligten Kliniken bzw. Stationen ebenso wie die Physio- oder Ergotherapie. Es hat sich vielfach bewährt, eine klinikinterne Arbeitsgruppe „Akutschmerz“ aufzubauen, die auf der Grundlage der „QUIPS“-Daten konkrete Verbesserungsprojekte initiiert.
Auf administrativer Seite sollte mit der Geschäftsleitung, der Pflegedienstleitung und mit Mitarbeitern bestehender Qualitätsmanagementprogramme Kontakt aufgenommen werden. Kontaktieren Sie in Ihrem Klinikum die Ethikkommission, den Datenschutz und in manchen Zentren kann es sinnvoll sein, den Personalrat zu informieren, um Missverständnissen vorzubeugen, die bei jeder Art von inner- oder außerklinischen Vergleichen sehr schnell entstehen können.

2.1.1 Projektteam

Das Projekt richtet sich an Mitarbeiter aller Fachrichtungen, die ein Interesse an der Qualität der Patientenversorgung und -zufriedenheit haben und es sich zum Ziel gesetzt haben, die Ergebnisqualität der postoperativen Schmerztherapie zu verbessern.
Das Team sollte bestehen aus einer Projektleiterin/einem Projektleiter, einer Person, die die Aufgabe der Datenerfassung übernimmt, und den Kooperationspartnern in den Stationsführenden Kliniken, ohne deren Zusammenarbeit dieses Projekt nicht sinnvoll durchführbar ist, siehe dazu Abb. 2.

Hinweis:

Das Projektteam sollte über eine gewisse Kompetenz in der postoperativen Schmerztherapie verfügen. Ein Projektmitarbeiter sollte in die Erstellung von klinikinternen Standards zur postoperativen Schmerztherapie und deren Umsetzung im Klinikalltag integriert sein.

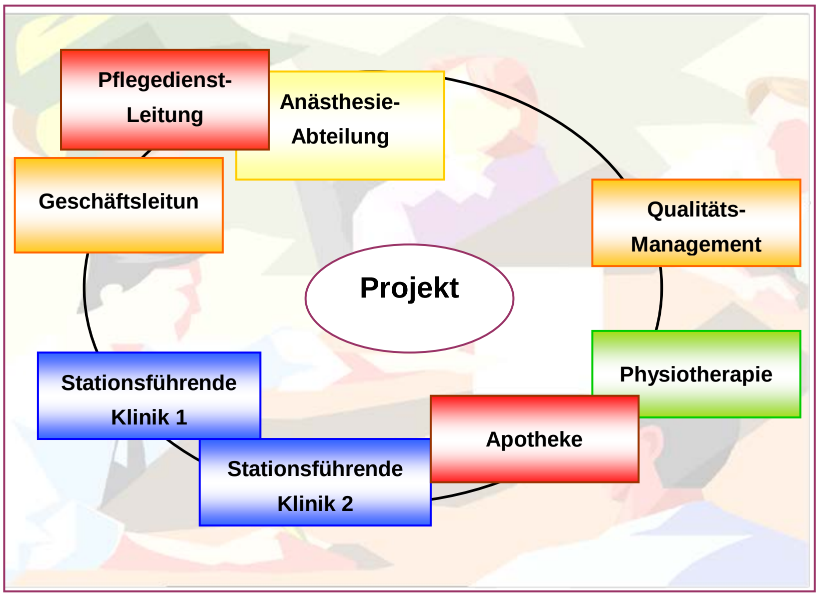

Abbildung 2: Ein Kooperationsmodell für QUIPS

2.1.2 Personal zur Datenerhebung

Zur Erfassung der Daten ist mindestens eine eingewiesene Person erforderlich. Grundsätzlich empfehlen wir, dass die Datenerhebung von einer Person durchgeführt werden soll, die **nicht** in das Pflegeteam der Station, auf der sich der Patient befindet, integriert ist. Im optimalen Falle ist dies eine Pflegekraft z. B. aus der Schmerzambulanz oder einer vergleichbaren Einrichtung. Auf die Einweisung ist großer Wert zu legen, da nur so eine objektive und zuverlässige Datenerfassung mit möglichst wenigen Fehlerquellen gewährleistet ist. Die Datenerhebung der Ergebnisparameter pro Patient/in dauert ca. 10 Minuten. Wenn pro Station 5 Patienten an vier Tagen in der Woche befragt würden, wäre dies ein Zeitaufwand von ca. 50 Minuten täglich, um innerhalb eines Quartals 240 Datensätze zu erhalten. Die zusätzliche Erhebung der Prozessparameter, inklusive Dateneingabe, nimmt pro Patient ca.10 min. mehr Zeit in Anspruch.

Copyright Nutzung durch Dritte vorbehaltlich Genehmigung durch DGAI/ DGCH Version 2018

Bei weniger als 20 Datensätzen/Patienten pro Quartal lassen sich keine sinnvollen Aussagen hinsichtlich der Ergebnisqualität treffen. Um eine sinnvolle Auswertung und Rückmeldung der Ergebnisse zu gewährleisten, sollten mindestens 30, besser 50 Patienten pro Einheit (z. B. pro Station) pro Quartal befragt werden.

2.2 Vorbereitung

2.2.1 EDV, technische Voraussetzung

Sie benötigen einen konventionellen PC mit Internetzugang. Zur Abklärung der technischen Anforderungen wenden Sie sich bitte an Ihre EDV-Abteilung, die alle weiteren Fragen mit unserem Kooperationspartner Firma TAKWA abklären kann.
Vor dem Beginn der Datenerhebung muss unbedingt die Stationskodierung der jeweils teilnehmenden Stationen der Klinik mit der Firma TAKWA anhand des Strukturfragebogens abgestimmt werden, um so die exakte Datenanalyse und das webbasierte Feedback pro Station und Klinik über den Benchmarkserver zu gewährleisten. Nähere Informationen zur Anwendung der webbasierten Eingabemaske erhalten Sie bei unserer Personalschulung.

2.2.2 Teilnahme am Projekt

Die Anmeldung zur Teilnahme am Projekt QUIPS erfolgt über die QUIPS- Website (www.quips-projekt.de), hier finden Sie auch die aktuellen Termine der angebotenen QUIPS- Schulungen und weiterer QUIPS-Veranstaltungen (Anwendertreffen, Statistik- Workshop).
Die Anmeldung zur QUIPS- Schulung erfolgt ebenfalls über unsere Homepage, bitte mit der Angabe des gewünschten Termins und der Anzahl der Teilnehmer (max. 3 Personen pro Klinik). Mit der Terminbestätigung erhalten die teilnehmenden Zentren die aktuelle Version des QUIPS-Fragebogens und das Handbuch zum Projekt QUIPS (SOPs). Nach der Absolvierung der Schulung erhalten die Teilnehmer die Vertragsunterlagen, ein Quiz und eine Teilnahmebestätigung. Das weitere Prozedere zur Teilnahme am Projekt QUIPS sieht vor, dass die unterschriebenen Verträge in doppelter Ausführung von Ihnen an die Geschäftsstelle der DGAI/BDA gesendet werden. Ein Exemplar verbleibt bei der Geschäftsleitung der DGAI/BDA und der zweite Vertrag wird unterschrieben an Sie zurückgesandt. Im Anschluss daran werden das QUIPS-Team und die kooperierende Firma TAKWA (siehe Abb. 3) durch die Geschäftsstelle der DGAI/ BDA über den erfolgten Vertragsabschluss informiert.

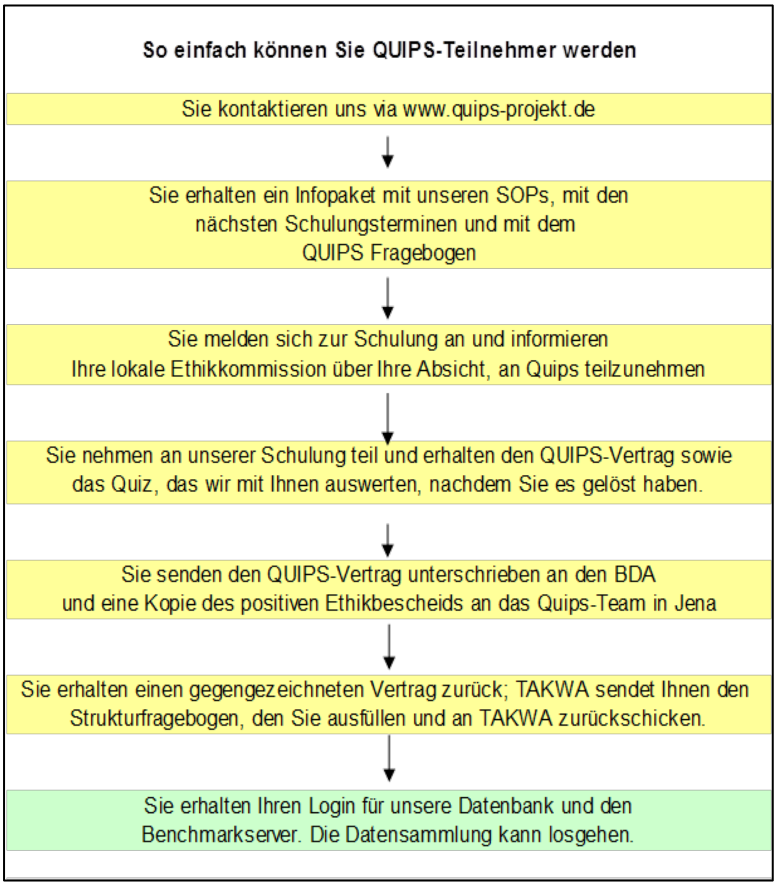

Abbildung 3: Prozedere Vertragsabschluss QUIPS

Die Passwortvergabe und der erforderliche Support für die webbasierte Dateneingabe und Ergebnisrückmeldung erfolgt nach Vertragsabschluss durch die kooperierende Softwarefirma TAKWA.
Mit der Teilnahme am Projekt QUIPS ist das einmalige Ausfüllen eines allgemeinen Strukturfragebogens zum jeweiligen operativen Zentrum verbunden. Sollten mehrere Stationen an dem Projekt teilnehmen oder neue Stationen später hinzukommen, ist für jede Station ein Strukturfragebogen (Station) auszufüllen. Die Zuordnung der Stationen zur jeweiligen chirurgischen Fachabteilung und die daraus folgende Kodierung werden mit der Firma TAKWA abgeglichen.

Copyright Nutzung durch Dritte vorbehaltlich Genehmigung durch DGAI/ DGCH Version 2018

2.2.3 Personalschulung zur Datenerhebung

Nach der Anmeldung zur Schulung erhalten die teilnehmenden Kliniken eine Bestätigung und die Tagesordnung (Abb.4) mit dem zeitlichen Rahmen in Form eines Flyers. Die vorherige Kenntnis der SOPs stellt eine optimale Vorbereitung für eine effektive Einweisung dar. Die Schulung führen wir zum einen im Rahmen der Kongresse (Deutscher Anästhesiecongress und Hauptstadtkongress der DGAI für Anästhesiologie und Intensivtherapie) sowie am Universitätsklinikum Jena durch.

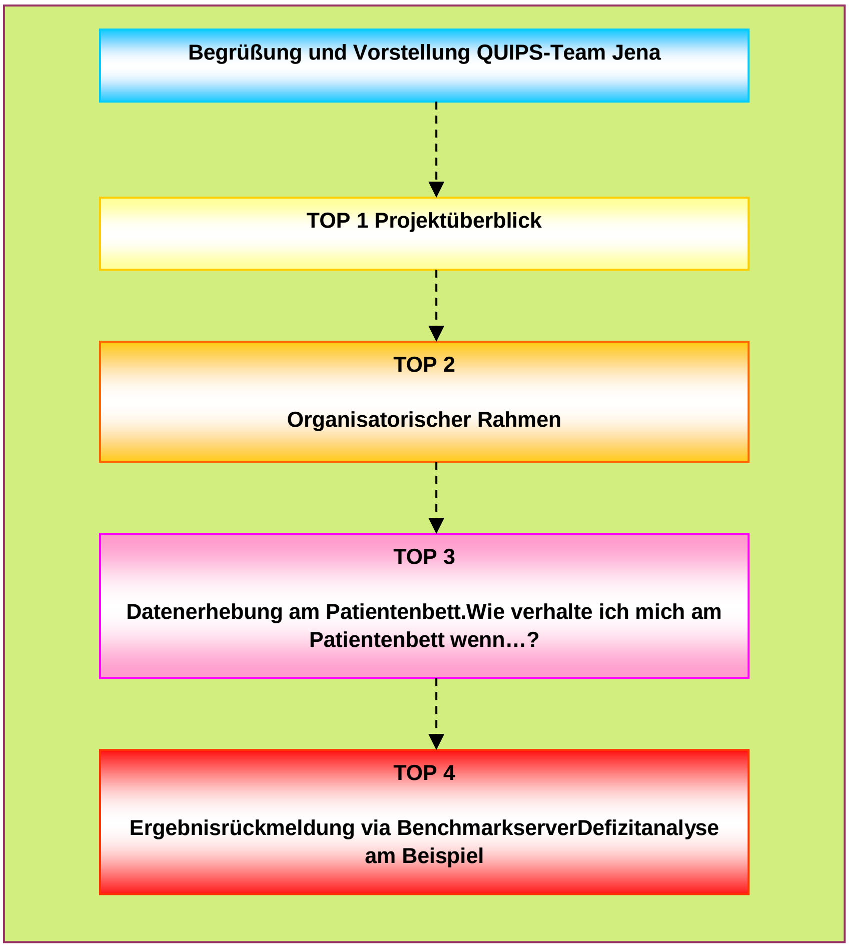

Die QUIPS-Schulung bietet die Möglichkeit bereits bestehende Fragen und Probleme zu diskutieren, um damit den zeitnahen Start der Datenerhebung zu gewährleisten. Es hat sich sehr bewährt, auch die Personen, z.B. Pflegepersonal o. a. im Workshop zu schulen, die später die Befragung durchführen werden.
Für jede teilnehmende Klinik stehen bis zu drei Schulungsplätze zu Verfügung. Die Kosten für die Teilnahme an der Schulung sind in der Teilnehmergebühr des Projektes QUIPS enthalten.
Die Tagesordnung (siehe Abb. 4) enthält eine allgemeine Einführung in das Projekt QUIPS und es werden die Inhalte und Prozesse der Datenerhebung ausführlich dargestellt.

Abbildung 4: Tagesordnung QUIPS-Schulung

2.2.4 Ethikvotum

Holen Sie sich bei Ihrer lokalen Ethikkommission eine Stellungnahme für die Durchführung des Projektes ein. Zur Einreichung benötigen Sie unter anderem den QUIPS-Fragebogen und die Einverständniserklärung für den Patienten.
Wir unterscheiden die Patienteninformation je nach Befragungsart: Die Standardvariante ist für die QUIPS Befragung, die einmalig nach der Operation während eines Krankenhausaufenthaltes (in Kap.3.2 beschrieben) stattfindet.
Für die Erfassung der Entstehung von chronischen Schmerzen können Sie Ihre Patienten zusätzlich nach 6 und 12 Monaten befragen. Dazu gibt es eine weitere Patienteninformation und Einverständniserklärung (in Kap.4.4.1 beschrieben).

Copyright Nutzung durch Dritte vorbehaltlich Genehmigung durch DGAI/ DGCH Version 2018

3 Erläuterung des Fragebogens

Im Folgenden wird der für das Projekt QUIPS verwendete Fragebogen Version 3.4 (Stand 2018) erläutert. Der Fragebogen ist in vier Bereiche gegliedert:

1. **Demographie**
2. **Ergebnisparameter** (Patientenfragebogen)

Und fakultativ:

3. **Prozessparameter** (Narkose, Prämedikation, Aufwachraum, Station)
4. **Freifelder** (für eigene Fragestellungen)

Kernpunkt des Projektes und obligatorischer Bestandteil sind die ersten beiden Teile des Fragebogens. Das Feedback der Ergebnisparameter ist Bestandteil des Basismoduls. Ihre Erhebung benötigt ca.10 Minuten pro Patient. Fakultativ können zusätzlich Prozessparameter erhoben werden, die die Art der Narkose, der Schmerztherapie und weitere Prozesse näher charakterisieren. Eine Auswahl der Prozessparameter ist auf dem den Benchmarkserver abgebildet. Für eine differenzierte Defizitanalase benötigt man jedoch die kompletten Rohdaten, abrufbar über einen Button auf dem Benchmarkserver. Die Freifelder erlauben zusätzlich die Erhebung individuell interessierender Fragen.

3.1 Demographische Parameter

Die demographischen Parameter werden vom Befragungspersonal ausgefüllt, wenn die Auswahl der zu befragenden Patienten feststeht. Die Daten enthalten die Zuordnung zur Station und zum jeweiligen Fachbereich. Persönliche Daten des Patienten, wie zum Beispiel Geschlecht, Alter, ASA-Status und durchgeführte Operation (OPS-Kodierung) werden erfasst.

Im Folgenden sind Originalzitate aus dem Fragebogen grün markiert.

D1 Nummer: Aus Gründen des Datenschutzes wird hier die 10-stellige Nummer eingetragen, die bei der webbasierten Eingabe des Datensatzes generiert wird. Um eine klinikinterne Zuordnung der Daten, zum Beispiel für die Vervollständigung eines Datensatzes zu einem späteren Zeitpunkt, zu ermöglichen, kann die generierte Nummer auf dem Fragebogen genutzt werden.

D6 Station: Die Bezeichnung der Station, auf welcher der Patient zum Befragungszeitpunkt liegt, ist entsprechend der vereinbarten Kodierung (Strukturfragebogen, siehe auch 2.2.1) vorzunehmen. Liegt ein Patient auf einer so genannten gemischten Station mit unterschiedlichen OP-Fachbereichen (z.B. Traumatologie und Kieferchirurgie), so sind für diese Station zwei Kodierungen (z.B. Station 2a und 2b) zu vergeben, damit der Patient eindeutig einer Fachrichtung zugeordnet werden kann.

D2 Geschlecht: Hier ist anzugeben, ob der Patient männlich oder weiblich ist.

D3 Alter: Aus Datenschutzgründen wird das Alter in Kategorien angegeben. Es ist jeweils die Kategorie anzukreuzen, in welche der Patient einzuordnen ist.
Beispiel: für einen Patienten von 55 Jahren wird die Kategorie "51-60 Jahre“ ausgewählt.

D4 Kalenderwoche und **Jahr der OP:** Die Kalenderwoche und das Jahr der OP werden entsprechend dem Eingabezeitpunkt automatisch vorgegeben und sind bei nicht übereinstimmenden Daten zu korrigieren.

D5 Post-OP-Tag: Hier ist einzutragen, an welchem Tag nach der OP die Befragung stattfindet, empfohlen wird der 1. postoperative Tag.

D7 OP-Dauer: Es sind Start und Ende der OP als Schnitt- und Naht-Zeit einzutragen, zum Beispiel OP-Schnitt: 12:45 Uhr und OP-Naht: 13:30 Uhr. Bei OP-Zeiten mit Datumswechsel, ist die Option [OP über 2 Tage] auszuwählen, damit die OP-Dauer richtig berechnet werden kann.

D8 ASA- Status: Hier ist die jeweils zutreffende ASA- Klassifikation des Patienten einzutragen.

D9 OPS: Es werden der Operationen- und Prozedurenschlüssel (OPS) eingetragen. Maximal 5 Nennungen sind möglich. Generell sollte der wichtigste Eingriff an erster Stelle genannt werden.

Copyright Nutzung durch Dritte vorbehaltlich Genehmigung durch DGAI/ DGCH Version 2018

Sollten mehr als 5 OPS in den Unterlagen aufgeführt sein, sollten die größten Eingriffe in der Reihenfolge ihrer Wichtigkeit eingetragen werden.
Um sich auch außerhalb Deutschlands anhand der durchgeführten Operationen zu vergleichen, nutzen Sie folgenden Link www.dimdi.de und übersetzen sich die Haupteingriffe.

D10 Einschluss des Patienten nicht möglich aus folgendem Grund: Sollte ein für die Stichprobe ausgewählter Patient nicht in die Befragung aufgenommen werden, so ist der Grund hierfür an dieser Stelle zu dokumentieren. Trifft einer der unter D10 genannten Gründe zu, so ist die Datenerhebung für diesen Patienten an dieser Stelle beendet. Sollte die Befragung des Patienten nicht möglich sein, weil der Patient sediert ist, sind die Prozessparameter zu erheben, um mögliche Nebenwirkungen der postoperativen Analgetika Therapie zu erfassen.
Auch die Dokumentation bzw. Eingabe der demographischen Daten von nicht befragten Patienten ist von größter Wichtigkeit, um systematische Verzerrungen der befragten Stichprobe und somit der Ergebnisqualität zu erfassen.

D11 Hat der Patient vor seinem Krankenhausaufenthalt Opioide gegen chronische Schmerzen genommen? Diese Frage ist mit ja, nein oder nicht erhebbar zu beantworten. Diese Angaben sind aus der Krankenakte zu entnehmen.

3.2 Ergebnisparameter

Nach der persönlichen Vorstellung sowie der kurzen Erläuterung des Projektes kann der Patient die Befragung ablehnen oder ihr zustimmen. Die Zustimmung erfolgt schriftlich, der Patient erhält eine Kopie der Einwilligung, das Original verbleibt beim Team der QUIPS-Befragung. Hinweis: Verwenden Sie zusätzlich das Logo/Namen Ihrer Klinik auf der Einwilligungserklärung.

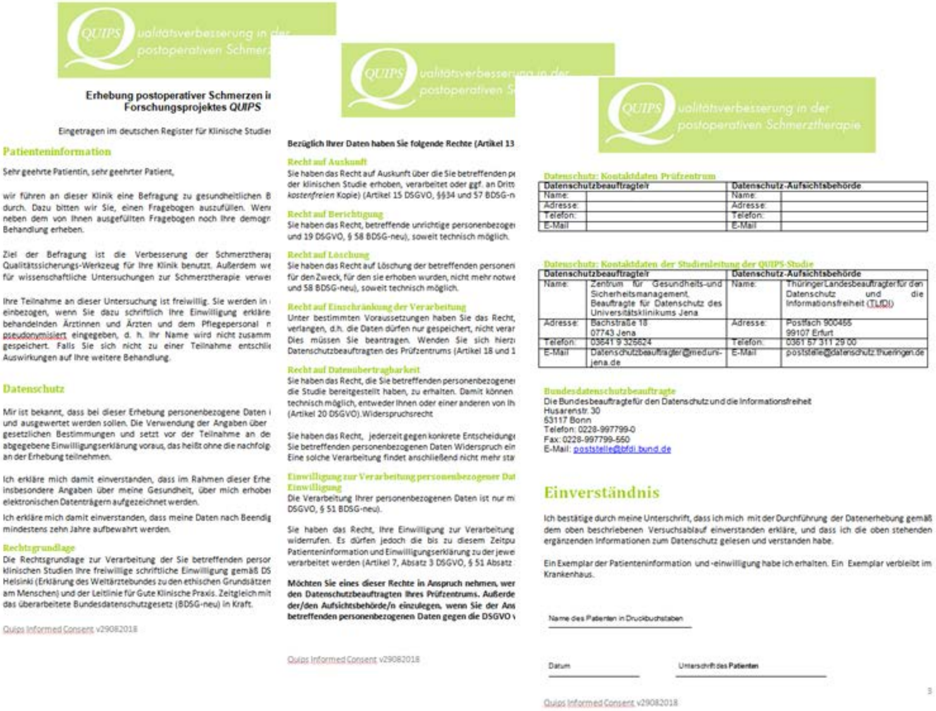

Abbildung 5: Patienteninformation und Einwilligungserklärung

Im Anschluss wird dem Patient der Ergebnis-Fragebogen (siehe auch Kapitel 4.3 Datenerfassung) ausgehändigt. Zusätzliche Ausführungen durch den Befragenden stellen eine Abweichung von der Standardisierung der Befragungssituation dar und sollten nur dann gegeben werden, wenn der Patient konkrete Fragen hat.
In manchen Situationen wird es nötig sein, dass der/die Befragende den Bogen im Auftrag des Patienten ausfüllt (z. B. der Patient hat seine Brille vergessen, hat die Schreibhand im Gipsverband etc.).
In diesem Fall sollte der Befragende die Fragen so neutral als möglich vorlesen und keinesfalls Einfluss auf die Antwort des Patienten nehmen. Generell sollte die Anzahl der auf diese Weise durchgeführten

Copyright Nutzung durch Dritte vorbehaltlich Genehmigung durch DGAI/ DGCH Version 2018

Befragungen so gering wie möglich gehalten werden, da die so gewonnenen Daten verzerrende Antworttendenzen in Richtung sozialer Erwünschtheit aufweisen.

Die ersten drei Fragen des Ergebnis-Fragebogens beziehen sich auf die Schmerzintensität bei Belastung, sowie stärkste und geringste Schmerzen seit der Operation. Die Fragen sind mit einer 11-stufigen numerischen Rating-Skala unterlegt, wobei 0 gleichbedeutend ist mit keinem Schmerz und 10 den stärksten vorstellbaren Schmerz bezeichnet. ***Hinweis:*** Es ist bei nicht eindeutigen Zuordnungen immer die „schlechtere" Antwort zu dokumentieren.
Bei Nachfragen kann dem Patienten erklärt werden,…

E 1 Schmerz bei Belastung
…dass hiermit jede Art von Belastung seit der OP gemeint ist, also z.B. sich aufsetzen, husten, waschen, umdrehen, etc. Die Verwendung der Skala von 0 bis 10 bezieht sich immer auf die Einschätzung des jeweiligen Patienten, 10 wäre dann also der stärkste Schmerz, den der Patient sich vorstellen kann.

E 2 Stärkster Schmerz seit der Operation
…dass der stärkste Schmerz durchaus über dem Belastungsschmerz liegen kann, z. B. der Schmerz direkt nach Aufwachen aus der Narkose. Es könnte auch sein, dass der Patient sich nicht belastet hat, weil er Schmerzen vermeiden will.

E 3 Geringster Schmerz seit der Operation
…wie stark seine Schmerzen in Ruhelage sind.

Die Fragen E4 bis E7 beziehen sich darauf, ob bestimmte Tätigkeiten oder die Stimmung durch den Schmerz beeinträchtigt sind. Mit „beeinträchtigt" ist gemeint: die Tätigkeit ist unmöglich oder nur unter großer Mühe möglich. Hier hat der Patient „Ja" oder „Nein" als Antwort zur Auswahl. ***Hinweis:*** Sollte eine Antwort fehlen, wird „nicht erhebbar" eingegeben.

Sind Sie durch Schmerzen beeinträchtigt:

E 4 bei **Bewegung**

E 5 beim **Husten oder tiefen Luftholen**

E 6 beim **Schlafen**

E 7 in Ihrer **Stimmung**

Die Fragen E8 bis E10 befassen sich mit weiteren Qualitätsmerkmalen der Schmerztherapie. Hier hat der Patient „Ja" oder „Nein" als Antwort zur Auswahl. ***Hinweis:*** Sollte eine Antwort fehlen, wird „nicht erhebbar" eingegeben.

E 8 Haben Sie sich seit der Operation sehr **müde** gefühlt?

E 9 Haben Sie seit der Operation unter **Übelkeit** gelitten?

E10 Haben Sie seit der Operation unter **Schwindel** gelitten?

Die nächsten Fragen des Ergebnis-Fragebogens betreffen die Qualität der präoperativen Aufklärung über die postoperative Schmerztherapie und deren Umsetzung aus Sicht des Patienten. ***Hinweis:*** Es ist bei nicht eindeutigen Zuordnungen immer die „schlechtere" Antwort zu dokumentieren. Sollte eine Antwort fehlen, wird „nicht erhebbar" eingegeben.

E11 Wurden Sie über die verschiedenen **Möglichkeiten Ihrer Schmerztherapie** informiert?
Die Fragen sind mit einer 11-stufigen numerischen Rating-Skala unterlegt, wobei 0 gleichbedeutend ist mit keinem Schmerz und 10 den stärksten vorstellbaren Schmerz bezeichnet.

E12 Wurden Sie in dem von Ihnen gewünschten Maß an **Entscheidungen** zu Ihrer Schmerztherapie beteiligt?
Die Antwort ist mit einer 11-stufigen numerischen Rating-Skala unterlegt, wobei 0 gleichbedeutend ist mit gar nicht in den Entscheidungsprozess einbezogen und 10 völlig ausreichende Einbeziehung in den Entscheidungsprozess bezeichnet.

Copyright Nutzung durch Dritte vorbehaltlich Genehmigung durch DGAI/ DGCH Version 2018

E13 Hätten Sie sich **MEHR Schmerztherapie** gewünscht, als Sie erhalten haben?
Hier hat der Patient „Ja“ oder „Nein“ als Antwort zur Auswahl.

E14 Bitte kreuzen Sie an, wie **zufrieden** Sie mit dem Ergebnis Ihrer **Schmerztherapie** seit Ihrer Operation sind:
Die Antwort ist mit einer 11-stufigen numerischen Rating-Skala unterlegt, wobei 0 gleichbedeutend ist mit völlig unzufrieden und 10 sehr zufrieden mit dem Ergebnis der Schmerztherapie.

E15 Haben Sie **nicht-medikamentöse** Methoden zur **Schmerzlinderung** benutzt oder erhalten?
Hier hat der Patient „Ja“ oder „Nein“ als Antwort zur Auswahl. Falls ja, sollte der Patient aus dem folgenden Menü auswählen:

Kühlkompressen
Akupunktur
Ablenkung (wie z. B. fernsehen, Musik hören, lesen)
Gespräche mit medizinischem Personal
Gespräche mit Freunden oder Verwandten
TENS (Transkutane elektrische Nervenstimulation)
Wärme
Meditation
Tiefes Atmen
Beten
Umhergehen
Massage
Vorstellungsbilder
Entspannung

Die letzte Frage eruiert chronische Schmerzen und deren Intensität im Hinblick auf die mögliche Beeinflussung der postoperativen Schmerzsymptomatik.

E16 Hatten Sie **ständige Schmerzen**, die **drei Monate oder länger** andauerten, bevor Sie wegen dieser Operation ins Krankenhaus gekommen sind?
Hier hat der Patient „Ja“ oder „Nein“ als Antwort zur Auswahl. Antwortet der Patient mit ja, sollte er die Frage **E16 a** und **E16 b** ausfüllen.

E16 a Falls ja, wie stark waren diese Schmerzen meistens?
Die Frage ist mit einer 11-stufigen numerischen Rating-Skala unterlegt, wobei 0 gleichbedeutend ist mit keinem Schmerz und 10 den stärksten vorstellbaren Schmerz bezeichnet.

E16 b Falls ja, wo traten diese ständigen Schmerzen auf?
Hier kann folgende Auswahl getroffen werden:

an der Körperstelle, die operiert wurde
an einer anderen Körperstelle
beides (Operationsstelle und woanders)

Am Ende des Ergebnisbogens ist vom Befragenden zu dokumentieren, ob ein Interview zur Beantwortung des Fragebogens geführt hat.

3.3 Prozessparameter

Die zu erhebenden Prozessdaten beziehen sich auf Details der Narkose, der Prämedikation und der postoperativen Schmerztherapie im Aufwachraum und auf der Normalstation. Die Initialen des Patienten oder eine andere Kodierung (zum Beispiel Teil der Fallnummer) können auf dem Prozessfragebogen eingetragen werden, um später die Zuordnung der Prozessparameter zu den Ergebnisparametern zu ermöglichen. Eine Dokumentation des Patientennamen auf dem Ergebnis-Fragebogen (Patientenfragebogen) ist nicht erlaubt, da es sich um eine anonymisierte Befragung handelt.

Hinweis: Zur gezielten Defizitanalyse von Bereichen (Stationen, Operationen, etc.), die über die Ergebnisparameter als problematisch identifiziert wurden, sind die Prozess-Parameter unverzichtbar. Die webbasierte Auswertung der gesamten Prozessparameter ist im Feedback des QUIPS-Basismoduls nicht

Copyright Nutzung durch Dritte vorbehaltlich Genehmigung durch DGAI/ DGCH Version 2018

enthalten. Darüber hinaus stehen diese Daten natürlich der erhebenden Einrichtung zur eigenen Auswertung in Form einer Excel oder SPSS- Datei zur Verfügung.

NARKOSE

N1 bis N4 erfassen Informationen zur Narkoseart, Regionalanästhesie, PONV- Prophylaxe, Wundinfiltration und intraoperativen Ko-Analgetika.

PRÄMEDIKATION

Was wurde zur Prämedikation gegeben?

P1 und P2 erfassen zur Prämedikation verabreichte Nicht-Opioide und Opioide. Eine Dosisangabe ist für P2 vorgesehen. Die Dosisangabe bezieht sich auf die applizierte Menge des Medikaments. Bitte beachten Sie bei der p. o.-Medikation die Unterscheidung zwischen retardierter und unretardierter Applikationsform.

AUFWACHRAUM

Welche systemische Schmerztherapie wurde im Aufwachraum seit der OP durchgeführt?

A1 bis A4 erfassen im Aufwachraum verabreichte Nicht-Opioide, Opioide, Ko-Analgetika und die Verwendung der PCA- Verfahren. Eine Dosisangabe ist für A2 vorgesehen. Die Dosisangabe bezieht sich auf die applizierte Menge des Medikaments. Bitte beachten Sie bei der p. o.-Medikation die Unterscheidung zwischen retardierter und unretardierter Applikationsform.

STATION

Welche systemische Schmerztherapie wurde auf der Station seit der OP durchgeführt?

S1 bis S4 erfassen auf der Station verabreichte Nicht-Opioide, Opioide, Ko-Analgetika und die Verwendung der PCA- Verfahren. Eine Dosisangabe ist für S2 vorgesehen. Die Dosisangabe bezieht sich auf die applizierte Menge des Medikaments. Bitte beachten Sie bei der p. o.-Medikation die Unterscheidung zwischen retardierter und unretardierter Applikationsform.
S5 erhebt, ob Katheter-Verfahren stationär zum Einsatz kamen. Unter S 5a kann das entsprechende Regionalanästhesieverfahren ausgewählt werden.
S6 dokumentiert, ob eine individuelle Therapieanordnung (d. h. eine individuell für diesen Patienten bestimmte Angabe zu Art und Dosis der Schmerztherapie) vorhanden ist, damit eine notwendige Therapie ohne Zeitverlust durch die Pflegenden eingeleitet werden kann.
S7 erfasst, ob mindestens einmal täglich eine Schmerzmessung postoperativ vorgenommen und in den Patientenunterlagen dokumentiert wurde.

3.4 Freifelder

Es stehen neun Freifelder zur Verfügung, die für individuelle Zusatzfragen genutzt werden können. Zum Beispiel für die Erhebung zusätzlicher Medikamente, Interventionen oder anderer Ergebnisparameter, die in der eigenen Klinik angewendet werden. Texteingaben sind nach Möglichkeit zu vermeiden, da die Auswertung der Daten in der Regel über eine numerische Kodierung vorgenommen wird. Die Kodierung sollte dem Personal zur Datenerhebung bekannt sein und konsequent verwendet werden.

Gern können Sie auch uns darüber informieren, wir koordinieren interessante Fragestellungen per Newsletter an die teilnehmenden Kliniken.

4. Datenerhebung

4.1 Randomisierung der Stationen

Oft wird es aus Kapazitätsgründen nicht möglich sein, eine Vollerhebung durchzuführen, also alle operierten Patienten auf allen teilnehmenden Stationen zu befragen. Dies ist allerdings auch nicht notwendig, wenn die Daten auch aus einer repräsentativen Stichprobe erhoben werden. Dazu sollte ein bestimmtes Prozedere eingehalten werden, um systematische Fehler zu vermeiden.
Wir empfehlen dazu folgendes Vorgehen:
Stehen nicht genug Ressourcen für eine Vollerhebung zur Verfügung, sollten täglich so viele Stationen ausgelost werden, wie Befragungskapazität vorhanden ist. Auf den ausgewählten Stationen sollten dann aber jeweils alle Patienten befragt werden, die die Einschlusskriterien erfüllen. Durch eine entsprechende Losverteilung kann eine ausreichende Berücksichtigung aller Stationen in bestimmten Zeiträumen gesichert werden.
Kommen auf der Station eine hohe Anzahl der Patienten in Frage, sollte man auch hier die Patienten auslosen/randomisieren.

Copyright Nutzung durch Dritte vorbehaltlich Genehmigung durch DGAI/ DGCH Version 2018

Natürlich sind auch andere Randomisierungsverfahren denkbar, es muss aber immer darauf geachtet werden, dass keine systematischen Fehler entstehen. Wird beispielsweise nur jeweils der erste Patient des OP-Programms befragt, wird es sich in vielen Fällen um Privatpatienten handeln, deren „Behandlungsprozesse“ sich in vielen Aspekten vom Durchschnitt unterscheiden (Chefarzt, Einzelzimmer etc.).

4.2 Ein- und Ausschlusskriterien

Die Befragung des Patienten ist möglichst am **ersten postoperativen Tag** auf der jeweiligen operativen Normalstation durchzuführen, dabei entspricht der Tag, an dem die Operation durchgeführt wurde, dem Tag Null,es gibt die Auswahl von Tag 0-5. Allerdings sollte man beachten, bei einer zu späten Befragung kann es zu einer systematischen Verzerrung der Ergebnisse kommen.
Der Beurteilungszeitraum integriert die Auswirkungen von der Operation und Narkose, der postoperativen Behandlung im Aufwachraum und vor allem die weitere postoperative Schmerztherapie auf der jeweiligen operativ geführten Station.
Für das erste Screening der Patienten, zum Beispiel anhand des Operationsplanes vom Vortag, gelten folgende **Einschlusskriterien** für das QUIPS-Basismodul:

- Der Einschluss in die Befragung erfolgt **ab dem 18. Lebensjahr**.
- Der Patient befindet sich postoperativ auf der **Normalstation**.
- Patienten, die mehrmals in Folge operiert werden müssen, werden nur **einmalig** während eines Krankenhausaufenthaltes befragt.
- Der Patient muss postoperativ auf einer **Station** betreut werden, die sich zu einer **Teilnahme am Projekt QUIPS** bereit erklärt hat.

Die ausgewählten Patienten werden im Anschluss an das Screening auf der Station aufgesucht und erneut evaluiert. Vor Beginn der Befragung muss das schriftliche Einverständnis für die QUIPS-Befragung des Patienten eingeholt werden. Der Patient erhält eine Kopie, das Original wird beim zuständigen QUIPS-Team verbleiben, bitte nicht in der Krankenakte abheften. Anschließend werden die demographischen Parameter (siehe 3.1) auf dem Fragebogen ergänzt und die Ausschlusskriterien anhand des Parameters D10 (siehe 3.1) überprüft. Im Folgenden sind Originalzitate aus dem Fragebogen grün markiert.

D10 Einschluss des Patienten nicht möglich aus folgendem Grund:

☐ Patient befindet sich auf einer anderen Station
☐ Patient ist nicht anwesend/bereits entlassen
☐ Patient lehnt die Befragung ab
☐ Patient hat bereits teilgenommen
☐ Patient spricht kein Deutsch
☐ Patient ist verwirrt/hat Verständnisschwierigkeiten
☐ Patient ist sediert/schläft
falls ja, bitte auswählen: ☐ leicht schläfrig
☐ häufig müde, leicht erweckbar
☐ tief schlafend, schwer erweckbar (Bitte unbedingt Prozessparameter erheben!)
☐ nicht erweckbar (Bitte unbedingt Prozessparameter erheben!)
☐ Sonstige Gründe

Grundsätzlich

- ist ein **sprachliches und intellektuelles Verständnis** seitens des Patienten Voraussetzung für das eigenständige Ausfüllen des Fragebogens,
- der Patient soll **wach** und **voll orientiert** sein und
- muss seine **schriftliche Einwilligung zur Teilnahme an der Befragung gegeben haben.**

4.3 Datenerfassung

Identifizieren Sie die Patienten, die sich postoperativ für eine Befragung eignen (siehe Kapitel 4.1 und 4.2).
Die standardisierte Datenerhebung ist von **äußerster Wichtigkeit für die Datenqualität**! Es sollte alles vermieden werden, was dazu führen könnte, dass der Patient nicht „ehrlich“ antworten würde. So sollte das Zimmer verlassen werden (diese Zeit bietet sich dafür an, die Prozess-Daten zu erheben), es sollte kein Zeitdruck bestehen. Oft bieten sich Angehörige zur Mithilfe an, zum Beispiel den Fragebogen vorzulesen und gemeinsam auszufüllen. Nach Möglichkeit sollte diese Situation vermieden werden und die Befragung durch das geschulte Personal erfolgen. Auf keinem Fall sollten die Angehörigen dem Patienten die

Copyright Nutzung durch Dritte vorbehaltlich Genehmigung durch DGAI/ DGCH Version 2018

Antworten „diktieren“ etc. Die Patienten benötigen im Durchschnitt 10 - 15 Minuten, um den Ergebnis-Fragebogen auszufüllen. Beim Abholen des Fragebogens kann nachgefragt werden, ob der Patient alle Fragen ausgefüllt hat und ob Fragen oder Schwierigkeiten beim Ausfüllen auftraten. Hilfestellungen beim Ausfüllen sollten keinen Einfluss auf die Antwort des Patienten nehmen.
Für die Befragung der zur Auswahl stehenden Patienten sind die Fragen D1 bis D9 immer auszufüllen. Die Informationen zu den Fragen D1 bis D11 sind der Krankenakte oder dem elektronischen Patienten-Informationssystem zu entnehmen.
Zur Beantwortung der Frage D10 muss der Patient in seinem Zimmer aufgesucht werden. Der Patient muss, wie erwähnt, zu diesem Zeitpunkt über das Projekt aufgeklärt werden. Hierfür bietet sich folgende Vorstellung an:

„Guten Tag, Frau/Herr XY, mein Name ist Z. Wir führen an dieser Klinik eine Erhebung zur Schmerztherapie nach Operationen durch. Sie soll dazu dienen, die Schmerztherapie weiter zu verbessern. Daher möchte ich Sie bitten, einige Fragen zu beantworten. Die Teilnahme ist freiwillig, falls Sie Interesse an der Teilnahme haben, bitten wir Sie, dieses Einverständnis durchzulesen und zu unterschreiben. Alle Daten werden absolut vertraulich behandelt und anonymisiert. Ich selber gehöre nicht zu Ihrem Behandlungsteam. Sind Sie damit einverstanden?“

Möchte der Patient an der Befragung teilnehmen, das schriftliche Einverständnis ist erteilt, übergibt man den Ergebnisfragebogen an den Patienten.
Manche Patienten benötigen eine kurze Erklärung der Fragen, (siehe Anleitung unter 3.2), sollte dies nicht ausreichen, lässt man die Frage offen und gibt später in der Datenmaske „nicht erhebbar“ ein.
Es ist von großer Wichtigkeit, die Daten sorgfältig von dem Papierbogen in die Datenmaske zu übertragen und keine Änderungen vorzunehmen. Es kann passieren, dass ein Patient das Kreuz zwischen 2 Zahlen setzt. In diesem Fall soll man immer den schlechteren Wert eingeben, bei dem NRS-Wert den höheren Zahlenwert, bei der Zufriedenheit den niedrigeren Zahlenwert.

Kann ein Patient nicht in die Befragung eingeschlossen werden, so ist dies unter dem Punkt D10 auf dem Fragebogen zu dokumentieren. Die Befragung für diesen Patienten ist an dieser Stelle beendet. Sollte der Einschluss des Patienten aufgrund einer Sedierung nicht möglich sein, empfehlen wir die Erhebung der Prozessdaten vorzunehmen, um mögliche Rückschlüsse auf die Nebenwirkungen der postoperativen Schmerztherapie zu erhalten und damit einer Auswertung zuzuführen.

Fehlende Daten: Bei fehlenden Unterlagen, zum Beispiel ein nichtauffindbares Narkoseprotokoll oder die Akte ist schon im Sekretariat um den Abschlussbericht zu schreiben, haben Sie bei allen Parametern die Möglichkeit, „nicht erhebbar“ einzugeben. Auch wenn der Patient eine oder mehrere Fragen nicht beantwortet hat, geben Sie bitte „nicht erhebbar“ in die Datenmaske ein.

4.3.1 Online Dateneingabe durch den Patienten

Wenn ein Patient den Fragebogen im Browser eingeben soll, öffnen Sie wie gewöhnlich einen Fragebogen in der Webmaske. Bei den demographischen Parametern finden Sie unter **D11 die Option "E-Mail"**. Hier haben Sie nun 2 Optionen:

1. Patient benutzt ein **eigenes Gerät**

- Sie geben in das Email-Feld die E-Mail-Adresse des Patienten ein und drücken "Senden".
- Der Patient erhält dann eine E-Mail mit einem Link. Wenn er darauf klickt, öffnet sich der Fragebogen im Browser seines Geräts (Computer, Laptop, Tablet, Smartphone). Er kann die Patientenfragen ausfüllen und den Fragebogen speichern. Danach sind die Daten in der QUIPS-Datenbank vorhanden.

2. Benutzung eines **klinikeigenen Tablets**

- Sie benutzen ein klinikeigenes Tablet mit Internetanschluss.
- Geben Sie in das Email-Feld eine E-Mail-Adresse an, auf die Sie Zugriff haben (z.B. eine Stations-Adresse oder eine extra für diesen Zweck erstellte Adresse bei einem E-Mail-Anbieter). Klicken Sie auf "Senden".
- Öffnen Sie die E-Mail und klicken Sie auf den Link. Der Browser mit dem Fragebogen öffnet sich.
- Geben Sie nun dem Patienten das Gerät. Wenn Sie es zurückerhalten, achten Sie darauf, dass der Patient "Speichern" geklickt hat. Der Fragebogen ist nun in der QUIPS-Datenbank vorhanden.
- Reinigen Sie das Gerät mit Desinfektionstüchern.

Copyright Nutzung durch Dritte vorbehaltlich Genehmigung durch DGAI/ DGCH Version 2018

- Beachten Sie, dass die Patienten evtl. Zugriff auf weitere Funktionen Ihres Gerätes haben. Vermeiden Sie also, datenschutz-relevante Informationen darauf zu speichern.

Die hier eingegebene E-Mail-Adresse wird nicht gespeichert. Der Patient hat nach Versendung der E-Mail 6 Stunden Zeit, den Fragebogen auszufüllen. Danach funktioniert der Link nicht mehr.

Text der automatischen Mail:

Sehr geehrte Damen und Herren,

Während Ihres Krankenhausaufenthalts haben Sie sich freundlicherweise bereit erklärt, an einer Befragung zu Ihrem Befinden nach Ihrer Operation teilzunehmen. Wir möchten untersuchen, wie Patienten sich am ersten Tag nach der Operation fühlen. Vielen Dank für Ihre Bereitschaft, an unserer Befragung teilzunehmen! Mit Ihren Angaben helfen Sie uns, die Schmerztherapie nach Operationen zu verbessern.

Diese E-Mail informiert Sie über die weitere Vorgehensweise in der Befragung.

Bitte klicken Sie auf den folgenden Link. Der Fragebogen sollte dann in Ihrem Browserfenster erscheinen. Alle notwendigen Informationen finden Sie auf dieser Website. Wir versichern Ihnen nochmals, dass Ihre Angaben vollkommen anonym sind.

Bitte denken Sie daran, dass Ihre Einschätzungen sich ausschließlich auf die Schmerzen im Zusammenhang mit Ihrer Operation beziehen sollen. Geben Sie bitte keine Schmerzen oder Beeinträchtigungen an, die keinen Bezug zu Ihrer Operation haben (z.B. Rückenschmerzen, Arthritis oder Kopfweh).

Nochmals vielen Dank für Ihre Teilnahme an dieser Befragung!

https://bms.med.uni-jena.de/cgi-bin/WebObjects/BmsQuips.woa/wa/qnQuips?uid=57WKS81K12&lang=de

4.4 Langzeit- Follow-up/ Chronifizierung postoperativer Schmerzen

Erstmalig in unserem Fachgebiet bietet QUIPS die Möglichkeit, Patienten 6 und 12 Monate postoperativ webbasiert zu langfristigen Ergebnissen der Schmerztherapie zu evaluieren! Somit können Sie die langfristigen Auswirkungen der Operation und Ihrer Schmerztherapie beobachten.

4.4.1 Datenerhebung für das Langzeit Follow up

Erheben Sie wie unter Punkt 4 beschrieben Ihre QUIPS-Daten. Fragen Sie dann den Patienten, ob er mit einer Nachbefragung nach 6 und 12 Monaten einverstanden ist. Falls ja, übergeben Sie die Patienteninformation, auf der der Patient seine E-Mail-Adresse und/oder Telefonnummer notiert und sein Einverständnis mit Unterschrift quittiert. Ein Beispiel für die Einverständniserklärung finden Sie auf unserer Homepage unter „Infothek“. Die komplette Eingabe wird im Kapitel 4.5 beschrieben.

Tragen Sie wie gewohnt die erhobenen QUIPS-Daten in die Datenmaske ein. In der ersten Karteikarte "Demographische Parameter" befindet sich der Punkt "Nachbefragung". Aktivieren Sie dort das Häkchen und fügen Sie die E-Mail-Adresse des Patienten ein. Falls Sie nur die Telefonnummer haben, tragen Sie diese ein und in das Mail-Feld Ihre eigene E-Mail-Adresse (Abb.6).
Wenn Sie die Adresse eingetragen haben, erhält der Patient automatisch nach 6 und 12 Monaten eine Mail. In dieser befindet sich ein Link, der ihn zur Nachbefragung führt. Falls der Patient diese nicht ausfüllt, wird er nochmal automatisch erinnert.

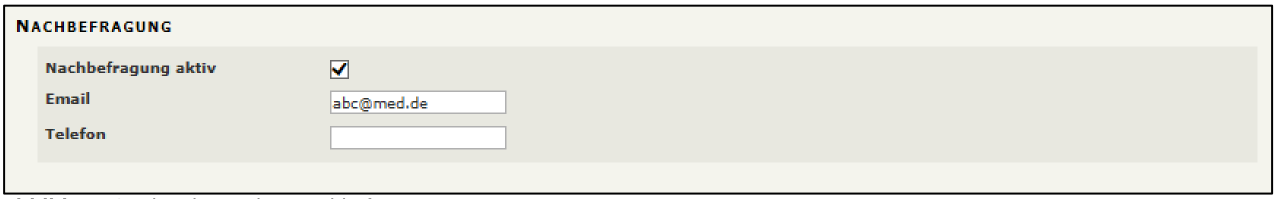

Abbildung 6: Eingabemaske „Nachbefragung“

Copyright Nutzung durch Dritte vorbehaltlich Genehmigung durch DGAI/ DGCH Version 2018

Wenn Sie die Telefonnummer und Ihre Mail-Adresse eingetragen haben, dann erhalten Sie eine Mail mit dem Link und der Telefonnummer. Dann rufen Sie den Patienten an und füllen den Fragebogen online aus. Das ist für Sie also etwas aufwändiger.

Die Fragebögen, die nach 6 und 12 Monaten benutzt werden, sind der DN4 (neuropathischer Schmerz) und der Brief Pain Inventory (gesundheitsbezogene Lebensqualität). Beide sind validiert und werden in der deutschen Übersetzung abgefragt.

Normalerweise werden QUIPS-Patienten sofort anonymisiert. Das ist hier nicht so, da Sie Kontaktdaten erheben und wir diese speichern müssen. Diese Kontaktdaten werden in einer separaten Datentabelle gespeichert. Sie können also nicht direkt, aber indirekt QUIPS-Daten zugeordnet werden. Nach der Erhebung werden die Kontaktdaten gelöscht. Dennoch sollten Sie, bevor Sie mit der Nachbefragung beginnen, ein **Ethik-Votum** einholen und wie schon beschrieben die Patienten informieren und sich ihre Teilnahme schriftlich bestätigen lassen.

4.5 Eingabe und Transfer der Daten zum zentralen Server

Die papiergebundenen Daten müssen in die Eingabemaske am Benchmarkserver eingegeben werden. Dafür ist eine Internet-Verbindung nötig. Die Daten können Sie über unsere Homepage www.quips-projekt.de unter dem Menüpunkt Applikation „Fragebogen/Benchmark“ eingeben. Ihre Benutzerkennung und Ihr Passwort wird Ihnen nach Vertragsabschluss von der kooperierenden Firma TAKWA zur Verfügung gestellt.
Über den Link „Fragebogen“ werden sie nun zum Login der Eingabemaske weitergeleitet. Geben Sie bitte Ihren Benutzernamen und Ihr zugewiesenes Passwort ein und bestätigen Sie die Anmeldung indem Sie auf „Login“ klicken. Sollten hierbei irgendwelche Probleme auftreten oder weiterführende Hilfestellungen benötigt werden, wenden Sie sich bitte an das Team der Firma TAKWA.

Nach dem erfolgreichen Login öffnet sich die nächste Seite, in der Sie bitte den Menüpunkt „QUIPS Basis“ aus der Liste auswählen. Wir empfehlen von einer automatischen Speicherung des Passwortes abzusehen, um zu verhindern, dass Unbefugte Zugriff auf Ihre Daten erhalten.
Jetzt öffnet sich das Fenster für die Suchmaske und die Liste der vorhandenen Datensätze.

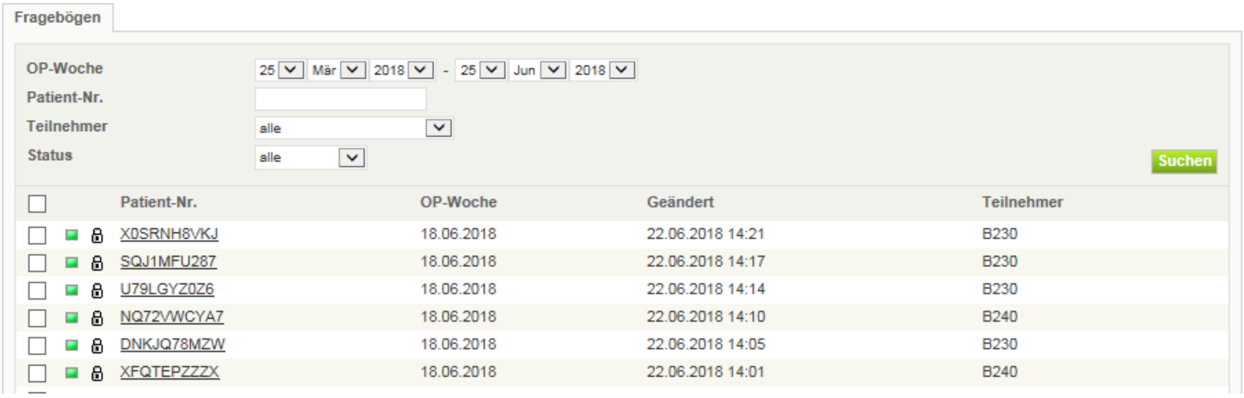

Abbildung 7: Suchmaske und Liste bereits gespeicherter Daten

Aus Gründen des Datenschutzes und der Anonymisierung der Daten wird für jeden gespeicherten Datensatz eine 10-stellige Nummer per Zufall generiert.
Für die Suche von Datensätzen haben Sie mehrere Optionen, suchen Sie zum Beispiel einen bestimmten Fragebogen, geben Sie die zugehörige Nummer ein und schränken Sie die Suche zusätzlich ein, indem Sie den Zeitraum eingrenzen und die zugehörige Station auswählen.
Eine weitere Suchfunktion ermöglicht es, anhand der Statussuche, noch zu bearbeitende Fragebögen anzuzeigen. Der Status der Eingabe ist farblich gekennzeichnet.

Symbol : Dieser Datensatz ist gespeichert, es besteht die Möglichkeit ihn weiter zu bearbeiten. So kann man die Daten von unterschiedlichen Arbeitsplätzen eingeben. Änderungen und Nachträge sind möglich.

Copyright Nutzung durch Dritte vorbehaltlich Genehmigung durch DGAI/ DGCH Version 2018

Symbol : Dieser Datensatz ist abgeschlossen, d. h. es sind keine Änderungen mehr vorzunehmen. Diese Daten sind bereits an den zentralen Server weitergeleitet worden und stehen für ein Feedback zur Verfügung.

Symbol : In diesem Datensatz sind die Ergebnisparameter unvollständig. Es besteht die Möglichkeit, falls der Datensatz noch nicht abgeschlossen ( ) ist, die fehlenden Ergebnisparameter zu vervollständigen (siehe Karteikarte: Ergebnis).

Symbol : In diesem Datensatz sind die Ergebnisparameter vollständig. Endet die Befragung an Punkt D10, d. h. der Patient wird von der Befragung ausgeschlossen, erhält der Datensatz auch das grüne Symbol.

Haben Sie einen fehlerhaften Datensatz angelegt, setzen Sie einen Haken in dem Kästchen vor dem entsprechenden Datensatz und wählen den Button „Löschen“.

Hinweis: Sie können in der Liste der Fragebögen den Status erkennen und die jeweiligen Daten vervollständigen und zum Abschluss bringen.

Unvollständige Datensätze können bis zu 3 Wochen nachbearbeitet werden, danach ist eine Editierung aus Plausibilitätsgründen nicht vorgesehen.

Um einen neuen Datensatz anzulegen, klicken Sie bitte auf den Button „Neuer Fragebogen“.
Die weitere Menüführung ist intuitiv erfassbar und in Form von Karteikarten aufgebaut. Es existieren so genannte Reiter für die jeweiligen Bereiche (Karteikarten) des QUIPS- Fragebogens:

- **Demographische Parameter**
- **Ergebnis** (Patientenfragebogen)
- **Narkose** (Prozessdaten)
- **Prämedikation** (Prozessdaten)
- **Aufwachraum** (Prozessdaten)
- **Station** (Prozessdaten)
- **Freifelder** (individuelle Fragestellungen)

Die Karteikartenreiter befinden sich am oberen und am unteren Rand, um so die Navigation in der Eingabemaske zu vereinfachen. Aus dem gleichen Grund befinden sich der Button zum „Speichern“ und der Button „Neuer Fragebogen“ jeweils am Anfang jeder Karteikarte und an deren Ende.
Haben Sie die Daten in alle Karteikarten eines Datensatzes vollständig eingegeben und diese überprüft, können Sie diesen Datensatz abschließen. Dafür klicken Sie bitte auf den Button „Abschließen“, zur Sicherheit wird noch einmal nachgefragt, ob der Fragebogen wirklich abgeschlossen werden soll.
Ein abgeschlossener Datensatz ist in der Liste mit dem Symbol des verriegelten Schlosses markiert und kann nicht mehr nachträglich editiert werden. Diese Daten befinden sich nun in der zentralen Datenbank und stehen für das Feedback via Benchmarkserver zur Verfügung.

Für einige Eingabefelder sind die Antwortmöglichkeiten bereits vorgegeben, die aus den Untermenüs ausgewählt werden sollen. Um eventuell auftretende Fehleingaben zu vermeiden, sind verschiedene Plausibilitätskontrollen eingebaut, zum Beispiel bei der Eingabe der Dosierung ist es nur möglich Zahlen einzugeben und keine Buchstaben. Deshalb sollte bei einer Fehlermeldung die Richtigkeit der Eingabe der Daten überprüft werden.

Wichtig: Um Dezimalstellen einzugeben, verwenden Sie bitte das Komma – und nicht den Punkt!

Es wird empfohlen, nach dem Ausfüllen einer Karteikarte diese erst zu speichern und dann zur nächsten Karteikarte zu wechseln. Auf die Vollständigkeit der eingegebenen Daten ist stets zu achten.

Karteikarte: Demographische Parameter

Zu Beginn wird auf dieser Karteikarte die automatisch generierte 10-stellige Nummer für den aktuellen Datensatz in grüner Schrift angezeigt. Notieren Sie sich diese Nummer auf den Fragebogen des zugehörigen Datensatzes, so können Sie später mit Hilfe der Suchfunktion den Datensatz noch einmal aufrufen und gegebenenfalls Ergänzungen vornehmen.

Copyright Nutzung durch Dritte vorbehaltlich Genehmigung durch DGAI/ DGCH Version 2018

Die Auswahl der zugehörigen Station ist bereits rot eingerahmt und stellt ein so genanntes Pflichtfeld dar. Wird aus Versehen ein rot umrandetes Feld = Pflichtfeld nicht ausgefüllt, wird man an der weiteren Eingabe der Daten gehindert. Die Pflichtfelder enthalten wesentliche Informationen, die für die Zuordnung und die weitere Analyse der Daten notwendig sind.

Für den Parameter „**Geschlecht**“ klicken Sie die jeweils zutreffende Antwort an. Bei einigen Parametern, wie zum Beispiel „**Alter**“ des Patienten, öffnet sich ein Pull-down-Menü und die zutreffende Antwort, in diesem Beispiel die entsprechende Altersdekade, wird durch einmaliges Klicken ausgewählt.
Anschließend werden die restlichen demographischen Parameter und die Kodierung der Operation (OPS) mit der Schnitt-Naht-Zeit vervollständigt.
Sollte ein Patient aus irgendeinem Grund nicht an der Befragung teilnehmen, wählen Sie im Pull-down-Menü die zutreffende Antwort aus (D10). Eine Eingabe der Ergebnisparameter ist somit nicht möglich und die Karteikarten für die Ergebnisparameter und Prozessdaten werden ausgeblendet. Der Datensatz ist vollständig und kann abgeschlossen werden.
Fällt der Patient auf Grund einer Sedierung aus („sediert/schläft“), wählt man zusätzlich den Grad der Sedierung aus dem Untermenü und kann die zugehörigen Prozessparameter eintragen. So können mögliche Nebenwirkungen durch die postoperative Schmerztherapie erfasst werden.

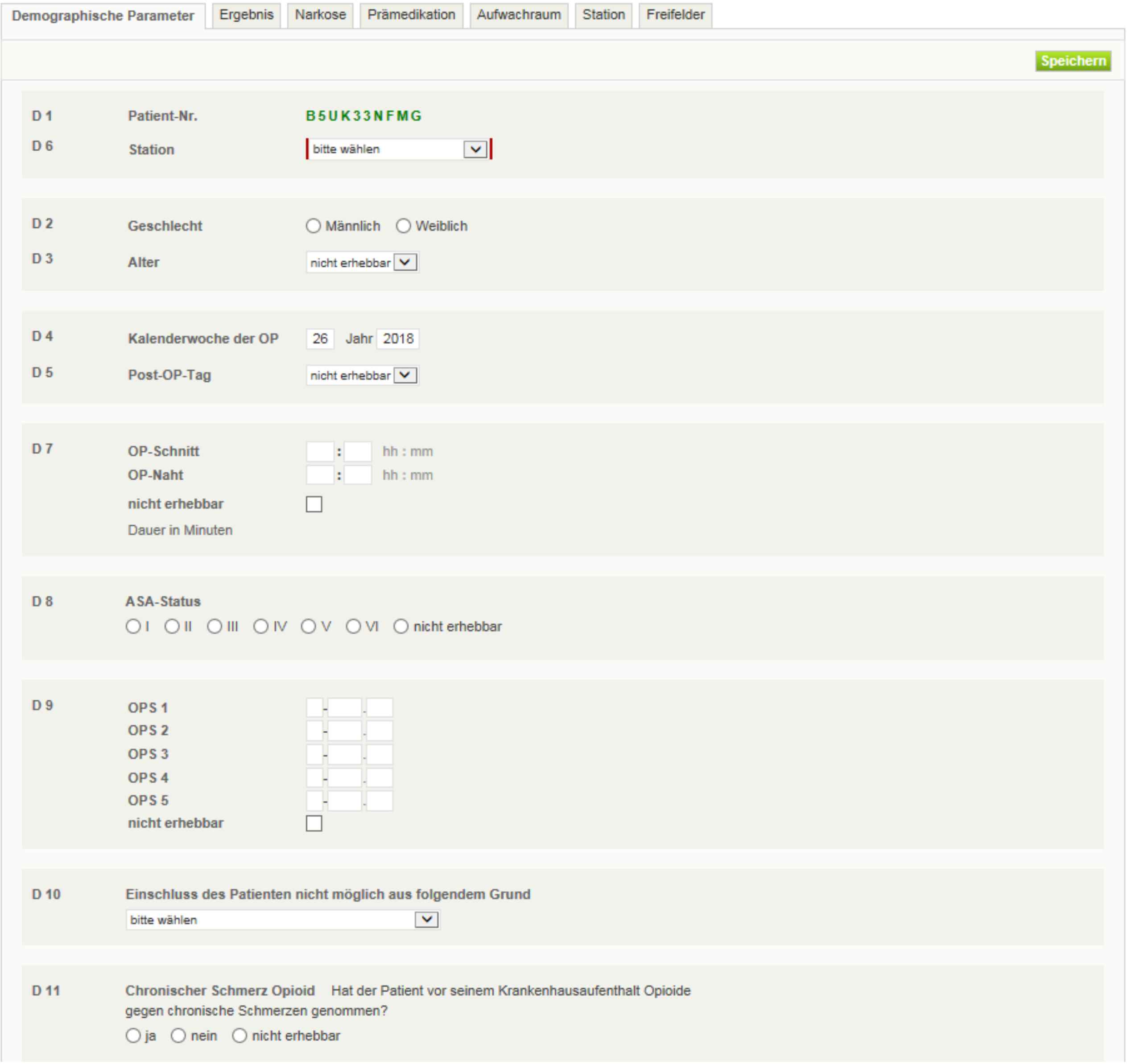

Abbildung 8: Fragebogen Demographische Parameter

Copyright Nutzung durch Dritte vorbehaltlich Genehmigung durch DGAI/ DGCH Version 2018

Karteikarte: **Ergebnis** (siehe Abb. 9)
Hier haben Sie die Möglichkeit die Ergebnisparameter einzugeben. Im Vordergrund des QUIPS-Projektes stehen die Outcome-Parameter aus Sicht des Patienten, deshalb sind diese Felder (E1 - E16) als so genannte Hinweisfelder markiert und durch eine orangefarbene Umrandung gekennzeichnet. Falls der Patient sich zu einem Punkt Fragen mit der der Ja /nein Antwortmöglichkeit nicht geäußert hat, wählt man „nicht erhebbar“. Die Angaben zur Schmerzintensität (NRS), Zufriedenheit mit der Schmerztherapie sowie die Einbeziehung Entscheidungen zu der Schmerztherapie werden in die Felder E1- E3, E12, E14 und E16a; E13 und E15 übertragen. Haben Sie aus Versehen eine falsche Angabe gemacht, kann die Eingabe mit dem Button „Auswahl aufheben“ wieder aufgehoben bzw. gelöscht werden.

Nachdem alle Daten der Karteikarte Ergebnis eingeben sind, gehen Sie bitte auf „Speichern“, es sollten alle orangefarbenen Umrandungen verschwunden sein. Äußert sich ein Patient nicht zu den Punkten E2 - E4, E13 und E15 bleibt die orangefarbene Umrandung bestehen, welches dazu führt, dass der abgespeicherte Fragebogen mit dem Status „rot“ gekennzeichnet wird. Dies hat aber keinen Einfluss auf die weitere Dateneingabe. Der Datensatz ist weiter zu bearbeiten und nach Überprüfung der Daten abzuschließen.

Copyright Nutzung durch Dritte vorbehaltlich Genehmigung durch DGAI/ DGCH Version 2018

E 1 **Schmerz bei Belastung** Wie stark waren Ihre Schmerzen seit der Operation, wenn Sie sich belastet haben, zum Beispiel bei Mobilisierung, Bewegen, Waschen, Husten, Durchatmen?
○ 0 ○ 1 ○ 2 ◉ 3 ○ 4 ○ 5 ○ 6 ○ 7 ○ 8 ○ 9 ○ 10 Auswahl aufheben

E 2 **Stärkster Schmerz** Wie stark waren bisher Ihre stärksten Schmerzen seit der Operation?
○ 0 ○ 1 ○ 2 ○ 3 ◉ 4 ○ 5 ○ 6 ○ 7 ○ 8 ○ 9 ○ 10 Auswahl aufheben

E 3 **Geringster Schmerz** Wie stark waren bisher Ihre geringsten Schmerzen seit der Operation?
○ 0 ◉ 1 ○ 2 ○ 3 ○ 4 ○ 5 ○ 6 ○ 7 ○ 8 ○ 9 ○ 10 Auswahl aufheben

E 4 **Bewegung** Sind Sie durch die Schmerzen in Ihrer Mobilität bzw. Bewegung beeinträchtigt?
◉ ja ○ nein ○ nicht erhebbar

E 5 **Husten und Luftholen** Sind Sie durch die Schmerzen beim Husten oder tiefen Luftholen beeinträchtigt?
◉ ja ○ nein ○ nicht erhebbar

E 6 **Schlaf** Sind Sie durch die Schmerzen heute Nacht aufgewacht?
○ ja ◉ nein ○ nicht erhebbar

E 7 **Stimmung** Sind Sie durch die Schmerzen in Ihrer Stimmung beeinträchtigt?
○ ja ◉ nein ○ nicht erhebbar

E 8 **Müdigkeit** Haben Sie sich seit der Operation sehr müde gefühlt?
○ ja ◉ nein ○ nicht erhebbar

E 9 **Übelkeit** Haben Sie seit der Operation unter Übelkeit gelitten?
◉ ja ○ nein ○ nicht erhebbar

E10 **Schwindel** Haben Sie seit der Operation unter Schwindel gelitten?
○ ja ◉ nein ○ nicht erhebbar

E 11 **Aufklärung** Wurden Sie über die verschiedenen Möglichkeiten Ihrer Schmerztherapie informiert?
◉ ja ○ nein ○ nicht erhebbar

E 12 **Beteiligung** Wurden Sie in dem von Ihnen gewünschten Maß an Entscheidungen zu Ihrer Schmerztherapie beteiligt?
○ 0 ○ 1 ○ 2 ○ 3 ○ 4 ○ 5 ○ 6 ◉ 7 ○ 8 ○ 9 ○ 10 Auswahl aufheben

E 13 **Mehr Schmerztherapie** Hätten Sie sich mehr Schmerztherapie gewünscht, als Sie erhalten haben?
○ ja ◉ nein ○ nicht erhebbar

E 14 **Zufriedenheit** Wie zufrieden sind Sie mit der Schmerzbehandlung seit der Operation?
○ 0 ○ 1 ○ 2 ○ 3 ○ 4 ○ 5 ○ 6 ○ 7 ○ 8 ◉ 9 ○ 10 Auswahl aufheben

E 15 **Nicht medikamentöse Methoden** Haben Sie nicht-medikamentöse Methoden zur Schmerzlinderung benutzt oder erhalten?
◉ ja ○ nein ○ nicht erhebbar

☑ Kühlkompressen	☐ Wärme	☐ Meditation	☐ Tiefes Atmen
☐ Akupunktur	☐ Beten	☐ Umhergehen	☐ Massage
☑ Ablenkung	☐ Vorstellungsbilder	☐ Entspannung	☐ TENS
☐ Gespräche mit medizinischem Personal	☐ Gespräche mit Freunden oder Verwandten		

E 16 **Chronischer Schmerz** Hatten Sie ständige Schmerzen, die drei Monate oder länger andauerten, bevor Sie wegen dieser Operation ins Krankenhaus gekommen sind?
○ ja ◉ nein ○ nicht erhebbar

Vom Befragenden auszufüllen
☐ Patient wurde interviewt

Abbildung 9: Fragebogen Ergebnisparameter

Copyright Nutzung durch Dritte vorbehaltlich Genehmigung durch DGAI/ DGCH Version 2018

Karteikarte: Narkose (siehe Abb.10)
Unter N1 wählt man die Narkoseart aus, welche der Patient erhalten hat. Wählt man Regionale Anästhesie oder Allgemein- mit Regionalanästhesie aus, öffnet sich automatisch Punkt N2, die Lokalisation der Regionalanästhesie ist zu dokumentieren. Diese Eingabe ist unbedingt zu vervollständigen (Pflichtfeld). Bei Punkt N3 haben Sie die Möglichkeit „nicht erhebbar" zu wählen, wenn keine Daten dazu vorliegen. Nach der Kontrolle auf Vollständigkeit der Karteikarte Narkose bitte den Button „Speichern" drücken.

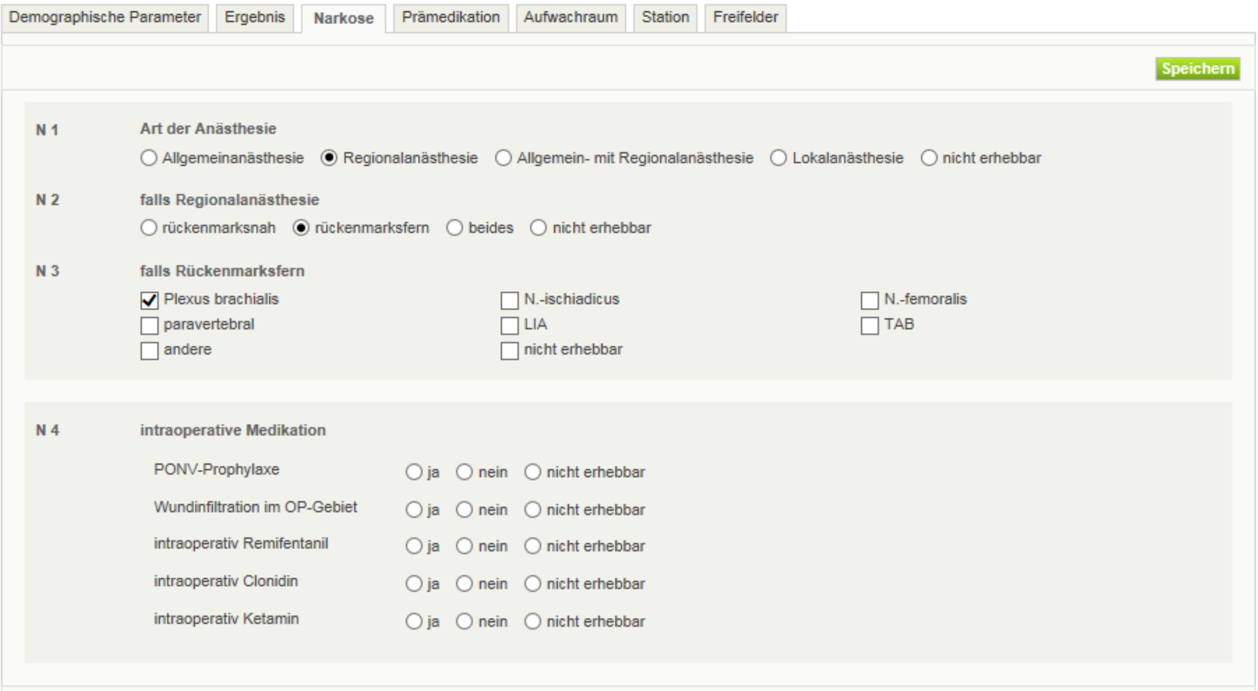

Abbildung 10: Fragebogen Prozessparameter: Narkose

Copyright Nutzung durch Dritte vorbehaltlich Genehmigung durch DGAI/ DGCH Version 2018

Karteikarte: Prämedikation (siehe Abb. 11)
Unter P1 und P2 werden die Medikamente zur Prämedikation erfasst. Unter P1, den Nicht- Opioiden werden nur die Applikationsformen erfasst. Unter P2 ist zu den jeweiligen Applikationsformen der Medikamente auch die Dosis erforderlich. Kombinationspräparate sind mit einem * gekennzeichnet; hier soll nur die Dosis des Opioids eingetragen werden (siehe auch 4.6, Beispiel 2). Fentanyl p.o. unretard wird als muccosale Applikation und Sufentanil p.o. unretard als sublinguale Applikation in diese Spalten eingetragen. Nach der Kontrolle auf Vollständigkeit der Karteikarte Prämedikation bitte den Button „Speichern“ drücken.

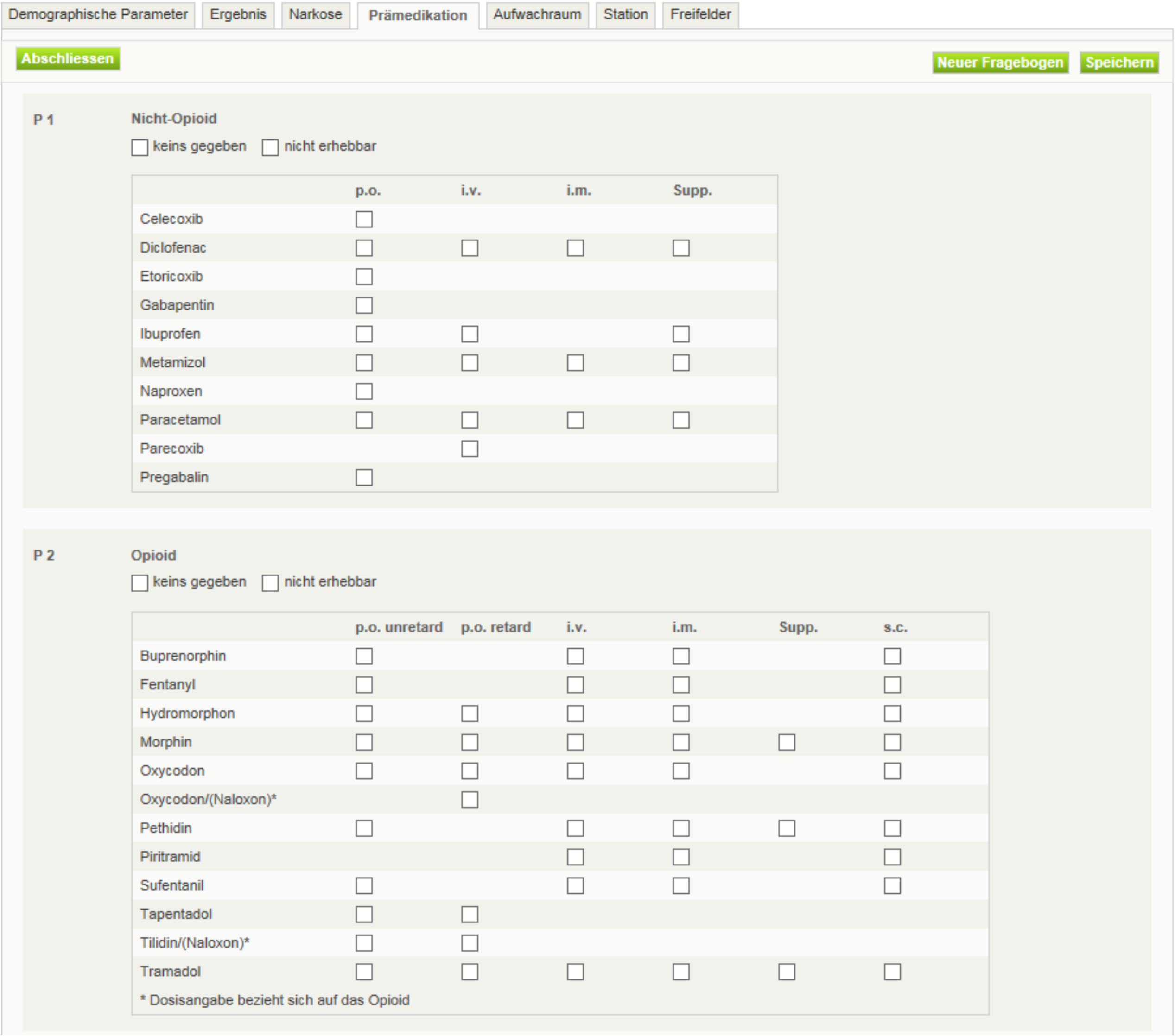
Demographische Parameter | Ergebnis | Narkose | Prämedikation | Aufwachraum | Station | Freifelder

Abschliessen — Neuer Fragebogen — Speichern

P 1 Nicht-Opioid

☐ keins gegeben ☐ nicht erhebbar

	p.o.	i.v.	i.m.	Supp.
Celecoxib	☐			
Diclofenac	☐	☐	☐	☐
Etoricoxib	☐			
Gabapentin	☐			
Ibuprofen	☐	☐		☐
Metamizol	☐	☐	☐	☐
Naproxen	☐			
Paracetamol	☐	☐	☐	☐
Parecoxib		☐		
Pregabalin	☐			

P 2 Opioid

☐ keins gegeben ☐ nicht erhebbar

	p.o. unretard	p.o. retard	i.v.	i.m.	Supp.	s.c.
Buprenorphin	☐		☐	☐		☐
Fentanyl	☐		☐	☐		☐
Hydromorphon	☐	☐	☐	☐		☐
Morphin	☐	☐	☐	☐	☐	☐
Oxycodon	☐	☐	☐	☐		☐
Oxycodon/(Naloxon)*		☐				
Pethidin	☐		☐	☐	☐	☐
Piritramid			☐	☐		☐
Sufentanil	☐		☐	☐		☐
Tapentadol	☐	☐				
Tilidin/(Naloxon)*	☐	☐				
Tramadol	☐	☐	☐	☐	☐	☐

* Dosisangabe bezieht sich auf das Opioid

Abbildung 11: Fragebogen Prozessparameter: Prämedikation

Copyright Nutzung durch Dritte vorbehaltlich Genehmigung durch DGAI/ DGCH Version 2018

Karteikarte: **Aufwachraum** (siehe Abb. 12)
Hier werden die Medikamente zur Schmerzlinderung im Aufwachraum erhoben. Kombinationspräparate sind mit einem * gekennzeichnet; hier soll nur die Dosis des Opioids eingetragen werden. Fentanyl p.o. unretard wird als muccosale Applikation und Sufentanil p.o. unretard als sublinguale Applikation in diese Spalten eingetragen.
A4 bezieht sich auf die patientenkontrollierte Analgesie (PCA) und deren Applikationsformen. Unterschieden werden die intravenöse Gabe (PCIA), die epidurale Gabe (PCEA), die regionale Gabe, bzw. die Applikation an peripheren Nerven (PCRA) und die sublinguale Gabe. Die Opioiddosis der i.v. und sublingualen PCA Verfahren bitte unter A2 entsprechend dem Medikament eintragen.
Nach der Kontrolle auf Vollständigkeit der Karteikarte Aufwachraum bitte den Button „Speichern" drücken.

Demographische Parameter | Ergebnis | Narkose | Prämedikation | Aufwachraum | Station | Freifelder

Speichern

A 1 **Nicht-Opioid**

☐ keins gegeben ☐ nicht erhebbar

	p.o.	i.v.	i.m.	Supp.
Celecoxib	☐			
Diclofenac	☐	☐	☐	☐
Etoricoxib	☐			
Gabapentin	☐			
Ibuprofen	☐	☐		☐
Metamizol	☐	☐	☐	☐
Naproxen	☐			
Paracetamol	☐	☐	☐	☐
Parecoxib		☐		
Pregabalin	☐			

A 2 **Opioid**

☐ keins gegeben ☐ nicht erhebbar

	p.o. unretard	p.o. retard	i.v.	i.m.	Supp.	s.c.
Buprenorphin	☐		☐	☐		☐
Fentanyl	☐		☐	☐		☐
Hydromorphon	☐	☐	☐	☐		☐
Morphin	☐	☐	☐	☐	☐	☐
Oxycodon	☐	☐	☐	☐		☐
Oxycodon/(Naloxon)*		☐				
Pethidin	☐		☐	☐	☐	☐
Piritramid			☐	☐		☐
Sufentanil	☐		☐	☐		☐
Tapentadol	☐	☐				
Tilidin/(Naloxon)*	☐	☐				
Tramadol	☐	☐	☐	☐	☐	☐

* Dosisangabe bezieht sich auf das Opioid

A 3 **Ko-Analgetika**

☐ keins gegeben ☐ nicht erhebbar

	p.o.	i.v.	i.m.	s.c.
Clonidin	☐	☐	☐	☐
Ketamin		☐	☐	☐
Lidocain		☐		

A 4 **Mit PCA**

○ PCIA (intravenös) ○ PCEA (epidural) ○ PCRA (regional) ○ PCA sublingual ○ keine erhalten ○ nicht erhebbar

Abbildung 12: Fragebogen Prozessparameter: Aufwachraum

Copyright Nutzung durch Dritte vorbehaltlich Genehmigung durch DGAI/ DGCH Version 2018

Karteikarte: **Station** (siehe Abb. 13)

Hier werden die Medikamente zur Schmerzlinderung auf der peripheren Station erhoben. . Kombinationspräparate sind mit einem * gekennzeichnet; hier soll nur die Dosis des Opioids eingetragen werden. Fentanyl p.o. unretard wird als muccosale Applikation und Sufentanil p.o. unretard als sublinguale Applikation in diese Spalten eingetragen.
S4 bezieht sich auf die patientenkontrollierte Analgesie (PCA) und deren Applikationsformen. Im stationären Setting stehen zur Auswahl die intravenöse (PCIA), die epidurale (PCEA), die regionale Analgesie (PCRA), und die sublinguale Gabe. Die Opioiddosis der i.v. und sublingualen PCA Verfahren bitte unter S2 entsprechend dem Medikament eintragen.
Unter S5 wird die durchgeführte Regionalanästhesie auf der peripheren Station erfasst. Trifft man die Auswahl „rückenmarksfern“ öffnet sich ein Menüfenster und man kann das spezielle Verfahren auswählen.
Unter S7 und S8 wird dokumentiert, ob eine individuelle Therapieanordnung für den Patienten vorlag und eine Schmerzdokumentation in der Patientenakte auf der Station vorgenommen wurde.
Nach der Kontrolle auf Vollständigkeit der Karteikarte Station bitte den Button „Speichern“ drücken.

Copyright Nutzung durch Dritte vorbehaltlich Genehmigung durch DGAI/ DGCH Version 2018

Demographische Parameter | Ergebnis | Narkose | Prämedikation | Aufwachraum | Station | Freifelder

Speichern

S 1 **Nicht-Opioid**

☐ keins gegeben ☐ nicht erhebbar

	p.o.	i.v.	i.m.	Supp.
Celecoxib	☐			
Diclofenac	☐	☐	☐	☐
Etoricoxib	☐			
Gabapentin	☐			
Ibuprofen	☐	☐		☐
Metamizol	☐	☐	☐	☐
Naproxen	☐			
Paracetamol	☐	☐	☐	☐
Parecoxib		☐		
Pregabalin	☐			

S 2 **Opioid**

☐ keins gegeben ☐ nicht erhebbar

	p.o. unretard	p.o. retard	i.v.	i.m.	Supp.	s.c.
Buprenorphin	☐		☐	☐		☐
Fentanyl	☐		☐	☐		☐
Hydromorphon	☐	☐	☐	☐		☐
Morphin	☐	☐	☐	☐	☐	☐
Oxycodon	☐	☐	☐	☐		☐
Oxycodon/(Naloxon)*		☐				
Pethidin	☐		☐	☐	☐	☐
Piritramid			☐	☐		☐
Sufentanil	☐		☐	☐		☐
Tapentadol	☐	☐				
Tilidin/(Naloxon)*	☐	☐				
Tramadol	☐	☐	☐	☐	☐	☐

* Dosisangabe bezieht sich auf das Opioid

S 3 **Ko-Analgetika**

☐ keins gegeben ☐ nicht erhebbar

	p.o.	i.v.	i.m.	s.c.
Clonidin	☐	☐	☐	☐
Ketamin		☐	☐	☐
Lidocain		☐		

S 4 **Mit PCA**

○ PCIA (intravenös) ○ PCEA (epidural) ○ PCRA (regional) ○ PCA sublingual ○ keins gegeben ○ nicht erhebbar

S 5 **Regionalanästhesie** Welche Regionalanästhesie (nur Katheterverfahren) wurde seit der Operation überwiegend durchgeführt?

○ rückenmarksnah ○ rückenmarksfern ○ keins gegeben ○ nicht erhebbar

S 6 **Therapieanordnung** Ist eine individuelle Therapieanordnung für die postoperative Schmerztherapie auf der Station vorhanden?

○ ja ○ nein

S 7 **Schmerzdokumentation** Ist eine Schmerzdokumentation (z.B. Kurve) auf der Station erfolgt?

○ ja ○ nein

Abbildung 13: Fragebogen Prozessparameter: Station

Copyright Nutzung durch Dritte vorbehaltlich Genehmigung durch DGAI/ DGCH Version 2018

Karteikarte: Freifeld (siehe Abb.14)
Sollten Sie individuelle Fragestellungen bearbeiten und dokumentieren wollen, können Sie dafür die so genannten Freifelder (F1 – F9) nutzen. Dokumentieren Sie sich den Zeitraum und die entsprechende Codierung für Ihre Zusatzfragestellungen in Ihren Unterlagen.

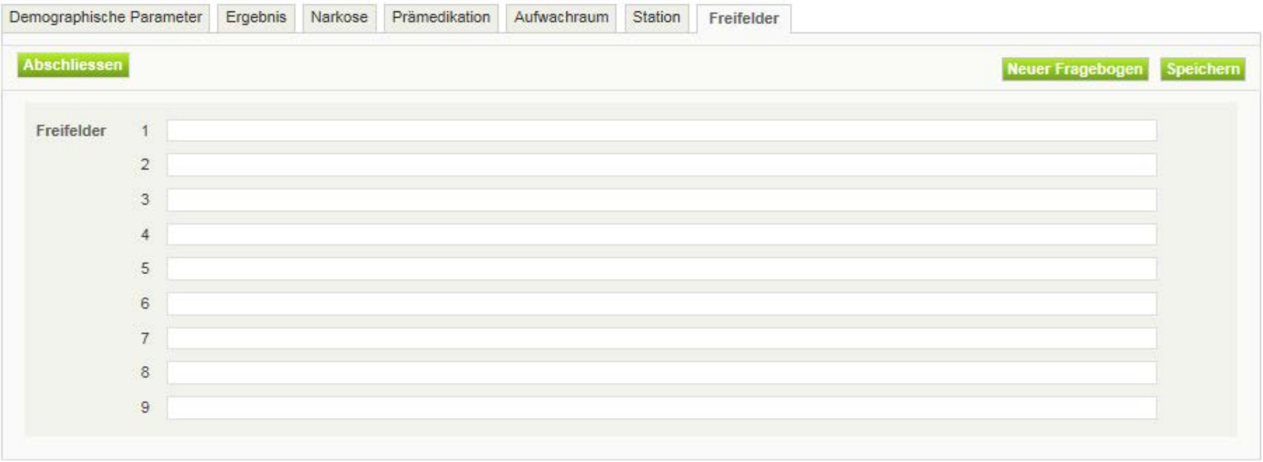

Abbildung 14: Fragebogen Freifelder

Haben Sie den ganzen Datensatz auf Vollständigkeit geprüft, gehen Sie auf den Button „Abschließen“, der sich am oberen rechten Rand befindet. Es öffnet sich daraufhin eine Dialogbox, in der Sie noch einmal gefragt werden, ob Sie den Fragebogen wirklich abschließen wollen, da der Datensatz im Anschluss daran nicht mehr editierbar ist.
Nun können Sie den nächsten Fragebogen aufrufen indem Sie den Button „Basismodul“ wählen und dann den Button „Neuer Fragebogen“.

Copyright Nutzung durch Dritte vorbehaltlich Genehmigung durch DGAI/ DGCH Version 2018

4.6 Häufige Fragen zur Datenerhebung

Wir möchten Ihnen anhand von einigen Beispielen die Dateneingabe weiter erläutern und auf häufig gestellte Fragen eingehen. Im Folgenden werden die Einträge erläutert, Medikamente in die jeweiligen Felder einzutragen, für eine vollständige Eingabe beachten Sie bitte auch die Möglichkeit den Button „keins gegeben“ auszuwählen.

Beispiel 1:

Der Patient Herr W. hat bereits eine chronische Schmerztherapie vor stationärer Aufnahme mit einem transdermalen System, Fentanyl-Pflaster 25 µg/h. Im Aufwachraum erhält er 1 g Novalgin als Kurzinfusion und 4 Boli à 3 mg Piritramid i.v. ohne die Verwendung einer PCA. Auf der Station bekommt er wieder 1 g Novalgin als Kurzinfusion und 1 g Novalgin als Tropfen (40 Tr.). Die Opioidgabe erfolgt aufgrund des erhöhten Schmerzmittelbedarfs nun mit Hilfe einer PCA, die zum Zeitpunkt der Befragung eine Menge von 7 Boli à 3 mg Piritramid als Verbrauch anzeigt.

Hinweis zur Dateneingabe der chronischen Schmerztherapie:

D 11 Chronischer Schmerz Opioid Hat der Patient vor seinem Krankenhausaufenthalt Opioide gegen chronische Schmerzen genommen?

(●) ja ( ) nein ( ) nicht erhebbar

Abbildung 15: chronische Schmerztherapie, Eintrag in der Karteikarte Demographie

Hinweis zur Dateneingabe im Aufwachraum:

A 1 Nicht-Opioid

☐ keins gegeben ☐ nicht erhebbar

	p.o.	i.v.	i.m.	Supp.
Celecoxib	☐			
Diclofenac	☐	☐	☐	☐
Etoricoxib	☐			
Gabapentin	☐			
Ibuprofen	☐	☐		☐
Metamizol	☐	☑	☐	☐
Naproxen	☐			
Paracetamol	☐	☐	☐	☐
Parecoxib		☐		
Pregabalin	☐			

A 2 Opioid

☐ keins gegeben ☐ nicht erhebbar

	p.o. unretard	p.o. retard	i.v.	i.m.	Supp.	s.c.
Buprenorphin	☐		☐	☐		☐
Fentanyl	☐		☐	☐		☐
Hydromorphon	☐	☐	☐	☐		☐
Morphin	☐	☐	☐	☐	☐	☐
Oxycodon	☐	☐	☐	☐		☐
Oxycodon/(Naloxon)*		☐				
Pethidin	☐		☐	☐	☐	☐
Piritramid			☑ 12 mg	☐		☐
Sufentanil	☐		☐	☐		☐
Tapentadol	☐	☐				
Tilidin/(Naloxon)*	☐	☐				
Tramadol	☐	☐	☐	☐	☐	☐

* Dosisangabe bezieht sich auf das Opioid

A 3 Ko-Analgetika

☑ keins gegeben ☐ nicht erhebbar

A 4 Mit PCA

( ) PCIA (intravenös) ( ) PCEA (epidural) ( ) PCRA (regional) ( ) PCA sublingual (●) keine erhalten ( ) nicht erhebbar

Abbildung16: Eintrag in der Karteikarte für den Zeitraum Aufwachraum

Copyright Nutzung durch Dritte vorbehaltlich Genehmigung durch DGAI/ DGCH Version 2018

Hinweis zur Dateneingabe auf der Station:

S 1 **Nicht-Opioid**

☐ keins gegeben ☐ nicht erhebbar

	p.o.	i.v.	i.m.	Supp.
Celecoxib	☐			
Diclofenac	☐	☐	☐	☐
Etoricoxib	☐			
Gabapentin	☐			
Ibuprofen	☐	☐		☐
Metamizol	☑	☑	☐	☐
Naproxen	☐			
Paracetamol	☐	☐	☐	☐
Parecoxib		☐		
Pregabalin	☐			

S 2 **Opioid**

☐ keins gegeben ☐ nicht erhebbar

	p.o. unretard	p.o. retard	i.v.	i.m.	Supp.	s.c.
Buprenorphin	☐		☐	☐		☐
Fentanyl	☐		☐	☐		☐
Hydromorphon	☐	☐	☐	☐		☐
Morphin	☐	☐	☐	☐	☐	☐
Oxycodon	☐	☐	☐	☐		☐
Oxycodon/(Naloxon)*		☐				
Pethidin	☐		☐	☐	☐	☐
Piritramid			☑ 21 mg	☐		☐
Sufentanil	☐		☐	☐		☐
Tapentadol	☐	☐				
Tilidin/(Naloxon)*	☐	☐				
Tramadol	☐	☐	☐	☐	☐	☐

* Dosisangabe bezieht sich auf das Opioid

S 3 **Ko-Analgetika**

☑ keins gegeben ☐ nicht erhebbar

S 4 **Mit PCA**

◉ PCIA (intravenös) ○ PCEA (epidural) ○ PCRA (regional) ○ PCA sublingual ○ keins gegeben ○ nicht erhebbar

Abbildung 17: Eintrag in der Karteikarte für den Zeitraum der Station

Beispiel 2:

Die Patientin Frau W. erhält zur Prämedikation eine Tablette Targin 10/5, postoperativ im Aufwachraum 1 Ampulle Dynastat i. v. verabreicht. Auf der Station erhält sie Ibuprofen 600 mg als Tablette, 1 g Novalgin als Tropfen und die Opioid - Medikation wird auf 2x1Tbl. Targin 10/5 festgelegt. Zum Befragungszeitpunkt hat die Patientin die zweite Dosis bereits erhalten.

Hinweis: **Die zu erhebende Dosis für die Station wird immer für den postoperativen Zeitraum auf der Station bis zum Zeitpunkt der Befragung erfasst.**

Copyright Nutzung durch Dritte vorbehaltlich Genehmigung durch DGAI/ DGCH Version 2018

Hinweis zur Dateneingabe zur Prämedikation:

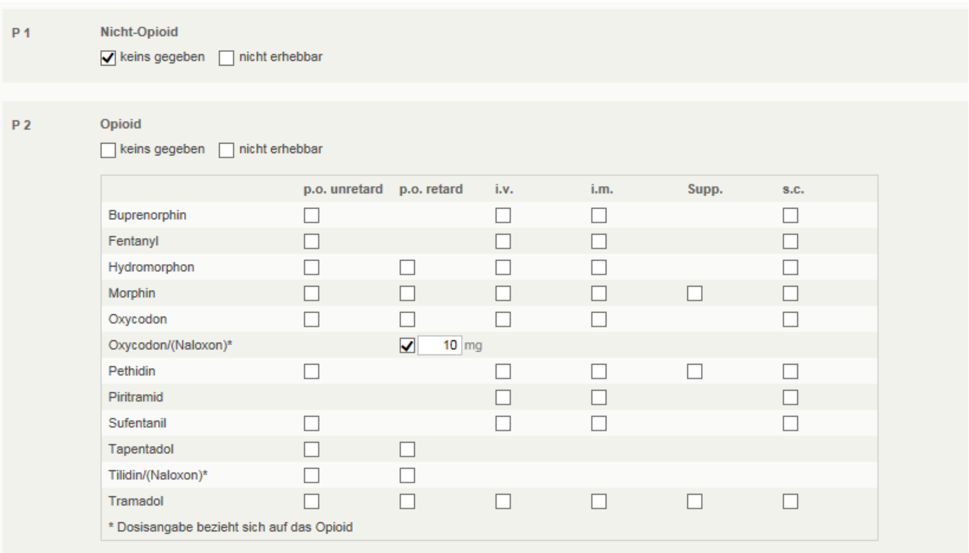

P 1 Nicht-Opioid

☑ keins gegeben ☐ nicht erhebbar

P 2 Opioid

☐ keins gegeben ☐ nicht erhebbar

	p.o. unretard	p.o. retard	i.v.	i.m.	Supp.	s.c.
Buprenorphin	☐		☐	☐		☐
Fentanyl	☐		☐	☐		☐
Hydromorphon	☐	☐	☐	☐		☐
Morphin	☐	☐	☐	☐	☐	☐
Oxycodon	☐	☐	☐	☐		☐
Oxycodon/(Naloxon)*		☑ 10 mg				
Pethidin	☐		☐	☐	☐	☐
Piritramid			☐	☐		☐
Sufentanil	☐		☐	☐		☐
Tapentadol	☐	☐				
Tilidin/(Naloxon)*	☐	☐				
Tramadol	☐	☐	☐	☐	☐	☐

* Dosisangabe bezieht sich auf das Opioid

Abbildung 18: Eintrag in der Karteikarte für die Prämedikation

Hinweis zur Dateneingabe für den Aufwachraum:

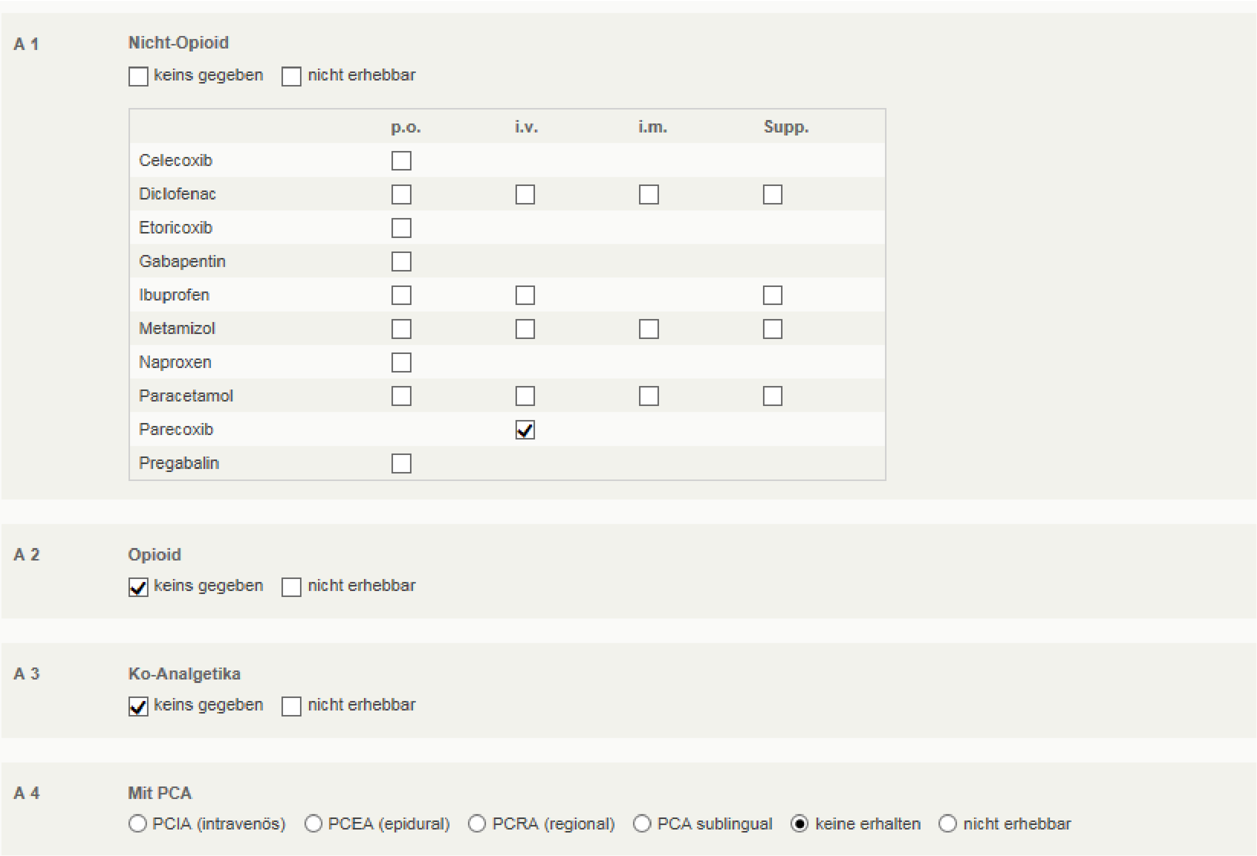

A 1 Nicht-Opioid

☐ keins gegeben ☐ nicht erhebbar

	p.o.	i.v.	i.m.	Supp.
Celecoxib	☐			
Diclofenac	☐	☐	☐	☐
Etoricoxib	☐			
Gabapentin	☐			
Ibuprofen	☐	☐		☐
Metamizol	☐	☐	☐	☐
Naproxen	☐			
Paracetamol	☐	☐	☐	☐
Parecoxib		☑		
Pregabalin	☐			

A 2 Opioid

☑ keins gegeben ☐ nicht erhebbar

A 3 Ko-Analgetika

☑ keins gegeben ☐ nicht erhebbar

A 4 Mit PCA

○ PCIA (intravenös) ○ PCEA (epidural) ○ PCRA (regional) ○ PCA sublingual ◉ keine erhalten ○ nicht erhebbar

Abbildung 19: Eintrag in der Karteikarte für den Zeitraum Aufwachraum

Copyright Nutzung durch Dritte vorbehaltlich Genehmigung durch DGAI/ DGCH Version 2018

Hinweis zur Dateneingabe für die Station:

S 1 **Nicht-Opioid**

☐ keins gegeben ☐ nicht erhebbar

	p.o.	i.v.	i.m.	Supp.
Celecoxib	☐			
Diclofenac	☐	☐	☐	☐
Etoricoxib	☐			
Gabapentin	☐			
Ibuprofen	☑	☐		☐
Metamizol	☑	☐	☐	☐
Naproxen	☐			
Paracetamol	☐	☐	☐	☐
Parecoxib		☐		
Pregabalin	☐			

S 2 **Opioid**

☐ keins gegeben ☐ nicht erhebbar

	p.o. unretard	p.o. retard	i.v.	i.m.	Supp.	s.c.
Buprenorphin	☐		☐	☐		☐
Fentanyl	☐		☐	☐		☐
Hydromorphon	☐	☐	☐	☐		☐
Morphin	☐	☐	☐	☐	☐	☐
Oxycodon	☐	☐	☐	☐		☐
Oxycodon/(Naloxon)*		☑ 20 mg				
Pethidin	☐		☐	☐	☐	☐
Piritramid			☐	☐		☐
Sufentanil	☐		☐	☐		☐
Tapentadol	☐	☐				
Tilidin/(Naloxon)*	☐	☐				
Tramadol	☐	☐	☐	☐	☐	☐

* Dosisangabe bezieht sich auf das Opioid

S 3 **Ko-Analgetika**

☑ keins gegeben ☐ nicht erhebbar

S 4 **Mit PCA**

◉ PCIA (intravenös) ○ PCEA (epidural) ○ PCRA (regional) ○ PCA sublingual ○ keins gegeben ○ nicht erhebbar

Abbildung 20: Eintrag in der Karteikarte für den Zeitraum Station

Beispiel 3:
Die Patientin Frau G. erhielt präoperativ einen Periduralkatheter, der intraoperativ mit 5 ml 0,25 % Bupivacain bestückt wurde. Zum Befragungszeitpunkt wurde der Katheter kontinuierlich aus einem Mischbeutel, der 0,15 % Ropivacain und 3 µg/ml Sufenta enthält, mit einer Laufrate von 10 ml/h bestückt.

Hinweis: **Die Opioid - Menge und die Lokalanästhetika, die zur Regionalanästhesie zum Einsatz kommen, werden nicht erfasst. Sollte es für die Auswertung Ihrer Schmerztherapie wichtig sein, nutzen Sie die Freifelder.**

Copyright Nutzung durch Dritte vorbehaltlich Genehmigung durch DGAI/ DGCH Version 2018

Hinweis zur Dateneingabe für die Narkose:

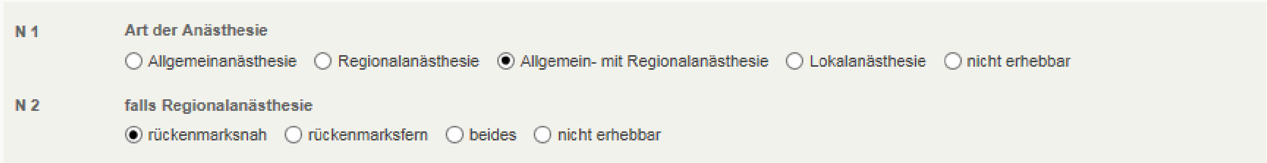

Abbildung 21: Eintrag in der Karteikarte für die Narkose

Hinweis zur Dateneingabe für die Station:

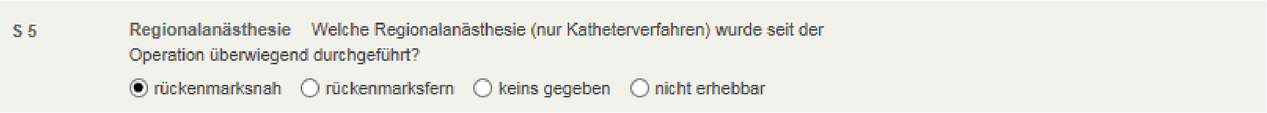

Abbildung 22: Eintrag in der Karteikarte für den Zeitraum der Station

Beispiel 4:
Kliniksinterne Fragestellung: Wo waren Ihre Schmerzen am stärksten?
Kodieren Sie sich die Antwortmöglichkeiten:
1 = Aufwachraum; 2 = Station; 3 = beides gleich; 4 = nicht erhebbar

Der Patient Herr K. gibt an, die stärksten Schmerzen habe er im Aufwachraum erlebt.

Hinweis zur Dateneingabe in den Freifeldern

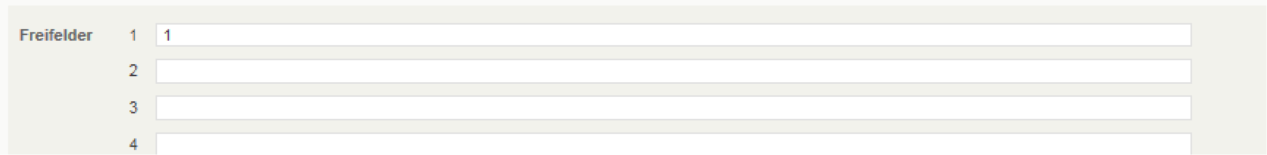

Abbildung 23: Eintrag für spezielle Fragestellungen in den Freifeldern

Haben Sie Fragen zur Dateneingabe, kontaktieren Sie uns einfach per Mail quips@med.uni-jena.de oder telefonisch unter 03641 9323354.
Sollten Sie Fragen zu Ihren Zugangsdaten oder zur Rückmeldung Ihrer Daten haben, wenden Sie sich bitte an den technischen Support der Firma TAKWA und schreiben Sie eine E-Mail an bms@takwa.de oder rufen Sie einfach unter der Telefonnummer 0361 6534096 an.

4.7 Sicherung der Datenqualität

Um mögliche Fehler in der Datenübertragung zum zentralen Server zu entdecken, werden regelmäßig Qualitätskontrollen und Plausibilitätsüberprüfungen durchgeführt.

Ein hohes Niveau an Datenqualität kann in erster Linie durch das frühzeitige Ausschalten von Fehlerquellen erreicht werden. Solche „Fehlerfilter“ sind an mehreren Stellen im Datenerfassungssystem eingebaut. Als Nutzer werden Sie bei der Eingabe der Daten feststellen, dass die Software bestimmte Grenzwerte vorgibt, in deren Rahmen Ihre eingegebenen Werte liegen müssen. Wo immer möglich, wird eine Auswahlliste angeboten, um Fehleingaben zu minimieren.

Copyright Nutzung durch Dritte vorbehaltlich Genehmigung durch DGAI/ DGCH Version 2018

5 Ergebnisrückmeldung

Das Feedback Ihrer Ergebnisqualitätsparameter können Sie auf der QUIPS-Website (www.quips-projekt.de) unter dem Menüpunkt Applikation „Fragebogen/Benchmarkserver“ abrufen. Voraussetzung für eine sinnvolle Datenauswertung und grafische Darstellung ist eine Mindestanzahl von 30, besser 50 eingegebenen Datensätzen pro auszuwertende Station.

Der Zugriff auf den Benchmarkserver ist durch ein Passwort geschützt. Dieses Passwort wird Ihnen nach dem Abschluss der Formalitäten (Vertragsabschluss zur Teilnahme am QUIPS-Projekt) durch die Firma TAKWA zugesandt.

5.1 Charakterisierung des Feedbacks

Nach der Eingabe des Passwortes stehen auf der linken Seite (siehe Abb. 23): folgende Auswahlmöglichkeiten und Benchmark-**Items** zur Auswahl:

- Allgemeine Dateninformationen
- Prozesse
- Ergebnisse zu Schmerzintensitäten, Beeinträchtigungen und Nebenwirkungen
- Prozessbeurteilung
- Datenexport

Sie können die Patientengruppe, die Sie betrachten (Ihre Stichprobe), mit Filtern nach Ihren Wünschen in der Mitte der Maske einschränken. Für die Darstellung der Ergebnisparameter (Feedback) einer Benchmarkgruppe (Fachrichtung) stehen folgende **Filter** und Auswahlmöglichkeiten zur Verfügung (siehe Abb. 23):

- Benchmarkgruppe
- Zeitraum
- Grafik-Typ
- der entsprechende „post-OP-Tag“
- Mindestanzahl der Datensätze für die angezeigten Stationen
- OPS-Code
- Art des Anästhesieverfahren
- Regionalverfahren auf der Station
- PONV Prophylaxe
- PCA-Therapie
- Alter
- Geschlecht

Durch das gezielte Setzen der Filter entsteht eine homogene Gruppe für die Beurteilung der Schmerztherapie und eine realistische Wertung des Rankings.

Copyright Nutzung durch Dritte vorbehaltlich Genehmigung durch DGAI/ DGCH Version 2018

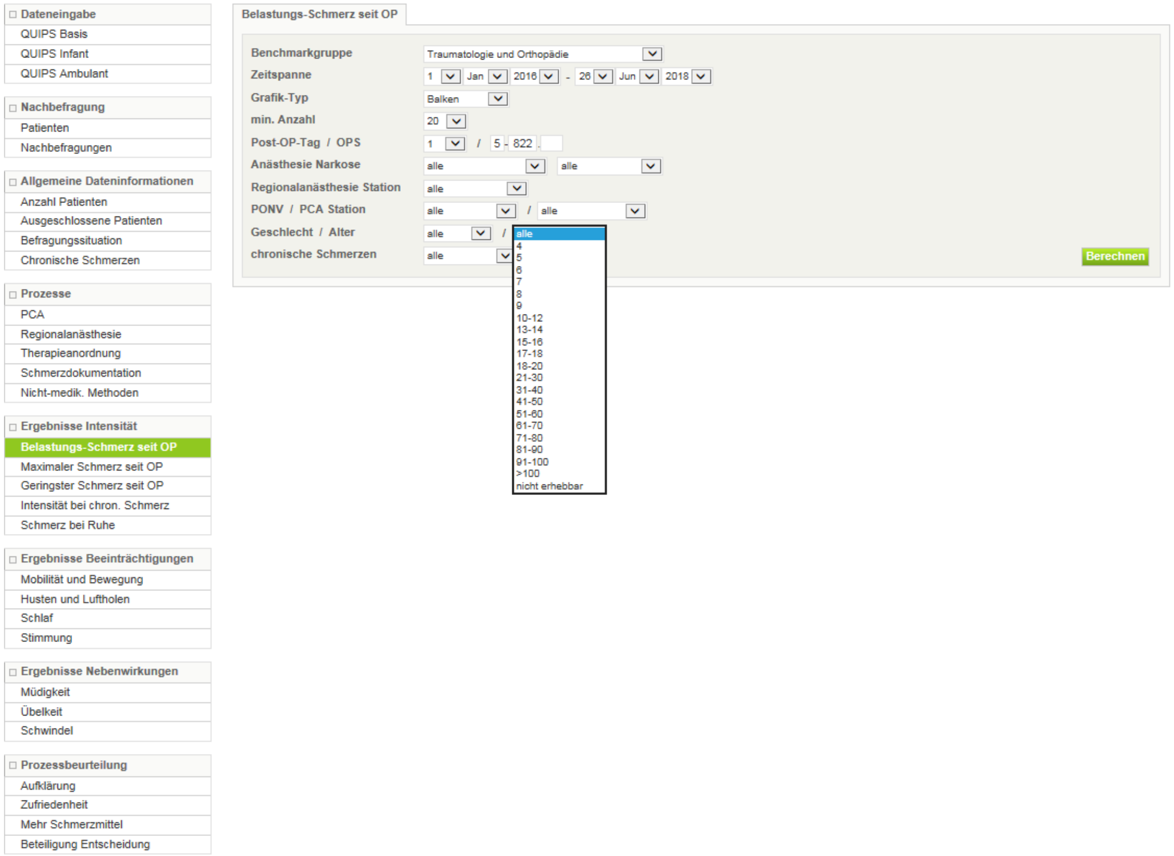

Abbildung 23: Ergebnisrückmeldung am **Item** *Schmerz bei Belastung* mit Setzen der **Filter** OPS-Codierung und Altersauswahl.

Unter der **„Benchmark-Gruppe"** wird die OP-Disziplin ausgewählt, für welche die Rückmeldung erfolgen soll. Gegenwärtig stehen als OP-Disziplinen: Allgemeinchirurgie, Gynäkologie, Geburtshilfe, Hals, -Nasen und Ohrenheilkunde, Thoraxchirurgie, Herzchirurgie, Traumatologie und Orthopädie, Urologie, Neurochirurgie, Wirbelsäulenchirurgie, Mund-, Kiefer- und Gesichtschirurgie/Plastische Chirurgie, Gefäßchirurgie, Augenheilkunde und „Sonstige Stationen" zur Auswahl.

Unter „Sonstige Stationen" sind bisher die Disziplinen aufgeführt, die noch keine ausreichende Teilnehmeranzahl aufweisen. Sobald die Kliniken ausreichend Stationen mit entsprechendem Fachbereich anmelden, wird die Benchmarkgruppe „Sonstige Stationen" aufgeteilt und die Disziplinen jeweils separat dargestellt.

Eine weitere Auswahl, vor allem um Operationen mit geringen Fallzahlen oder Vorkommen in mehreren Fachbereichen aufzufinden, ist „sämtliche Kliniken" auszuwählen. Hier kann man den Operationscode eingeben und „suchen" anklicken. So finden Sie zum Beispiel die Daten nach einer „Bandscheibenoperation" in den Benchmarkgruppen Wirbelsäulenchirurgie, Neurochirurgie oder Orthopädie.

Hinweis: Bei einigen Benchmarkgruppen, z.B. in der Allgemeinchirurgie nehmen deutschlandweit sehr viele Stationen teil. Für eine bessere Übersicht wählen Sie den Button „Drucken" (erscheint neben Berechnen). Sie bekommen eine Darstellung der eigenen Station gegenüber den fremden Teilnehmern mit einer gleichmäßigen Gesamt- Verteilung. Dies hat den Vorteil, dass der Gesamt-Eindruck der Verteilung erhalten bleibt und die eigenen Balken besser eingeordnet werden.

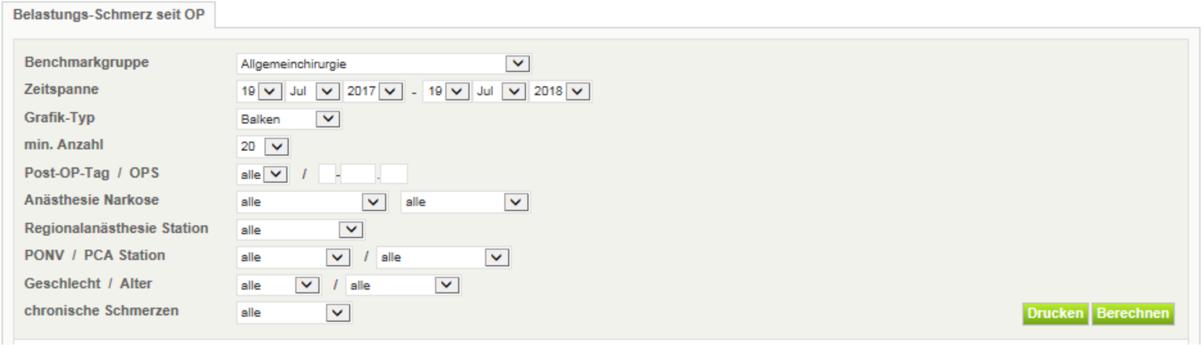

Abbildung 24: Druckversion

Copyright Nutzung durch Dritte vorbehaltlich Genehmigung durch DGAI/ DGCH Version 2018

Der **„Grafik-Typ“** bietet Ihnen drei Varianten an:

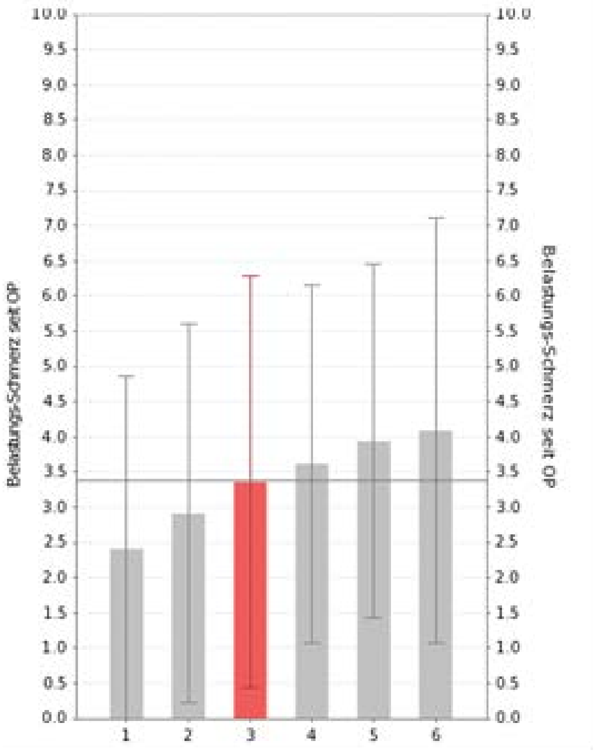

„Balken“: Auswahl der Ergebniswerte im gewählten Zeitintervall in absoluten Zahlen oder arithmetischen Mittelwerten in Form eines Balkendiagramms. Für die eigenen Stationen werden rote Balken mit Stationskennung verwendet, Die anderen Kliniken werden anonymisiert in grauer Farbe abgebildet (siehe Abb. 25)

Unterhalb der Graphik befindet sich eine Tabelle mit der Anzahl der Datensätze, dem arithmetischen Mittelwerten sowie der Standardabweichung.

Legende:
roter Balken: eigene Station
grauer Balken: fremde Station (anonymisiert)

Abbildung 25: Ergebnisrückmeldung Balkendiagramm am Beispiel Schmerz bei Belastung

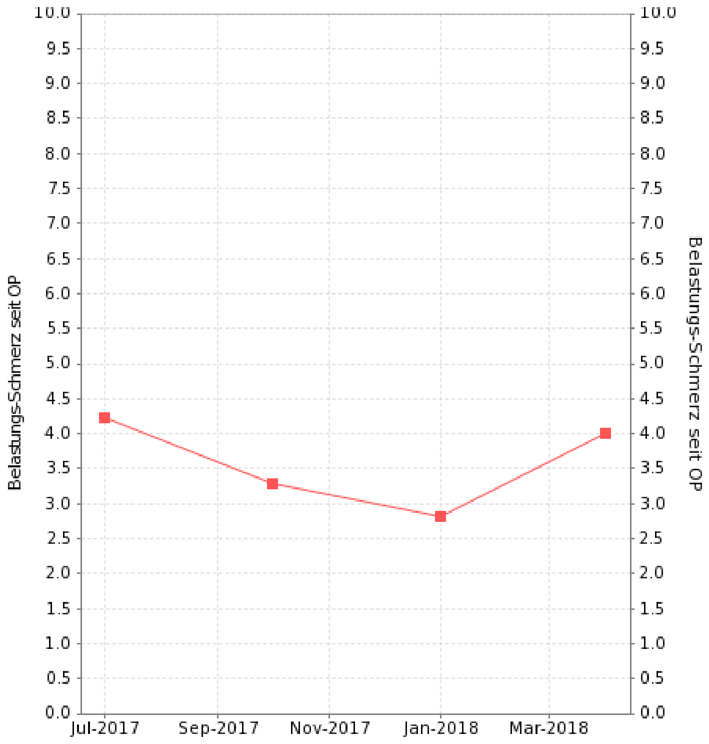

„Zeit“: Hier kann der zeitliche Verlauf, zum Beispiel die Schmerzintensität in einem Jahr als Ergebnisparameter eingesehen werden. Die eigenen Stationen sind als farbige Linie gekennzeichnet (siehe Abb.26). Der Median und der Mittelwert für die jeweiligen Stationen werden unterhalb der Grafik angegeben Sie können wählen, ob sie Quartalsweise, halbjährlich usw. die Datenpunkte haben möchten.

Abbildung 26: Ergebnisrückmeldung im Zeitverlauf am Parameter Schmerz bei Belastung

Copyright Nutzung durch Dritte vorbehaltlich Genehmigung durch DGAI/ DGCH Version 2018

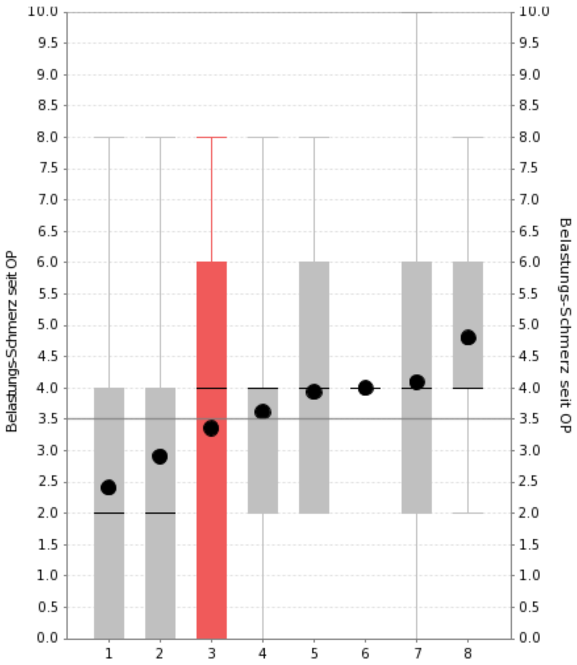

„Box-Whisker“: Es erfolgt die Darstellung der Ergebniswerte im gewählten Zeitintervall in absoluten Zahlen, arithmetischen Mittelwerten (Punkt) und Medianwerten (Strich) in Form eines Box-Whisker-Plots mit Angabe der Streuung (siehe Abb. 27). Für die eigenen Stationen werden rote Box-Plots mit Stationskennung verwendet, die anonymisierten Teilnehmer grau dargestellt.
Unterhalb der Graphik werden zusätzlich neben der Anzahl, dem arithmetischen Mittelwerten und Standardabweichungen auch die Quantile dargestellt.

Abbildung 27: Box- Whisker- Plots für den Parameter *Schmerz bei Belastung*

Beim Menüpunkt **„Zeitspanne“** kann der Zeitraum, für den das Feedback erfolgen soll, eingegeben werden. Es sind der Beginn des ausgewählten Zeitraums und das Ende separat einzugeben.
Bei dem Auswahlmenü **„Post-OP-Tag“** kann von Tag 1 bis 4 unterschieden werden.

Hinweis: Da die Grafiken jeweils aus den neuesten Daten aktuell erstellt werden, kann die Generierung der Ergebnisse manchmal ein paar Sekunden dauern.

Zusätzlich sind auf dem Benchmarkserver Filter aus den Prozessdaten gesetzt (Abb. 23). Man kann somit die Ergebnisse der Schmerztherapie aus Patientensicht weiter untersuchen, eventuell Defizite erkennen.
Exemplarisch für den Einsatz der Filter anhand der OPS-Codierung kann man die laparoskopische Cholezystektomie einsetzen. Der OPS-Code lautet **5-511.11**, weitere Filter sind die Auswahl des postoperativen Tages:**1,** die Narkoseart: **Allgemeinnarkose** und die **Mindestanzahl** der Datensätze der beteiligten Stationen soll mindestens 5 betragen (siehe Abb.28). Die graue horizontale Linie entspricht dem Mittelwert.
Nutzen Sie die Filter! Sie erreichen auf diesem Weg eine homogene Gruppe.
Hinweis: Setzen sie immer den Filter auf den postoperativen Tag, sodass Sie sich auch nur mit den postoperativen Verläufen vergleichen, an denen auch Sie ihre Patienten befragen.

Copyright Nutzung durch Dritte vorbehaltlich Genehmigung durch DGAI/ DGCH Version 2018

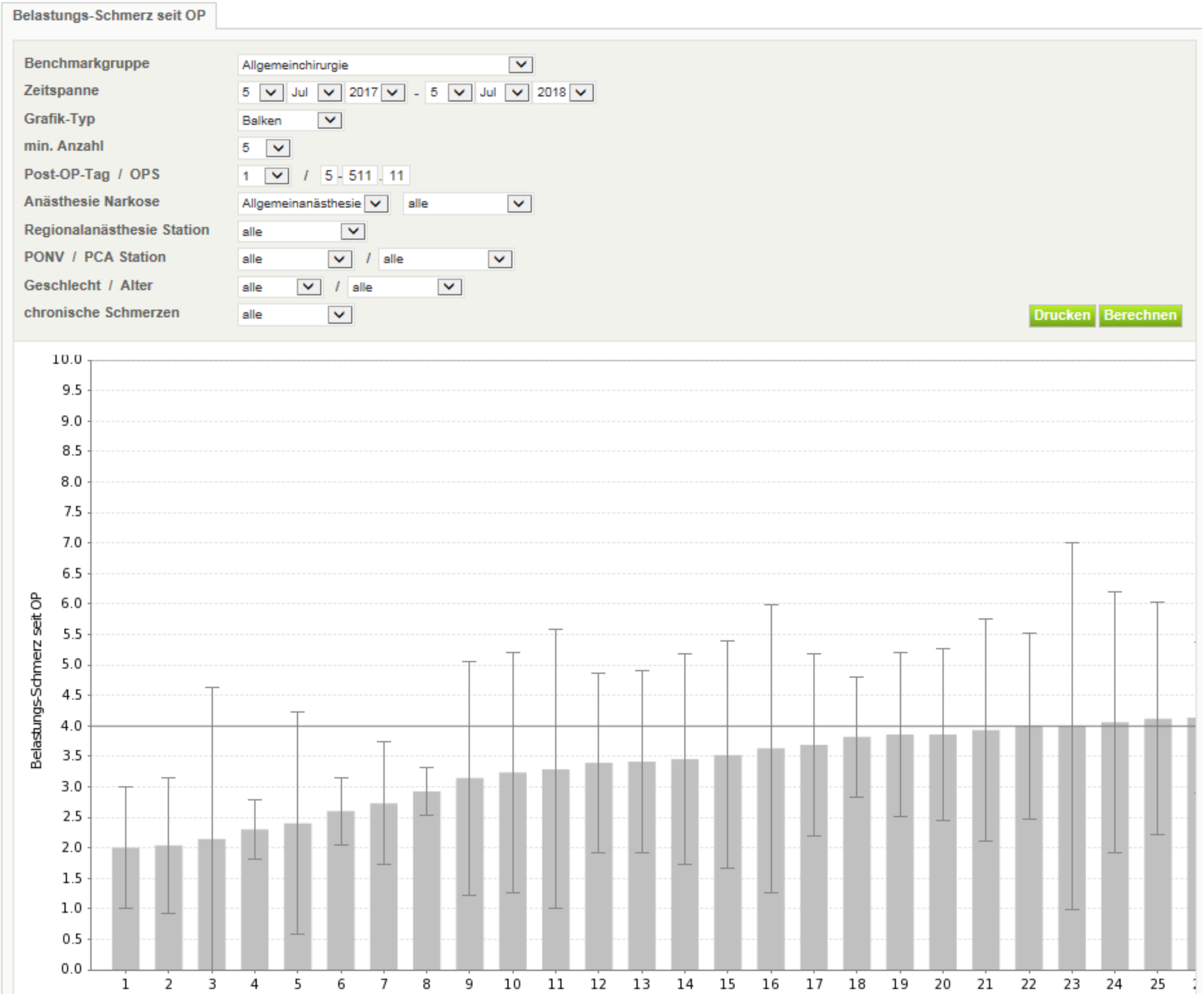

Abbildung 28: Eingriffsspezifische Darstellung bei dem Parameter *Belastungsschmerz nach laparoskopische Cholezystektomie*

5.2 Datenbestand

Unter **„Datenbestand"** kann die Anzahl der dokumentierten Patienten, sowie die Anzahl der ausgeschlossenen Patienten eingesehen werden. Nach Auswahl der OP-Disziplin können der Grafiktyp und der Zeitraum gewählt werden.

- Bei der Auswahl des Grafiktypen **„Balken"** und der Benchmark-Gruppe wird die Patientenanzahl zum aktuellen Zeitpunkt in absoluten Zahlen und in Form eines Balkendiagramms angezeigt. Für die eigenen Stationen werden rote Balken mit Stationskennung verwendet, die anderen Kliniken werden anonymisiert in grauer Farbe abgebildet.
- Bei der Auswahl des Grafiktypen **„Zeit"** kann der Verlauf der absoluten Patientenanzahl pro Monat eingesehen werden. Die eigenen Stationen sind als farbige Linie und mit Stationskennung gekennzeichnet.

Die Anzahl der nicht eingeschlossenen Patienten werden mit entsprechendem Ausschlussgrund (laut D 10 im Fragebogen) in der **„Balken"** Graphik angezeigt. Im zeitlichen Verlauf ist nur die Anzahl der ausgeschlossenen Datensätze zu sehen.

Copyright Nutzung durch Dritte vorbehaltlich Genehmigung durch DGAI/ DGCH Version 2018

5.3 Outcome- Parameter Schmerzintensität

Unter dem Menüpunkt “**Ergebnisse Intensität**“ sind folgende Items aufgeführt:

- **„Schmerz bei Belastung“**
- **„Maximaler Schmerz** seit Operation**“** und
- **„Geringster Schmerz** seit Operation**“** abgerufen werden.

Die Darstellung für die drei Outcome-Parameter erfolgt identisch. Als Beispiel wird hier die Rückmeldung für „Schmerz bei Belastung“ beschrieben (siehe Abbildung 25-27).

Der Erwartungswert (= Durchschnittswert) über alle Patienten aller beteiligten Zentren ist als horizontale graue Linie dargestellt. Die Länge der vertikalen Linie stellt den Standardfehler für die Punktschätzung des Mittelwertes dar.
Für die eigenen Stationen (rote Balken) werden jeweils unterhalb der Grafik die Stichprobengröße der Datensätze und der Mittelwert angegeben. Für eine sinnvolle Berechnung der statistischen Größen sollten mehr als 30 Datensätze pro Station und Zeitraum vorliegen.

Der Grafiktyp Zeit stellt die Entwicklung der Ergebnisse im gewählten Zeitintervall als Linie (Zeitverlauf) dar. Wird der Grafiktyp Box-Whisker genutzt, werden die Ergebnisparameter im gewählten Zeitintervall in absoluten Zahlen, Mittelwerten (Punkt) und Medianwerten (Strich) in Form eines Box-Plots dargestellt.

5.4 Outcome-Parameter Beeinträchtigungen aufgrund der Schmerzen

Unter diesem Menüpunkt “**Ergebnisse Beeinträchtigungen**“ sind folgende Items:

- **Mobilität und Bewegung**
- **Husten und Luftholen**
- **Schlaf**
- **Stimmung**

Für diese Parameter werden die Ergebnisse in identischer Form dargestellt, daher wird an dieser Stelle nur exemplarisch das Feedback zu „Husten und Luftholen“ erläutert. Die eigenen Stationen werden gegenüber den fremden Teilnehmern farblich hervorgerufen.

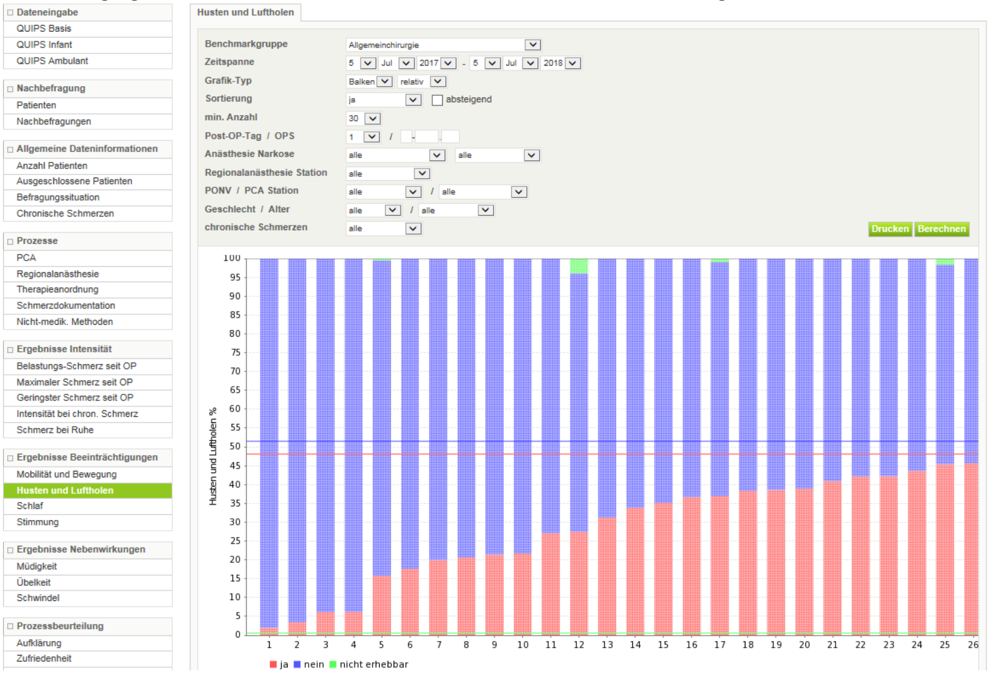

Abbildung 29: Beeinträchtigung beim Husten und Luftholen aufgrund der Schmerzen, Darstellung der Stationen mit allen Ergebnissen

Copyright Nutzung durch Dritte vorbehaltlich Genehmigung durch DGAI/ DGCH Version 2018

Legende:

ja — Anteil der Patienten, die aufgrund der Schmerzen beeinträchtigt sind.

nein — Anteil der Patienten, die aufgrund der Schmerzen nicht beeinträchtigt sind.

nicht erhebbar — Anteil der Patienten, die sich zu dieser Frage nicht geäußert haben.

Die rote durchgezogene Linie entspricht dem Mittelwert des Anteils der beeinträchtigen Patienten in Prozent, die blaue durchgezogene Linie entspricht dem Mittelwert ohne Beeinträchtigungen.

Unter diesem Menüpunkt “**Ergebnisse Nebenwirkungen**“ sind folgende Items:

- **Müdigkeit**
- **Übelkeit**
- **Schwindel**

Die Graphiken entsprechen den vorangegangenen Beispielen.

5.5 Prozessparameter

Unter diesem Menüpunkt “**Prozesse**“ sind folgende Items:

- **PCA**
- **Regionalanästhesie**
- **Therapieanordnung**
- **Schmerzdokumentation**
- **nicht- medikamentöse Methoden**

Die Darstellung ist identisch und wird daher beispielhaft für den Parameter „Schmerzdokumentation“ erläutert. Die eigenen Stationen werden gegenüber den fremden Teilnehmern farblich hervorgerufen.

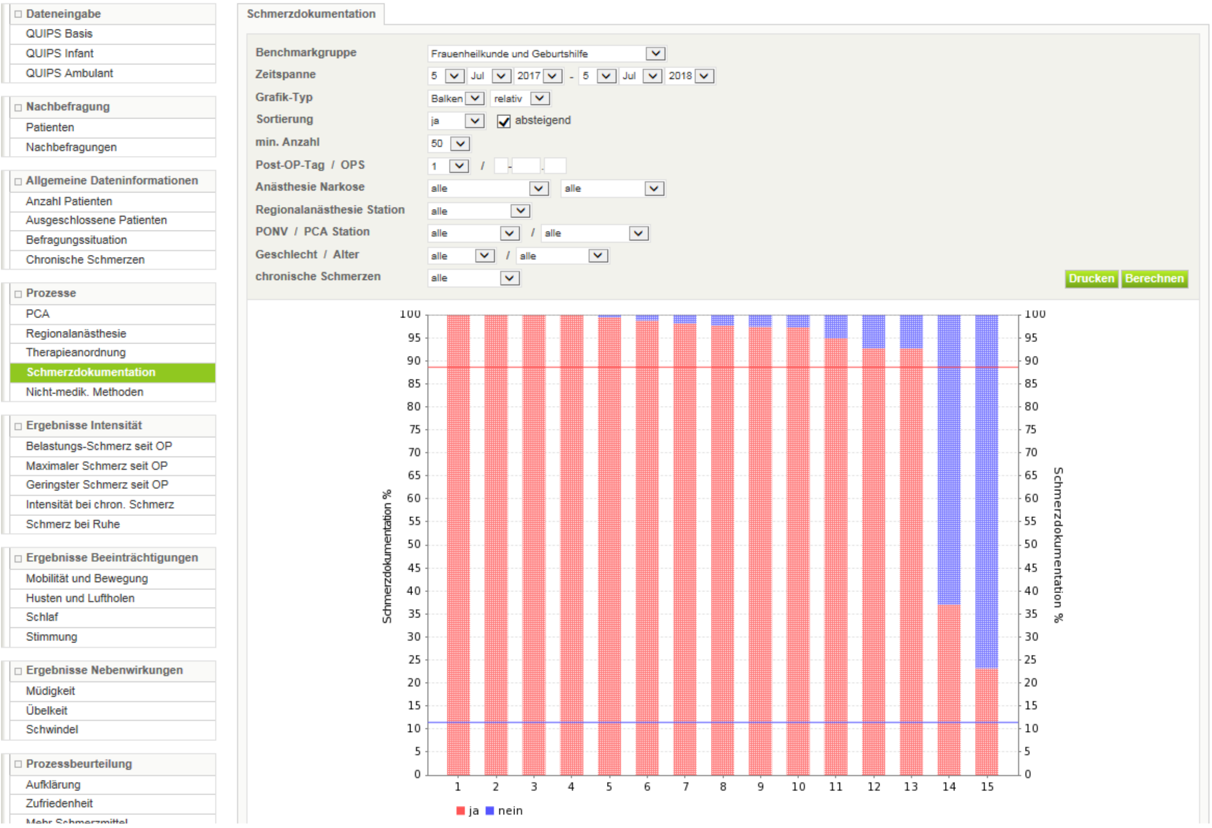

Abbildung 30a : Prozessparameter Schmerzdokumentation, Darstellung der Stationen mit allen Ergebnissen

Copyright Nutzung durch Dritte vorbehaltlich Genehmigung durch DGAI/ DGCH Version 2018

Legende:

ja Schmerzdokumentation ist vorhanden

nein Schmerzdokumentation ist nicht erfolgt

Die rote durchgezogene Linie entspricht dem Mittelwert des Anteils vorhandener Schmerzdokumentation in Prozent, die blaue durchgezogene Linie entspricht dem Mittelwert für fehlende Schmerzdokumentation.

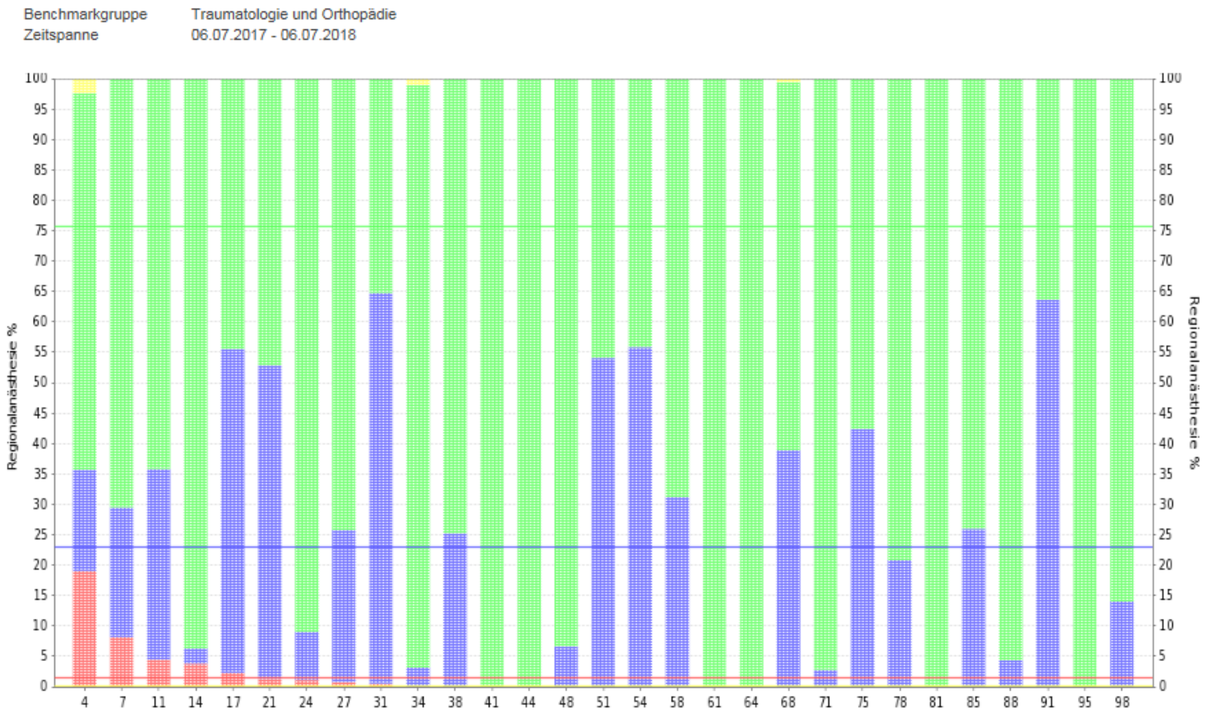

Abb.30b: Prozessparameter Regionalanästhesie auf Station, Darstellung Druckversion

Rückenmarksnah Anteil der rückenmarksnahen Regionalanästhesie in Prozent

Peripher Anteil der peripheren Regionalanästhesie in Prozent

keine Keine Regionalanästhesie in Prozent

nicht erhebbar Anteil der nicht erhebbaren Datensätze

Die durchgezogenen farbigen Linien entsprechen dem Mittelwert des Anteils der jeweiligen Regionalanästhesieverfahren.
Unter diesem Menüpunkt " **"Prozessbeurteilung"** sind folgende Items:

- **Aufklärung**
- **Zufriedenheit**
- **Mehr Schmerzmittel**
- **Beteiligung an der Entscheidung**

Die Graphiken entsprechen den vorangegangenen Beispielen.

Copyright Nutzung durch Dritte vorbehaltlich Genehmigung durch DGAI/ DGCH Version 2018

Ein Tipp bei der Beurteilung Ihrer Daten auf dem Benchmarkserver. Schauen Sie im Ranking der Stationen nicht nach einzelnen Platzierungen! Unterteilen Sie sich die teilnehmenden Stationen in den vorderen, mittleren und hinteren Teil und setzen sich ein Ziel, in welchem Drittel Sie sich wiederfinden möchten. Auch sehen Sie immer nach dem deutschlandweiten Durchschnitt, als diagonale Linie in der Graphik dargestellt.

	Durchschnitt	Wert der eigenen Station	Drittel: 1 /2 /3	Handlungs - bedarf
**Ergebnisse Intensität**				
Belastungs-Schmerz seit OP	3,9	2,1	1	nein
Maximaler Schmerz seit OP	5,2	2,9	1	nein
Geringster Schmerz seit OP	1,5	0,1	1	nein
Intensität bei chron. Schmerz				
**Ergebnisse Beeinträchtigungen**				
Mobilität und Bewegung	ja: 67%	ja: 26%	1	nein
Husten und Luftholen	ja: 7%	ja: 0%	1	nein
Schlaf	ja: 39%	ja: 10%	1	nein
Stimmung	ja: 17%	ja: 5%	1	nein
**Ergebnisse Nebenwirkungen**				
Müdigkeit	ja: 37%	ja: 53%	3	ja
Übelkeit	ja: 17%	ja: 36%	3	ja
Schwindel	ja: 21%	ja: 23%	2	später
**Prozessbeurteilung**				
Aufklärung	ja: 82%	ja: 43 %	3	ja
Zufriedenheit	8,4	9,5	1	nein
Mehr Schmerzmittel	ja: 12%	ja: 7 %	1	nein
Beteiligung Entscheidung	7,6	8,9	1	nein

Auswertungsbeispiel mit der Graphik via Benchmakserver

5.6 Rohdaten

Unter Daten -> Export können Sie sich die Rohdaten Ihrer Klinik herunterladen. Stellen Sie dazu den gewünschten Datei-Typ (CSV, Excel, SPSS) und die Zeitspanne ein. Klicken Sie dann auf den Button „Download“. Ihre Daten werden erzeugt. Danach können Sie die Ihre Daten mittels des Buttons „Speichern“ auf Ihren Rechner laden.

Auf derselben Seite können Sie zusätzlich die Kodierung der Werte herunterladen. Dort wird erklärt, unter welchem Variablennamen sich welche Frage aus dem Fragebogen versteckt. Zusätzlich werden dort die gespeicherten Werte erklärt. Falls Sie Fragen bzgl. des Namens oder des Wertes einer Variablen haben, schauen Sie bitte in diese Datei.

Copyright Nutzung durch Dritte vorbehaltlich Genehmigung durch DGAI/ DGCH Version 2018

6 Feedback-Meeting

Der wichtigste und zugleich schwierigste Aspekt des QUIPS-Projektes ist die Entwicklung und Umsetzung neuer Strategien zur Verbesserung Ihrer Schmerztherapie. Feedback zur Ergebnisqualität führt erst dann zu einer Verbesserung der Therapie, wenn aus den rückgemeldeten Daten auch entsprechende Konsequenzen gezogen werden.

Zur Interpretation der Ergebnisse hat es sich als äußerst hilfreich erwiesen, auch die Prozessdaten zu erheben. Dadurch wird die Datenerfassung zwar aufwendiger, der Benefit aus den Daten ist jedoch auch ungleich größer. Wir werden Ihnen in Form eines Newsletters und in moderierten Anwendertreffen Interpretationshilfen und mögliche Konsequenzen an verschiedenen Beispielen zur Verfügung stellen.
Auch bieten wir Ihnen Workshops in weiterführender Statistik an.

Sinnvoll ist schon im Vorfeld des Projektes die Einrichtung einer interdisziplinären und interprofessionellen Arbeitsgruppe (Anästhesie, Chirurgie, Physiotherapie, Ärzte, Pflege, evtl. Apotheke, Verwaltung, Qualitätsmanagement), die in regelmäßigen Abständen (z. B. alle 3 Monate) die Ergebnisse bespricht. Wichtig ist, dass die Mitglieder in klinikinterne Entscheidungen involviert sind und in der Lage sind, in der Einrichtung, die sie vertreten, eine Multiplikatorfunktion zu übernehmen.

Auf Wunsch vermitteln wir auch die Möglichkeit zur Hospitation bei erfolgreichen Kliniken.

Copyright Nutzung durch Dritte vorbehaltlich Genehmigung durch DGAI/ DGCH Version 2018

6.1 Überblick Changemanagement

Stets gilt es zu bedenken, dass nichts schwieriger zu bewerkstelligen, nichts von zweifelhafteren Erfolgsaussichten begleitet und nichts gefährlicher zu handhaben ist als eine Neuordnung der Dinge. (Niccolo Macchiavelli)

Dabei sieht es doch so einfach aus...

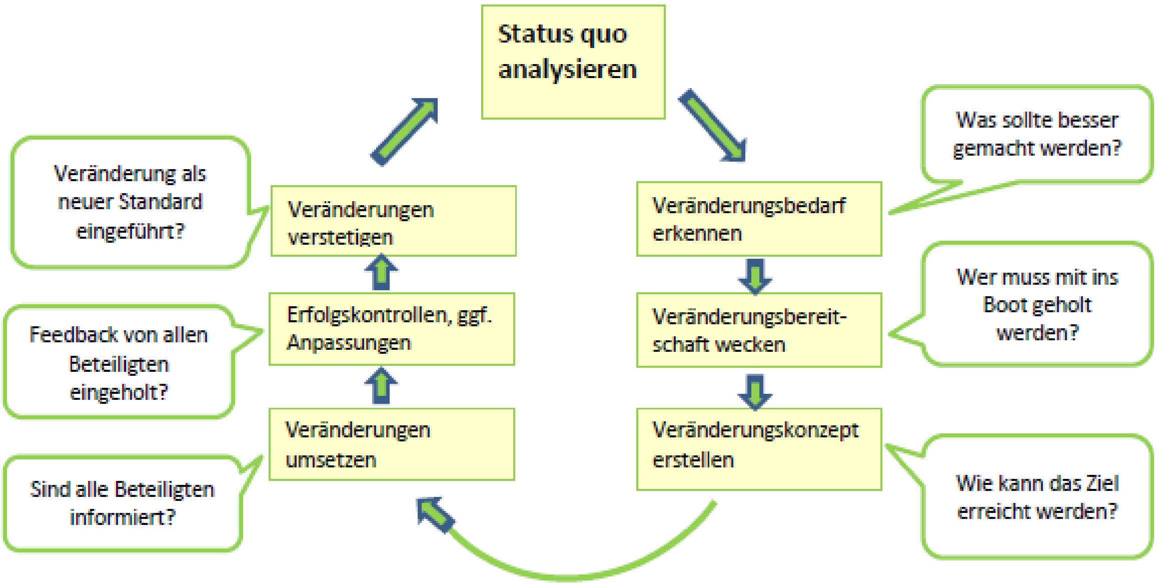

Vorsicht Stolperfallen!

- Mangelndes Problembewusstsein bei Kollegen und/oder Vorgesetzten („Das haben wir doch schon immer so gemacht!“)
- Mangelndes Verständnis
- Mangelhafte Kommunikation

Wahrnehmungsprobleme

- Angst vor Veränderungen
- Angst Fehler zu machen
- Beteiligte fühlen sich kritisiert

Emotionale Hindernisse

- Fehlende Diskussions- und Kritikkultur
- Starre Hierarchien
- Mangelnde Unterstützung

Strukturelle Hindernisse

Deshalb ...

- Alle Beteiligten einbeziehen; als Partner gewinnen
- Ziele, Vorgehensweise, Erfolgskontrollen offen und deutlich kommunizieren und dokumentieren
- Herausstellen, welche Vorteile die Veränderungen im Idealfall bringen
- Wertschätzender Umgang miteinander!

Überblick Change-Management CW 01.06.16

Copyright Nutzung durch Dritte vorbehaltlich Genehmigung durch DGAI/ DGCH Version 2018

7 Zusammenfassung/Überblick

Hier sehen sie noch einmal kurz zusammengefast, welche Schritte im Einzelnen nötig sind, um mit dem Projekt zu starten.

Checkliste

- ✓ Schulung erhalten und Quiz erfolgreich ausgefüllt
- ✓ Vertrag unterschrieben
- ✓ Strukturfragebogen für das Krankenhaus und die teilnehmende Stationen ausgefüllt und an TAKWA geschickt
- ✓ Ethikvotum vorhanden
- ✓ Geschultes Personal und Rechnerarbeitsplatz mit Internet vorhanden
- ✓ Kooperation der chirurgischen Stationen sichergestellt

Los geht’s mit QUIPSEN!

Hilfreiche Kontakte:

info@takwa.de Sie wollen neue Stationen anlegen oder haben Fragen zur Software.

quips@med.uni-jena.de Wenn Sie Fragen rund ums Projekt haben, z.B. Dateneingabe, zu Fragebögen, weitern Modulen (QUIPSInfant, QUIPS ambulant, QUIPS konservativ) etc.

www.quips-projekt.de: immer einen Blick wert! Veranstaltungshinweise, Workshops, News, Best-of-Reports

www.pain-out.eu: zum Blick über den deutschen Tellerrand hinaus! Was passiert im postoperativen Schmerzmanagement weltweit?!

Copyright Nutzung durch Dritte vorbehaltlich Genehmigung durch DGAI/ DGCH Version 2018

8 Zusätzliche Angebote aus dem PAIN OUT Projekt

8.1 Elektronische Wissensbibliothek (EKL)

Da viele der in den letzten Jahren entstandenen Behandlungsleitlinien zum Thema postoperativer Schmerz nur schwer zugänglich, bzw. sehr umfangreich sind, wurden im Rahmen des PAIN OUT Projekts für zahlreiche patienten-, situations- und therapiespezifische Themen kurze und prägnante Zusammenfassungen erstellt.
Die Originalquellen (Leitlinien der American Pain Society, des Australischen und Neuseeländischen College of Anaesthetists, der Deutschen interdisziplinären Vereinigung Schmerztherapie, der PROSPECT-Gruppe, der Bandolier-Gruppe und der französischen Societé d'Anesthésiologie) sind dabei per Mausklick aufrufbar.

Die EKL ist vor allem für Kliniker (Chirurgen, Anästhesisten), aber auch für den Akutschmerzdienst, für den Pflegedienst und für Physiotherapeuten eine nützliche Informationsquelle. Sie umfasst die Aspekte

- allgemeine Aspekte in der Behandlung postoperativer Schmerzen (general aspects in management of postoperative pain)
- Therapiespezifische Schmerztherapie (treatment specific management of postoperative pain)
- Patienten- und situationsspezifische Schmerztherapie (patient or condition specific management of postoperative pain) und
- Eingriffsspezifische Schmerztherapie (procedure specific management of postoperative pain)

Da PAIN OUT ein internationales Projekt ist, sind alle Texte der EKL auf Englisch.

Um die EKL nutzen zu können, erhalten QUIPS Teilnehmer einen Login für die PAIN OUT Projektwebsite www.pain-out.eu

Ein kleiner Einblick in die EKL:

1. Einloggen auf www.pain-out.eu:

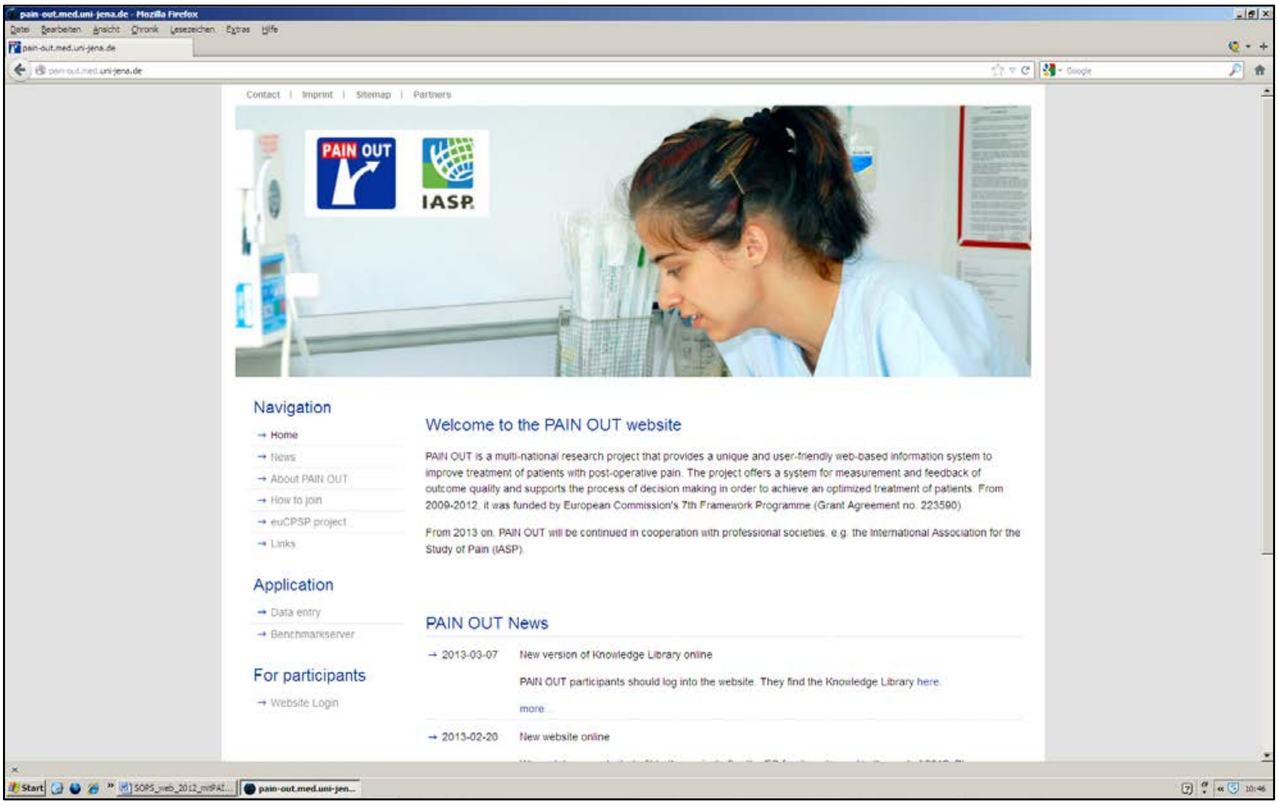

2. Im Menü die ‚Knowledge Library' auswählen:

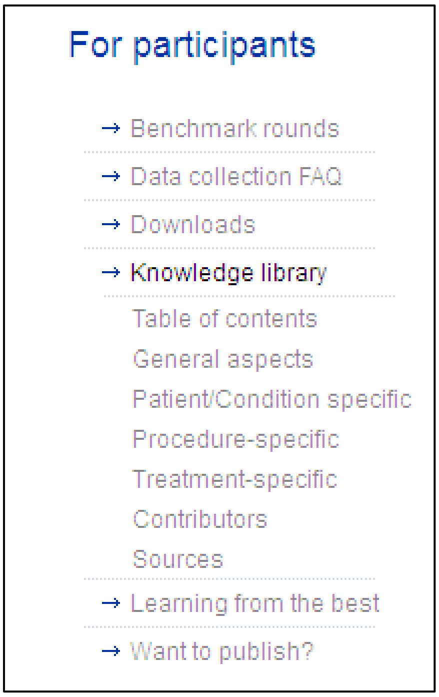

3. Gewünschtes Kapitel auswählen:

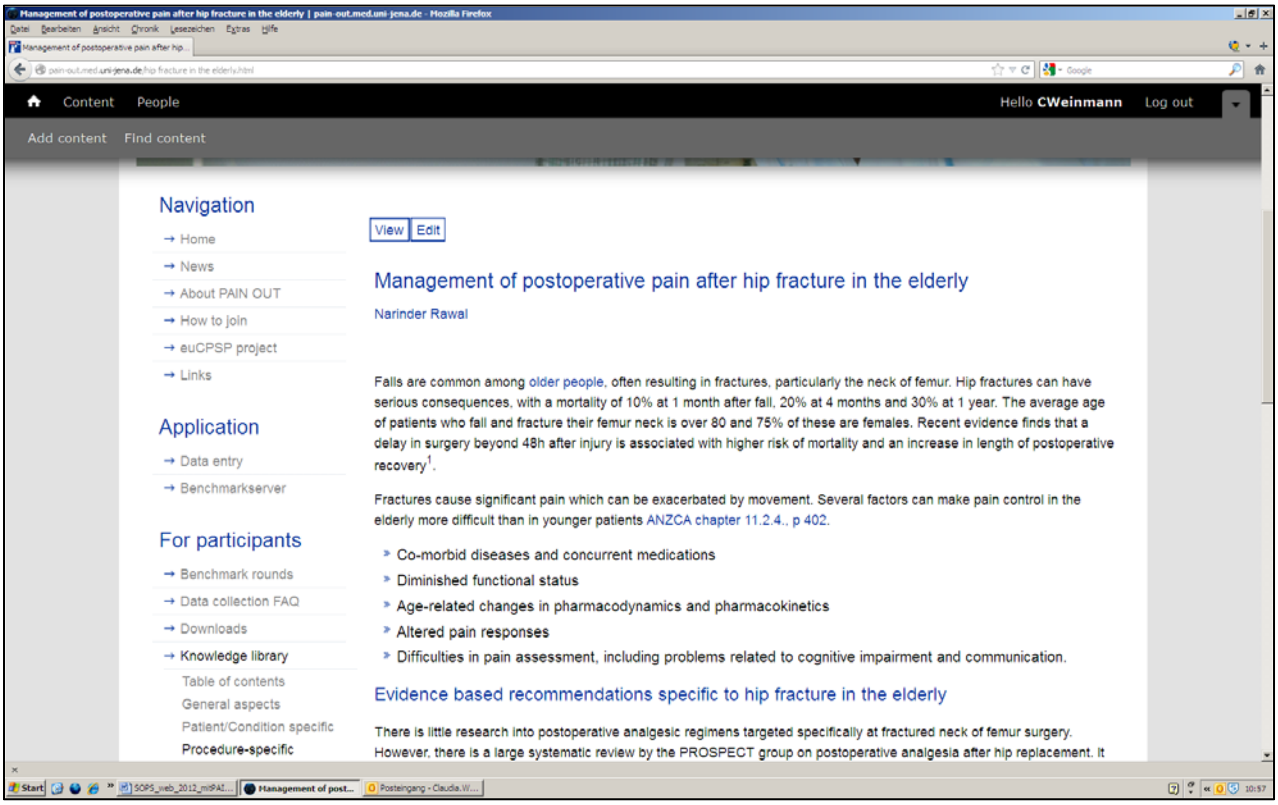

Copyright Nutzung durch Dritte vorbehaltlich Genehmigung durch DGAI/ DGCH Version 2018

8.2 Teilnahme an PAIN OUT (Improvement in postoperative PAIN OUTcome)

Falls Sie sich international vergleichen möchten, haben Sie als QUIPS Teilnehmer die Möglichkeit, kostenlos am PAIN OUT Projekt teilzunehmen.
Auf www.pain-out.eu finden Sie unter dem Menüpunkt ‚How to join' alle notwendigen Informationen dazu.

9 Zu guter Letzt ...

Wir hoffen, dass wir Ihnen mit diesen SOPs alle Informationen an die Hand gegeben haben, die Sie für eine erfolgreiche Durchführung des QUIPS Projektes benötigen. Da im weiteren Verlauf unseres Projektes immer wieder Neuerungen und Veränderungen eingearbeitet werden, stellen wir für Sie immer die aktuellste Version der SOPs und des Fragebogens in unserem Downloadbereich auf der Website www.quips-projekt.de zur Verfügung.
Gern möchten wir Sie auch motivieren, mit Ihren eigenen Daten oder der deutschlandweiten Datenbank wissenschaftlich zu arbeiten. Nach einem formlosen Antrag an die Geschäftsstelle der DGAI und einer Prüfung durch uns, ermöglichen wir es Ihnen gern Sollten Sie noch weitere Fragen haben, können Sie sich gerne mit einer E-Mail an uns wenden (quips@med.uni-jena.de). Wir werden unser Möglichstes tun, um Ihnen mit Ihrem Anliegen weiterzuhelfen.

Unser Team wünscht Ihnen viel Erfolg
bei der Optimierung der postoperativen Schmerztherapie in Ihrem Hause!

Copyright Nutzung durch Dritte vorbehaltlich Genehmigung durch DGAI/ DGCH Version 2018
