# Supplementary material for: The effect of adjuvant oral application of honey in the management of postoperative pain after tonsillectomy in adults: A pilot study
Source: PLoS One. 2020 Feb 10;15(2):e0228481. doi: 10.1371/journal.pone.0228481 (PMC7010464; doi:10.1371/journal.pone.0228481)
Supplement: S3 File — (DOCX) [file pone.0228481.s011.docx]

Summary

The main objective of QUIPS is to improve postoperative pain therapy by collecting data on pain treatment quality outcomes, analyzing them and sending an immediate feedback to the participating hospitals. As a sufficient monitoring of symptoms can contribute to reducing the length of stay - and thus increase patient satisfaction - the QUIPS project focuses on outcome quality from the patients' point of view.

[
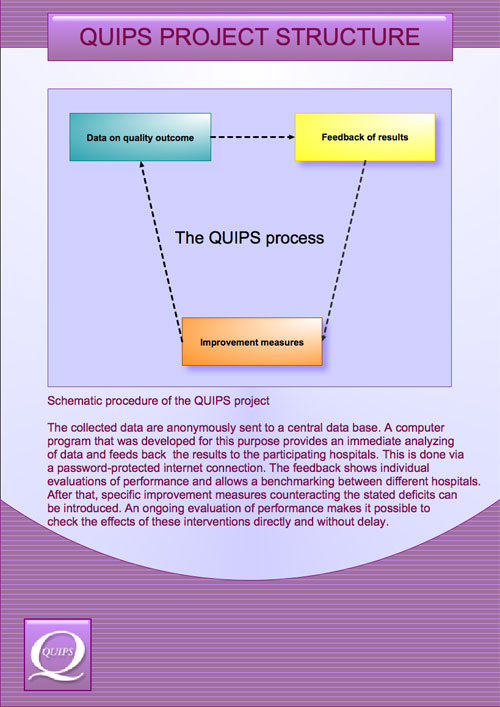
](http://www.quips-projekt.de/sites/quips/files/pictures/quips_project_structure.jpg)

**Way of proceeding**

On the first postoperative day, a bedside registration of several outcome quality parameters (pain intensity, functional impairments, side effects) as well as of selected clinical-demographic data takes place.

**Participation**

QUIPS can be carried out in operative centers of any size. Neither a minimum number of beds nor a broad variety of surgery is necessary. As a valid data collection is of utmost priority, the modular participation package includes a detailed training course and written guidelines on the standard operating procedures. The project was designed without industrial support and was funded by the German Federal Ministry of Health (BMGS) within the BIG project. The steering committees of the German association for anesthesiology and intensive care (DGAI) and of the professional association of German anesthesiologists (BDA) decided in 2005 to take over the patronage of this project, ensuring not only the project's continuity after the BMGS funding expired but also the setup of a Germany-wide benchmark project in postoperative pain treatment.
The German association for surgery (DGCH), the professional association of German surgeons (BDC), and the Austrian Society of Anesthesiology, Reanimation and Intensive Care (ÖGARI) are cooperating with QUIPS. Thus, a sustainable and non-commercial implementation of the project is ensured and an extensive platform for discussions on interdisciplinary clinical and nursing questions is made available.

Background and objectives

Postoperative pain is not only an unpleasant sensory experience but carries the risk of a number of potentially dangerous consequences. Severe pain can delay postoperative mobilisation and prolong rehabilitation. Postoperative pain increases endocrine stress reactions and has negative effects on wound healing. There is strong evidence that severe postoperative pain leads to chronic pain. For this reason, resolving pain according to state of the art standards should be a moral obligation for every healthcare provider.

Unfortunately, the quality outcome of postoperative pain therapy is far from being optimal in the clinical day-to-day business and is described: "as ineffective, inadequate and without the necessary organizational and scientific background" (Neugebauer 1998).

As pain therapy is not a particularly complex medical problem, the reason for this negative outcome is likely to be caused by the numerous non-medical obstacles that prevent an efficient implementation of pain therapy.

"Thus it appears that the solution of the problems of postoperative pain management lies not so much in the development of new techniques but in development of an organization to exploit existing expertise" (Rawal 1994).

QUIPS's main objective is to improve postoperative pain therapy by collecting data on pain treatment quality outcomes, analyzing them and sending an immediate feedback to the participating hospitals. This system works independently from the hospitals' infrastructural conditions (i.e. IT or documentation systems) and guarantees an ongoing quality assurance by its internal and external benchmarking.

Data collection

The collection of data consists of two parts: the obligatory collection of outcome parameters and the facultative collection of process parameters. At the beginning, data concerning the structure quality are also collected.

Outcome parameters

Outcome parameters - plus a few demographic data like age, gender, ward, operation - are registered by use of a questionnaire that is filled in by the patient at his bedside on the first postoperative day. This questionnaire was developed within the BMGS-project "Benchmarking im Gesundheitswesen" (benchmarking in public healthcare). It includes elements of the Brief Pain Inventory as well as of the Oucome Questionnaire developed by the American Pain Society. Special emphasis was put on a standardized way of collecting the data in order to minimize the variability of questioning interaction and to guarantee an interclinical comparability of data. Distribution, explication and collection of the questionnaires is for example done by a member of staff who is not directly involved in the patient's care. This is a requirement to make sure that the data are collected under the same conditions throughout Germany and that the SOPs are strictly observed. The time needed for collecting the outcome parameters is about 5 minutes per patient.

The outcome quality parameters are mainly defined from the patient's point of view. Priority is given to the functional impact of pain, to side effects of therapies and to patient satisfaction. After that, all anonymized data are transmitted to an external data base where they are analyzed.

Process parameters

On a facultative basis, additional process data can be collected (e.g. concerning anesthesia, surgery, pain therapy on the ward, etc.) in order to enable deficit analyzing. Furthermore, additional blank spaces permit to collect parameters that are of individual interest. For a conclusive feedback, at least 30 - better 50 - data sets per ward per quarter are necessary. This means that not all patients have to answer the questionnaire but only a randomized number of them. Uniform methods of randomization guarantee interclinical comparability and minimize the danger of selection bias and systematic errors.

At the moment, an interface with existing IT-systems in hospitals is not necessary because all outcome parameters are collected by the questionnaire on a daily basis. Prospectively, such an interface for integration of data (e.g. of anesthesia protocols or electronic patient files) is imaginable.

Feedback

Once collected, all data are anonymized and sent to the so-called benchmark server where participants can compare their own results with those of other hospitals. Filter functions enable the user to choose different parameters and to do stratifications according to different fields of medical treatment. At this, the user can identify his own data and compare them with other anonymized data sets. An automated data analyzing allows a prompt, nearly realtime feedback (fig. 1 and 2)

[
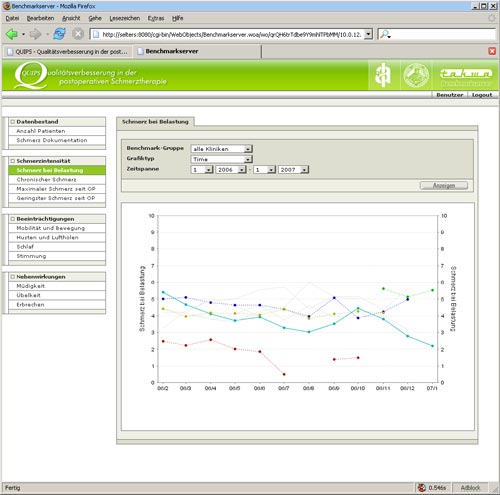
](http://www.quips-projekt.de/sites/quips/files/pictures/feedback_en_1.jpg)
Fig. 1: Online presentation "Patients with side-effects"

Additionally, the performance in the course of time can be shown. This feature permits the assessment of effects caused by modifications of procedures (fig. 1). Frequently, changes of "own" data are more likely to be accepted than comparisons with other institutions.

[
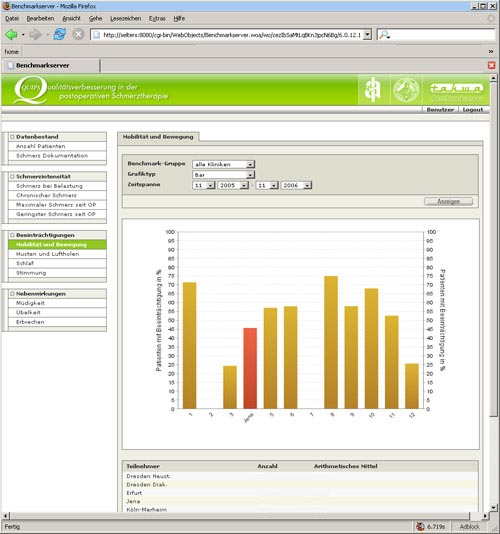
](http://www.quips-projekt.de/sites/quips/files/pictures/feedback_en_2.jpg)
Fig. 2: Online presentation "Patients with handicaps"

Because of the considerable number of data sets, an offline evaluation of many additional aspects is possible (e.g. surgery-specific problems, influences of different anesthesia procedures or use of analyses of variance in order to identify independent factors of influence on the outcome).
The identification of so-called tracer-operations that are characteristic for a particular field of medicine (e.g. cholezystectomy for abdominal surgery) provides an additional reduction of variables and thus an even more valid comparison of different hospitals as well as a further economization of data collection.
Those offline-evaluations can be coordinated by the steering committee of the DGAI. Furthermore, each participant can use his own data sets without any restrictions for evaluation purposes.
